# TRIM21 blocks DDX3X-driven stress granule formation during *Mycobacterium tuberculosis* infection via a non-canonical E3 ligase mechanism

**DOI:** 10.1371/journal.ppat.1014553

**Published:** 2026-09-08

**Authors:** Ruiyao Xu, Mengru Lu, Gaoyan Wang, Ximeng Zhang, Yuxing Huang, Yu Wang, Qing Yu, Yaxuan Wang, Dan Chen, Yunlong Hu, Chenyan Shi, Yi Cai, Li Fu, Xinchun Chen, Youchao Dai

**Affiliations:** 1 Guangdong Provincial Key Laboratory of Infection Immunity and Inflammation, Department of Pathogen Biology, School of Medicine, Shenzhen University, Shenzhen, China; 2 Guangzhou Medical Research Institute of Infectious Diseases, Infectious Disease Center, Guangzhou Eighth People’s Hospital, Guangzhou Medical University, Guangzhou, Guangdong, China; 3 Guangdong Provincial Key Laboratory of Regional Immunity and Diseases, Department of Pharmacology and International Cancer Center, School of Medicine, Shenzhen, China; Centre National de la Recherche Scientifique, FRANCE

## Abstract

Tripartite motif-containing 21 (TRIM21) is an E3 ubiquitin ligase that binds viral and host proteins to mediate antiviral defense. Its role in *Mycobacterium tuberculosis* (*Mtb*) infection, however, is unclear. Here, our analysis of human TB transcriptomic datasets showed that TRIM21 expression levels correlate with TB progression in human patients. We further demonstrated that TRIM21 deficiency suppresses *Mtb* growth in both macrophages and a murine model of tuberculosis (TB). Mechanistically, TRIM21 limits host resistance to *Mtb* through a non-canonical pathway distinct with its E3 ligase activity. It physically interacts with the RNA helicase DEAD-box helicase family member DDX3X and disrupts phase separation dynamics required for stress granule (SG) assembly. Impaired SG formation compromises innate immune responses, thereby facilitating intracellular bacterial growth and creating a permissive cellular environment for *Mtb* growth. Consistent with these genetic findings, pharmacological inhibition of TRIM21 restricted *Mtb* growth in experimental macrophage and murine models. Together, these findings uncover a non-canonical role of TRIM21 in *Mtb* infection and highlight its potential as a therapeutic target for host-directed therapy against TB.

## Author summary

Tuberculosis (TB) remains one of the world’s most serious infectious diseases, and better treatments are urgently needed to improve outcomes for patients. Because host immunity plays a critical role in controlling *Mycobacterium tuberculosis* (*Mtb*) infection, host-directed therapy (HDT), which enhances protective host responses, has emerged as a promising complementary approach.

In this study, we identify TRIM21 as a host factor that promotes intracellular *Mtb* growth and increases susceptibility to TB in mice through a mechanism distinct from its well-characterized E3 ubiquitin ligase activity. We found that TRIM21 interacts with the RNA helicase DDX3X, thereby disrupting liquid–liquid phase separation dynamics required for stress granules, which are cellular structures that help organize protective responses during infection. Importantly, we found that blocking TRIM21 activity enhances host resistance to *Mtb* infection in both cellular and animal models. Our findings uncover a previously unrecognized TRIM21-mediated mechanism by which *Mtb* evades innate immune defenses and position TRIM21 as a potential target for HDT in TB.

## Introduction

Tuberculosis (TB), caused by *Mycobacterium tuberculosis* (*Mtb*), is a leading cause of mortality from infectious disease worldwide [1]. While drug-sensitive TB can be treated with 6-month antibiotic regimens, drug-resistant strains pose a major clinical challenge. In addition, shorter treatment courses are urgently needed to improve patient adherence, which remains a major driver of therapeutic failure and resistance development. Because host immunity plays a central role in shaping the outcome of *Mtb* infection, host-directed therapy (HDT), which aims to enhance protective host responses rather than directly targeting the pathogen, has emerged as a complementary strategy to improve TB treatment [2,3].

Tripartite motif-containing protein 21 (TRIM21), also known as RO52, is a well-characterized target of circulating autoantibodies in autoimmune diseases [4]. TRIM21 is an E3 ubiquitin ligase that directly binds viral and host proteins during the antiviral defense [5–7], and is a negative regulator of innate immune responses during certain bacterial infections, where it recognizes antibody-coated Salmonella and promotes cell death [8]. Our previous work demonstrated that TRIM21 functions as an E3 ubiquitin ligase to promote HERC2- and NCOA4-dependent ferritin degradation, thereby supporting intracellular *Mtb* growth [9,10]. Interestingly, members of the TRIM protein family are also recognized to perform non-canonical, non-enzymatic functions through protein–protein interactions and modulation of cellular signaling networks, including epigenetic regulation [11]. Together, these findings suggest that TRIM21 may exert broad immunoregulatory effects during infection. However, a complete picture of TRIM21’s role in mediating *Mtb* infection has not been defined.

In this study, we sought to fully elucidate TRIM21’s role during *Mtb* infection and to identify potential non-canonical functions distinct with its E3 ligase activity. We first demonstrated that TRIM21 deficiency suppresses *Mtb* growth in both macrophages and a murine model. We further revealed that TRIM21 interacts with the DEAD-box helicase family member DDX3X and disrupts liquid-liquid phase separation (LLPS) dynamics, thereby inhibiting stress granule (SG) assembly—a process critical for anti-pathogen defense [12]. Finally, using both genetic models and the inhibitor fimepinostat, we intended to validate TRIM21 as a candidate target for anti-TB therapy in both macrophages and in vivo murine models. Our findings provide compelling evidence for the essential role of TRIM21 in *Mtb* infection and highlight its potential as a therapeutic target for host-directed tuberculosis treatment.

## Results

### TRIM21 deficiency enhances host resistance to *Mtb* infection

**Fig 1:**
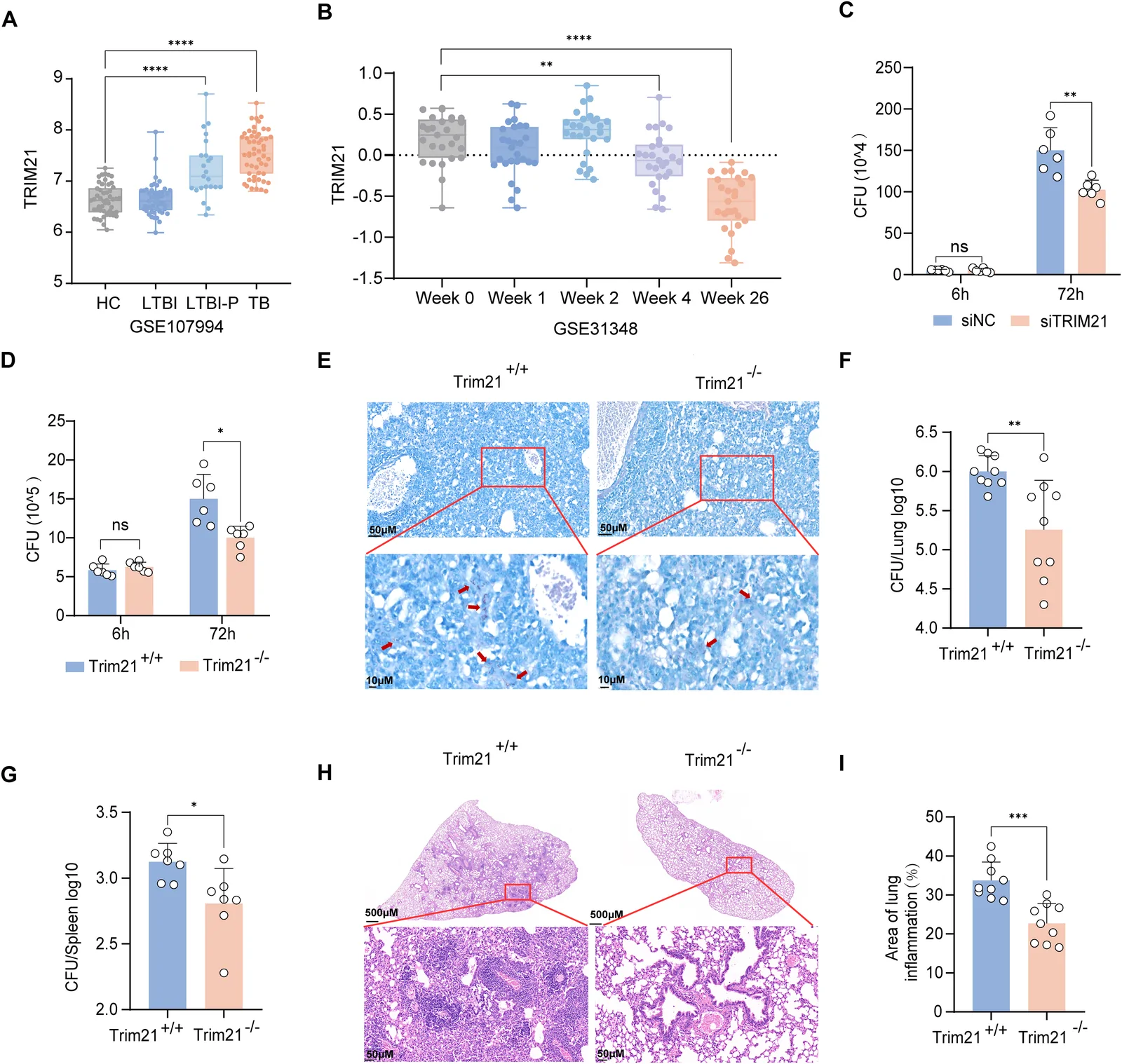
TRIM21 deficiency further enhances clearance of *Mtb* in macrophages and mice. **(A)** TRIM21 mRNA expression in peripheral blood from healthy controls (HC), individuals with latent TB infection (LTBI), and patients with active TB, based on the GSE107994 dataset. LTBI-progressor (LTBI-P) refers to individuals with latent tuberculosis infection who subsequently progressed to active tuberculosis disease during follow-up. **(B)** Longitudinal analysis of TRIM21 mRNA expression before and after anti-TB therapy at the indicated time points, based on the GSE31348 dataset; each dot represents one individual sample. **(C)** Intracellular *Mycobacterium tuberculosis* H37Rv colony-forming units (CFUs) in TRIM21-knockdown THP-1–derived human macrophages at 6h and 72h post-infection. **(D)** Intracellular H37Rv CFUs in bone marrow–derived macrophages (BMDMs) obtained from Trim21^+/+^ and Trim21^-/-^ mice. **(E)** Representative acid-fast–stained lung sections from Trim21^+/+^ and Trim21^-/-^ mice following aerosol infection with H37Rv from Trim21^+/+^ and Trim21^-/-^mice. **(F-G)** Trim21^+/+^ and Trim21^-/-^ mice were aerosol-infected with approximately 200 CFU of H37Rv. Bacterial burdens in the lungs (F) and spleens (G) were quantified by CFU enumeration at 5 weeks post-infection. (H-I) Representative lung sections stained with hematoxylin and eosin (H&E) for histopathological assessment **(H)**, with quantification of immune cell infiltration **(I)**. Data are presented as means ± SD. Statistical significance was assessed using Student’s *t* test. *P < 0.05, **P < 0.01, ***P < 0.001, ****P < 0.0001.

In our previous work, we observed upregulation of both TRIM21 mRNA and protein levels in THP-1–derived macrophages during *Mtb* infection [10]. To assess the clinical relevance of these findings, we analyzed TRIM21 mRNA expression patterns across multiple human TB cohorts. Transcriptomic data from the GEO database showed that TRIM21 expression was increased in patients with active TB compared with uninfected individuals and those with latent TB infection (Fig 1A, S1 Fig A, B) [13,14]. Given the heightened susceptibility to TB in HIV-positive populations, we also examined co-infected cohorts and observed higher TRIM21 expression in HIV-positive individuals with active TB than in those with latent infection (S1 Fig C) [15]. Moreover, treatment-response analyses showed that TRIM21 expression declined markedly following successful *Mtb* clearance after both 12-week and 26-week treatment regimens (Fig 1B, S1 Fig D) [16,17].

To determine whether this induction functionally influences intracellular bacterial growth, we first assessed the impact of TRIM21 depletion on *Mtb* growth in THP-1-derived macrophages. TRIM21 knockdown using siRNA enhanced bacterial clearance compared with control cells (Fig 1C, S1 Fig E, F). Consistently, Trim21-deficient bone marrow-derived macrophages (BMDMs) exhibited a greater capacity to clear *Mtb* than wild-type BMDMs (Fig 1D). We next examined whether TRIM21 similarly modulates host resistance in vivo. In murine infection models, TRIM21-deficient mice showed lower bacterial burdens in both the lungs and spleens, together with reduced immune cell infiltration, compared with wild-type animals (Fig 1E–I, S1 Fig G, H). Collectively, these data indicate that elevated TRIM21 expression is associated with TB disease progression and is inversely correlated with effective bacterial clearance, thereby providing a strong rationale for further delineating the molecular mechanisms by which TRIM21 modulates host–pathogen interactions during *Mtb* infection.

### TRIM21 interacts with DDX3X

**Fig 2:**
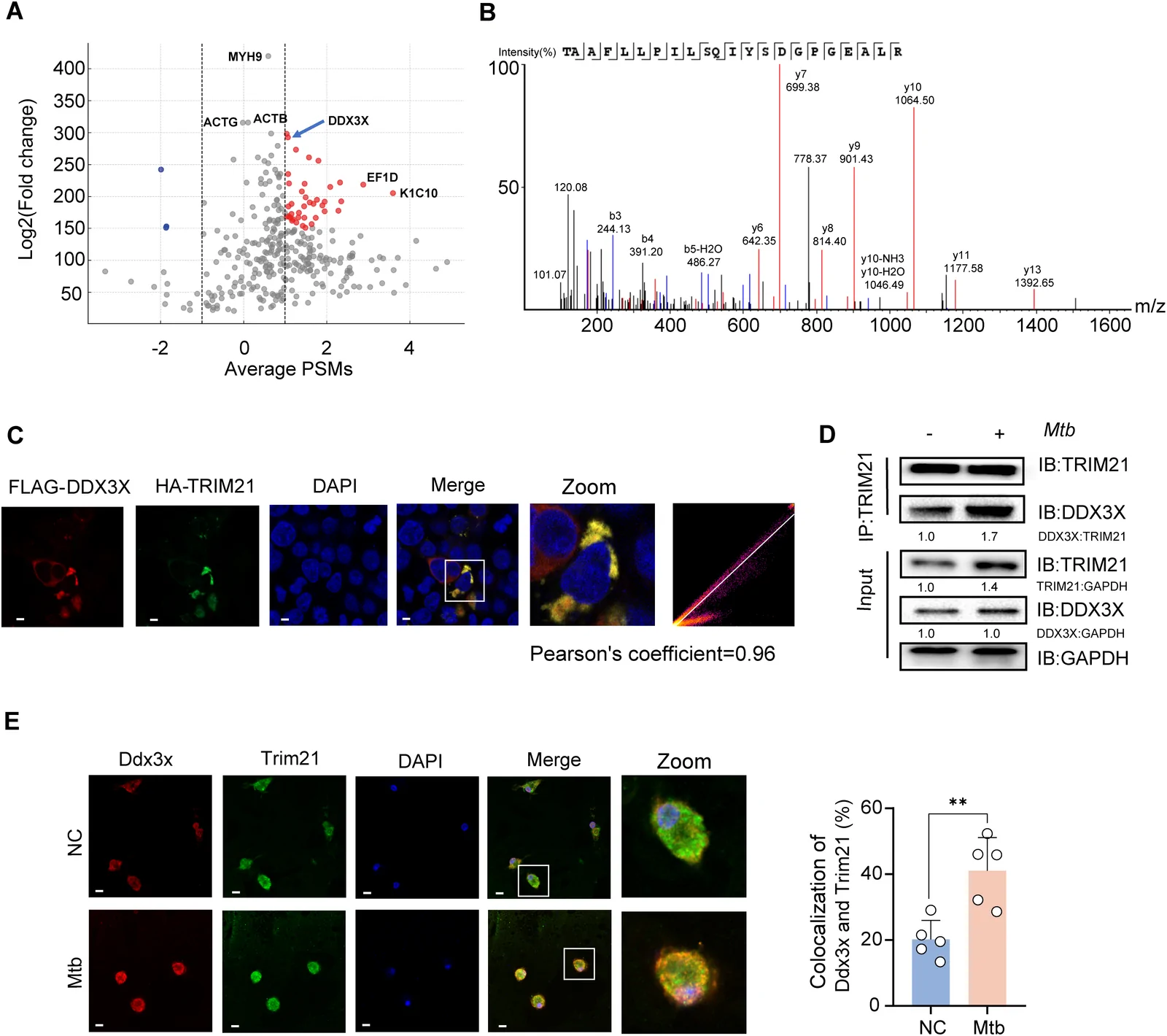
TRIM21 interacts with DDX3X. **(A)** Volcano plot showing proteins captured by anti-TRIM21 immunoprecipitation in THP-1 macrophages under uninfected conditions or following *Mycobacterium tuberculosis* (*Mtb*) infection. The x-axis indicates the log_2_ ratio of normalized scores for infected versus control samples, and the y-axis shows the average number of peptide-spectrum matches (PSMs). **(B)** Representative MS2 spectrum of a DDX3X-derived peptide identified by mass spectrometry. **(C)** Representative confocal images of 293T cells co-expressing FLAG-DDX3X and HA-TRIM21, showing colocalization of the two proteins (scale bars: 5 μm). Colocalization was analyzed with the ImageJ software and shown in the right panels. **(D)** Co-immunoprecipitation analysis of endogenous TRIM21 and DDX3X in THP-1 macrophages infected with H37Rv for 24 h (MOI = 5). **(E)** Representative images of mouse peritoneal macrophages stained for Trim21 and Ddx3x (scale bars: 5 μm). Colocalization was analyzed with the ImageJ software and shown in the right panel. Statistical significance was assessed using Student’s *t* test. **P < 0.01.

To elucidate the mechanism by which TRIM21 modulates *Mtb* infection, we performed mass spectrometric analysis of proteins captured by anti-TRIM21 co-immunoprecipitation (co-IP) in THP-1 macrophages under both uninfected and *Mtb*-infected conditions (S1 Table). Among the identified candidates, we prioritized DDX3X, a key regulator in innate immunity, for further investigation as it showed enrichment in the TRIM21 interactome during *Mtb* infection (Fig 2A, 2B). Confocal microscopy analysis of 293T cells co-expressing FLAG-DDX3X and HA-TRIM21 revealed colocalization of the two proteins (Fig 2C). Moreover, endogenous co-IP and confocal microscopy analysis demonstrated that the TRIM21–DDX3X interaction was markedly enhanced during *Mtb* infection (Fig 2D, 2E).

### TRIM21 negatively regulates stress granules formation after *Mtb* infection

Stress granules (SGs) are important modulators of immune responses and a conserved host defense mechanism during microbial infection [12]. During *Mtb* infection, they form protective plugs at damaged endolysosomal membranes that repair *Mtb*-containing phagosomes and restrict bacterial growth [18]. Interestingly, DDX3X rapidly aggregates in SGs under cellular stress, facilitating protein recruitment for granule assembly and dynamics [19].

**Fig 3:**
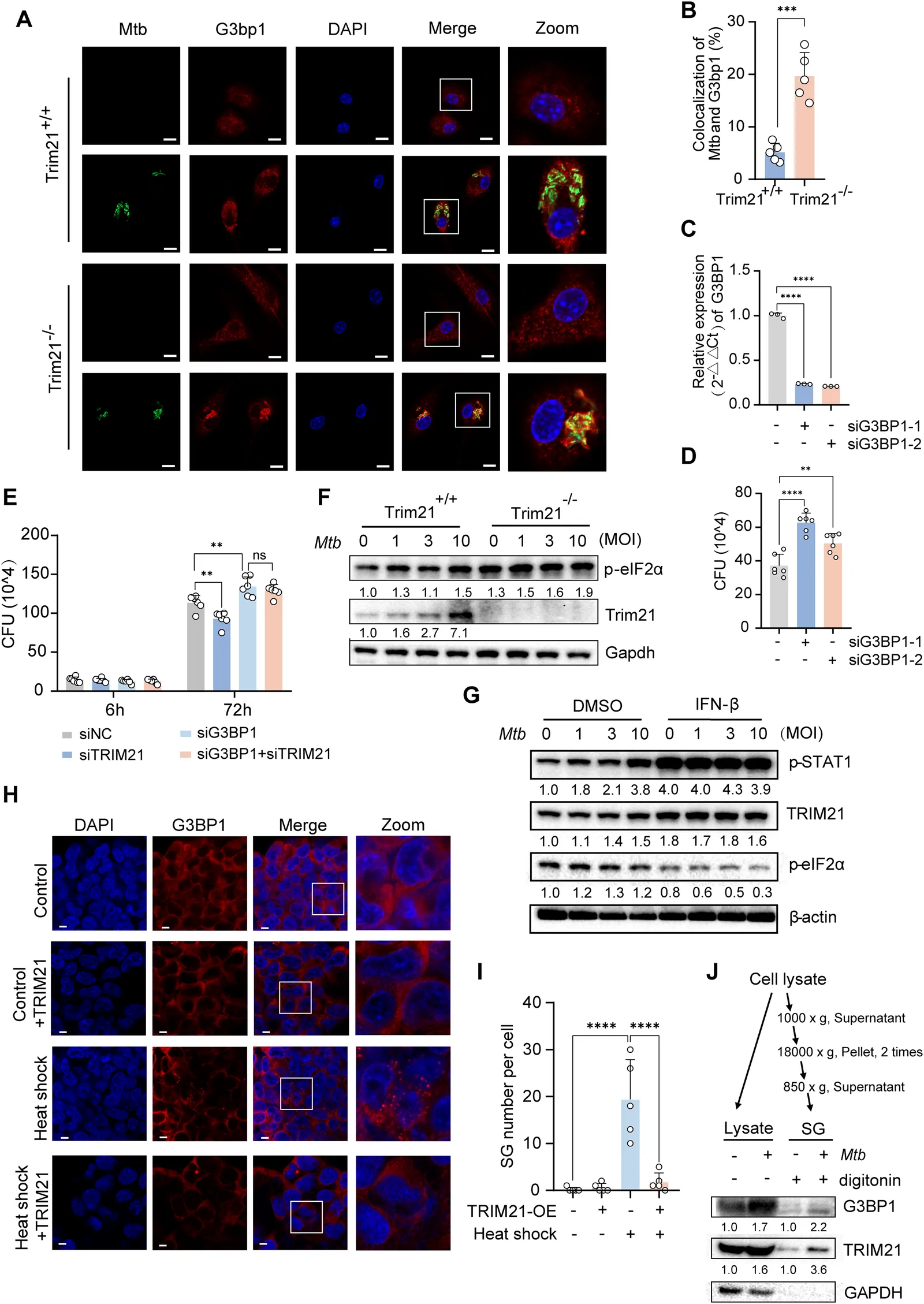
TRIM21 negatively regulates SG formation after *Mtb* infection. **(A)** Representative confocal images of peritoneal macrophages infected with *Mycobacterium tuberculosis* (*Mtb*) (MOI = 5, 48 h) and stained for the stress granule marker G3BP1 (scale bars: 10 μm). **(B)** Colocalization of *Mtb* and G3bp1 was analyzed with the ImageJ software. **(C-D)** RT–qPCR analysis of G3BP1 knockdown efficiency in THP-1–derived macrophages, and intracellular H37Rv colony-forming units (CFUs) in G3BP1-knockdown THP-1–derived macrophages. **(E)** Intracellular H37Rv CFUs in TRIM21-knockdown and G3BP1-knockdown THP-1–derived macrophages. **(F)** Immunoblot analysis of phosphorylated elF2α and Trim21 in Trim21^+/+^ and Trim21^−/−^ BMDMs after *Mtb* infection. **(G)** Immunoblot analysis of phosphorylated eIF2α, phosphorylated STAT1 and Trim21 in THP-1 cells following *Mtb* infection with simultaneous IFN-β treatment (100 units/ml). **(H)** Immunofluorescence analysis of G3BP1 in 293T cells transfected with a TRIM21 expression plasmid and subjected to heat shock (scale bars: 5 μm). **(I)** Quantification of G3BP1 (per cell) in 293T cells transfected with a TRIM21 expression plasmid and subjected to heat shock. **(J)** Immunoblot analysis of TRIM21 in stress granule–enriched fractions following *Mtb* infection. Data in bar graphs are presented as means ± SD. Statistical significance was assessed using Student’s *t* test and one-way ANOVA. **P < 0.01, ***P < 0.001, ****P < 0.0001.

In this study, we confirmed that *Mtb* localized to SGs in infected macrophages. Notably, compared with wild-type macrophages, Trim21-deficient macrophages exhibited significantly increased co-localization of *Mtb* with SGs (Fig 3A, 3B). In addition, TRIM21 deficiency is associated with reduced Galectin-3 recruitment to *Mtb*-containing compartments, indicating decreased lysosomal membrane damage, together with a reduced intracellular *Mtb* burden. These observations are consistent with the enhanced antibacterial phenotype observed in TRIM21-deficient macrophages (S2 Fig A-D). Moreover, knockdown of the SG core component G3bp1 enhanced intracellular *Mtb* growth in macrophages (Fig 3C, 3D), supporting a protective role for SGs during infection.

To determine whether the effect of TRIM21 on *Mtb* growth is dependent on SG formation, we genetically impaired SG assembly by targeting key SG components G3bp1 and assessed intracellular bacterial in control and TRIM21-deficient macrophages. Notably, Trim21-deficient cells exhibited a significant reduction in intracellular *Mtb* burden compared with control cells. Notably, this difference was abolished upon disruption of SG assembly, indicating that intact SG formation is required for the TRIM21-dependent regulation of *Mtb* growth (Fig 3E).

SG assembly is frequently initiated by stress-induced eIF2α phosphorylation, which promotes translational arrest and the accumulation of untranslated mRNAs into SGs [20]. We also observed that TRIM21-deficient macrophages exhibited significantly elevated phosphorylated eIF2α levels compared with controls (Fig 3F). TRIM21 has been reported to be upregulated upon activation of the type I interferon (IFN-I)–STAT1 signaling axis [8,21]. Upon exogenous IFN-β treatment, activation of the type I interferon signaling pathway led to a marked increase in TRIM21 expression, while SG formation was reduced under these conditions (Fig 3G). Furthermore, TRIM21 overexpression suppressed SG formation in heat-shocked 293T cells (Fig 3H, 3I). In addition, TRIM21 protein was substantially enriched in the SG-enriched fraction following *Mtb* infection (Fig 3J), suggesting an association between TRIM21 and SG-containing complexes during infection.

Together, these data indicate that TRIM21 acts as a negative regulator of SG formation during *Mtb* infection, thereby impairing a host defense mechanism that restricts intracellular bacterial growth.

### TRIM21 blocks the DDX3X-G3BP1 interaction by modulating DDX3X phase separation

We next hypothesized that TRIM21 could potentially regulate DDX3X through ubiquitin-mediated proteasomal degradation. However, TRIM21 knockdown in THP-1–derived macrophages did not alter DDX3X protein levels (S3 Fig A–C). To further exclude the possibility of proteasomal involvement, we treated cells with protein synthesis inhibitor cycloheximide (CHX) and proteasome inhibitor MG132. In the presence of CHX, DDX3X protein levels were dramatically decreased and MG132 treatment did not recover DDX3X protein levels (S3 Fig D). These data suggested that downregulation of DDX3X protein occurs through a proteasome-independent mechanism.

**Fig 4:**
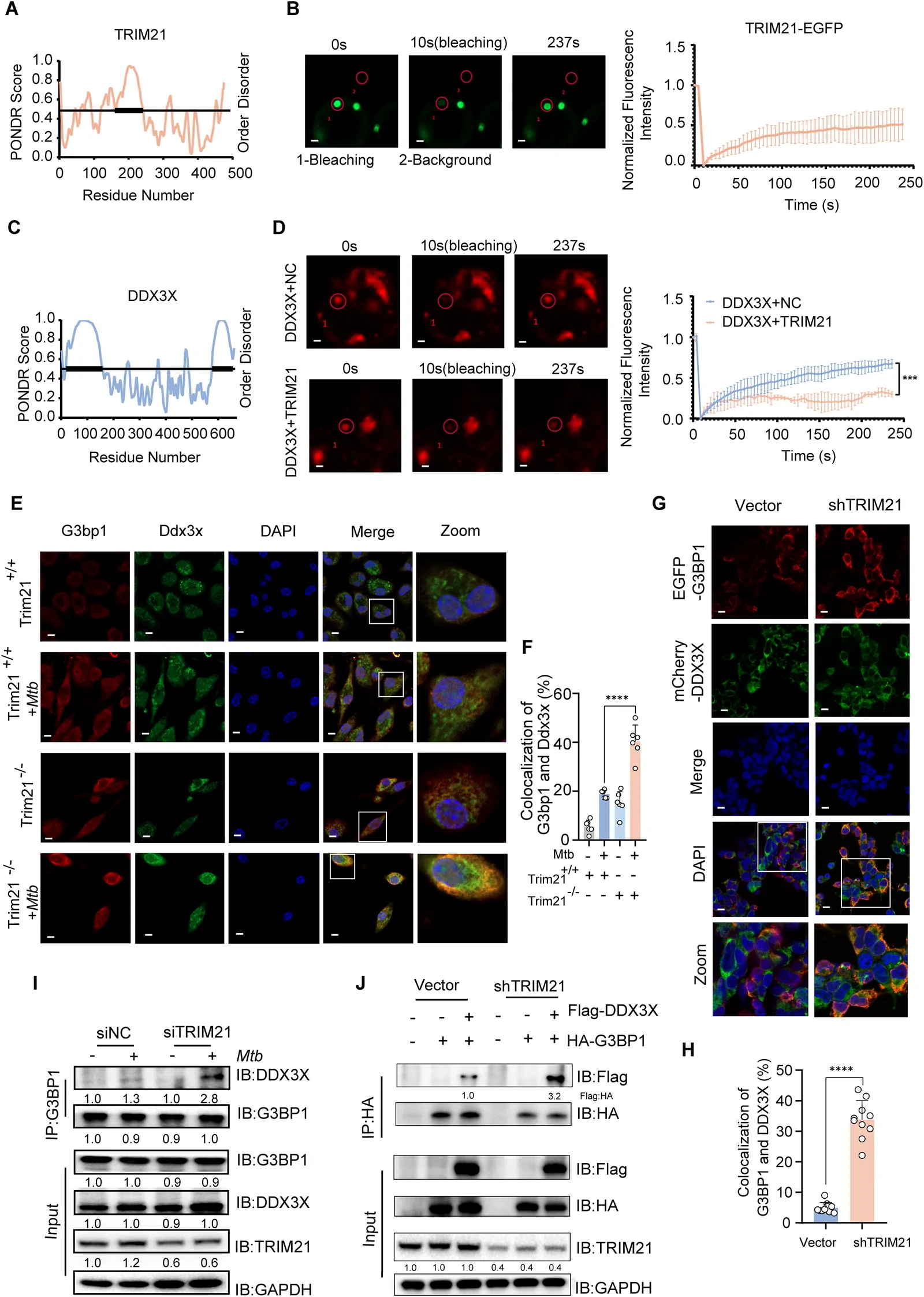
TRIM21 regulates the phase separation behavior of DDX3X and its interaction with G3BP1. **(A)** Prediction of intrinsically disordered regions (IDRs) in TRIM21 using PONDR analysis. **(B)** Fluorescence recovery after photobleaching (FRAP) analysis of EGFP–TRIM21 condensates in 293T cells, including representative images and recovery quantification (scale bars: 5 μm). **(C)** Prediction of IDRs in TRIM21 using PONDR analysis. **(D)** FRAP analysis of mCherry–DDX3X condensates in 293T cells, including representative images and recovery quantification (scale bars: 5 μm). **(E)** Immunofluorescence analysis of G3BP1 and DDX3X colocalization in Trim21^+/+^ and Trim21^-/-^ peritoneal macrophages following *Mtb* infection (scale bars: 5 μm). **(F)** Colocalization of Ddx3x and G3bp1 was analyzed with the ImageJ software. **(G)** Representative confocal images of 293T cells co-transfected with EGFP–G3BP1 and mCherry–DDX3X plasmids under control conditions or following TRIM21 knockdown. (scale bars: 10 μm). **(H)** Colocalization of DDX3X and G3BP1 was analyzed with the ImageJ software. **(I)** Co-immunoprecipitation analysis of endogenous G3BP1 and DDX3X in TRIM21-knockdown THP-1–derived macrophages during *Mtb* infection. **(J)** Co-immunoprecipitation analysis of FLAG–DDX3X and HA–G3BP1 in 293T cells transfected with control or TRIM21-targeting shRNA. Data in bar graphs are presented as means ± SD. Statistical significance was assessed using Student’s t test. ****P < 0.0001.

DDX3X plays a central role in cellular phase separation, particularly during SG formation in response to oxidative stress or heat shock [22,23]. Phase separation is driven by intrinsically disordered regions (IDRs) that enable multivalent interactions with RNA or partner proteins to promote droplet-like condensate formation under specific cellular conditions [24]. Interestingly, we identified IDRs in both TRIM21 and DDX3X, with the TRIM21 IDR localized within its coiled-coil domain and the DDX3X IDR localized within its N-terminal and C-terminal extensions (Fig 4A, 4C). These data for TRIM21 are consistent with previous studies implicating the coiled-coil domain in protein condensation and autophagosome formation via phase separation [25,26].

To investigate the condensate formation properties of TRIM21, we expressed EGFP–TRIM21 in 293T cells. Fluorescence recovery after photobleaching (FRAP) assays showed rapid recovery of EGFP–TRIM21 within 300 seconds, consistent with the dynamic nature observed in many biomolecular condensates (Fig 4B, S1 Video). Importantly, TRIM21 altered the dynamics and molecular mobility of DDX3X condensates: In cells co-expressing DDX3X and TRIM21, FRAP analysis revealed reduced fluorescence recovery of DDX3X compared with cells expressing DDX3X and an empty vector, indicating decreased molecular mobility within DDX3X condensates (Fig 4D, S2 and S3 Videos). These findings suggest that TRIM21 influences the dynamics and mobility of DDX3X condensates, likely through co-condensation with DDX3X.

Given that DDX3X interacts with G3BP1 under conditions of cellular stress [22,23], we examined whether TRIM21 modulates this interaction in different cellular contexts. Immunofluorescence analysis showed enhanced G3bp1–Ddx3x colocalization in peritoneal macrophages from Trim21-deficient mice compared with wild-type controls (Fig 4E, 4F). This effect was recapitulated in vitro in 293T cells, where TRIM21 knockdown increased DDX3X–G3BP1 colocalization (Fig 4G, 4H). Consistently, co-IP assays demonstrated strengthened endogenous G3BP1–DDX3X interactions in Trim21-deficient THP-1 macrophages during *Mtb* infection (Fig 4I), which was again confirmed in 293T cells co-expressing FLAG–DDX3X and HA–G3BP1 (Fig 4J). Collectively, these data indicate that TRIM21 negatively regulates the DDX3X–G3BP1 interaction during SG assembly.

### The TRIM21 coiled-coil domain binds DDX3X to facilitate intracellular *Mtb* growth

**Fig 5:**
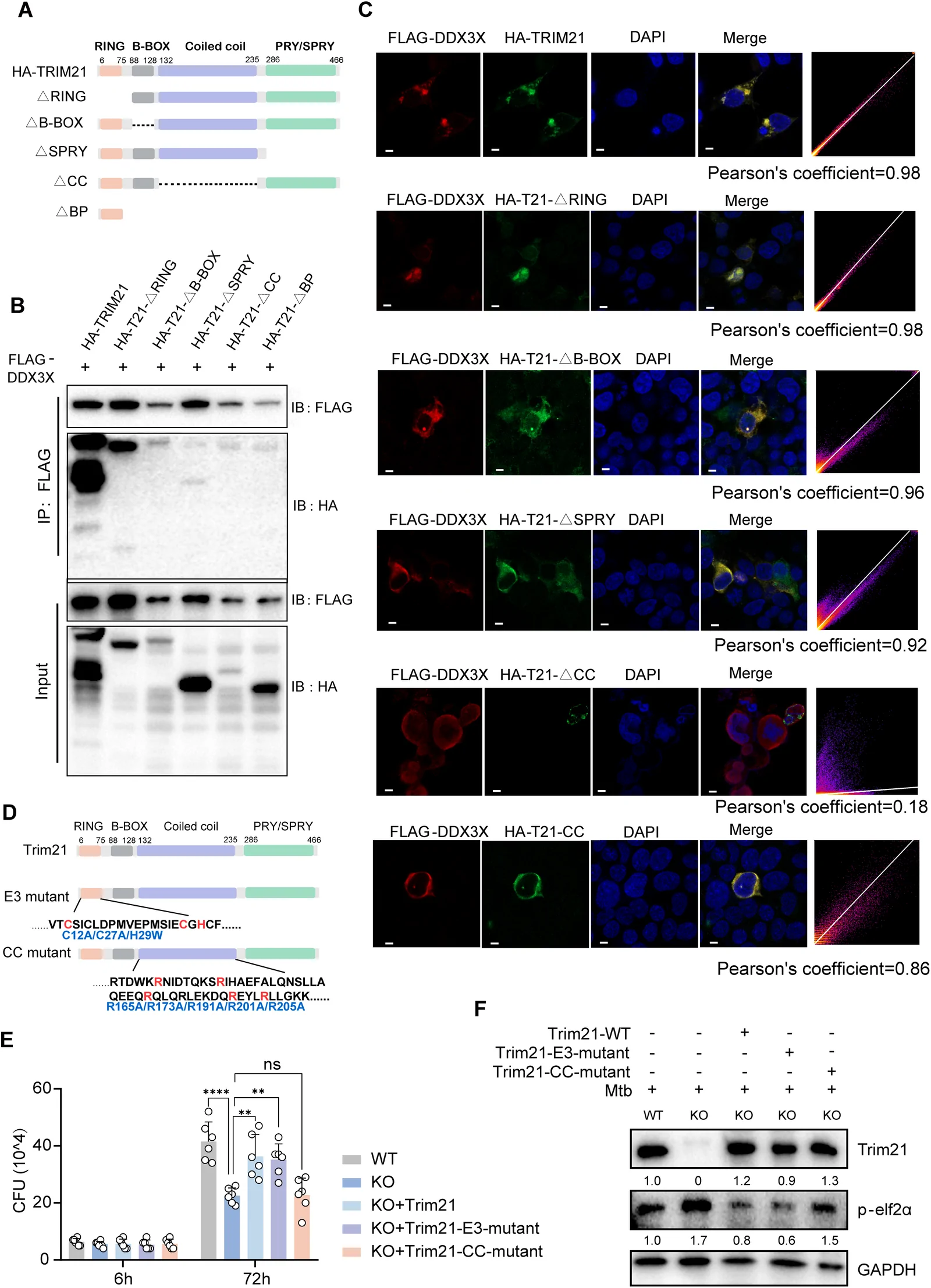
The TRIM21 coiled-coil domain is required for DDX3X interaction and intracellular *Mtb* growth. **(A)** Schematic representation of TRIM21 truncation mutants used to map the DDX3X interaction domain. **(B)** Co-immunoprecipitation analysis of FLAG–DDX3X with HA-tagged full-length TRIM21 or TRIM21 truncation mutants expressed in 293T cells. **(C)** Representative confocal images of 293T cells co-expressing FLAG–DDX3X (red) and the indicated HA-tagged TRIM21 truncation mutants (green), showing colocalization patterns (scale bars: 5 μm). Colocalization was analyzed with the ImageJ software and shown in the right panels. **(D)** Schematic representation of TRIM21 RING-domain and coiled-coil–domain point mutants used for functional reconstitution experiments. **(E)** Intracellular H37Rv colony-forming units (CFUs) in wild-type (WT) bone marrow–derived macrophages (BMDMs), Trim21-knockout (KO) BMDMs, and Trim21-KO BMDMs reconstituted with wild-type TRIM21 or the indicated TRIM21 domain mutants at the indicated time points. **(F)** Immunoblot analysis of phosphorylated eIF2α and TRIM21 in Trim21- KO BMDMs and Trim21-KO BMDMs reconstituted with wild-type TRIM21 or the indicated TRIM21 domain mutants. Data are presented as means ± SD. Statistical significance was assessed using one-way ANOVA. *P < 0.05, ***P < 0.001, ****P < 0.0001. ns., not significant.

To define the structural basis of the TRIM21–DDX3X interaction, we generated a series of TRIM21 truncation mutants (Fig 5A) and assessed their interactions with DDX3X by co-IP. Mutants lacking the RING, B-box, or PRY/SPRY domains retained their ability to bind DDX3X. By contrast, deletion of the coiled-coil domain (ΔCC), or simultaneous deletion of the B-box, coiled-coil, and PRY/SPRY domains (ΔBP), markedly impaired the interaction with DDX3X (Fig 5B, 5C). These results indicate that TRIM21 engages DDX3X primarily through its coiled-coil domain. Consistent with this interaction, full-length TRIM21 overexpression suppressed SG formation induced by sodium arsenite (NaAsO_2_, a classical oxidative stress inducer that promotes SG assembly) in 293T cells, whereas a TRIM21 mutant lacking the coiled-coil domain failed to inhibit SG assembly (S4 Fig A).

To determine which TRIM21 domain is required to modulate intracellular *Mtb* growth, we generated TRIM21 mutants harboring point mutations in either the RING domain (to control for E3 ligase activity) or the coiled-coil domain. Arginine (R) and lysine residues, which are enriched in most cellular condensates, have markedly distinct propensities to drive the LLPS of protein/RNA mixtures [27,28]. Phase separation was abrogated when arginine-mediated interactions, including potential cation-π interactions, were disrupted by replacement of arginines with alanine (R165A/R173A/R191A/R201A/R205A) (Fig 5D). In this study, expression of the coiled-coil domain mutant in Trim21-deficient BMDMs failed to restore the inhibitory effect of TRIM21 on *Mtb* clearance, resulting in enhanced bacterial clearance compared with cells expressing wild-type TRIM21 or the RING domain mutant (Fig 5E). Similarly, expression of the coiled-coil domain mutant failed to rescue the SG-suppressive effect of TRIM21, whereas wild-type TRIM21 and the RING domain mutant reduced SG formation in Trim21-deficient BMDMs (Fig 5F).

Our previous work demonstrated that TRIM21 functions as an E3 ubiquitin ligase to promote HERC2- and NCOA4-dependent ferritin degradation, thereby supporting intracellular *Mtb* growth. Notably, even upon simultaneous knockout of NCOA4, a key regulator of ferritinophagy, TRIM21 depletion still reduced intracellular *Mtb* burden in THP-1–derived macrophages (S4 Fig B), suggesting that TRIM21 may regulate *Mtb* infection through additional NCOA4-independent mechanisms.

These findings indicate that the coiled-coil domain mediates TRIM21–DDX3X interaction and promotes intracellular *Mtb* growth through a mechanism distinct with TRIM21’s canonical E3 ubiquitin ligase activity. Moreover, these data support a central role for the coiled-coil domain in TRIM21-dependent suppression of SG formation during *Mtb* infection.

### Phenocopying TRIM21 loss with fimepinostat suppresses intracellular *Mtb* growth

**Fig 6:**
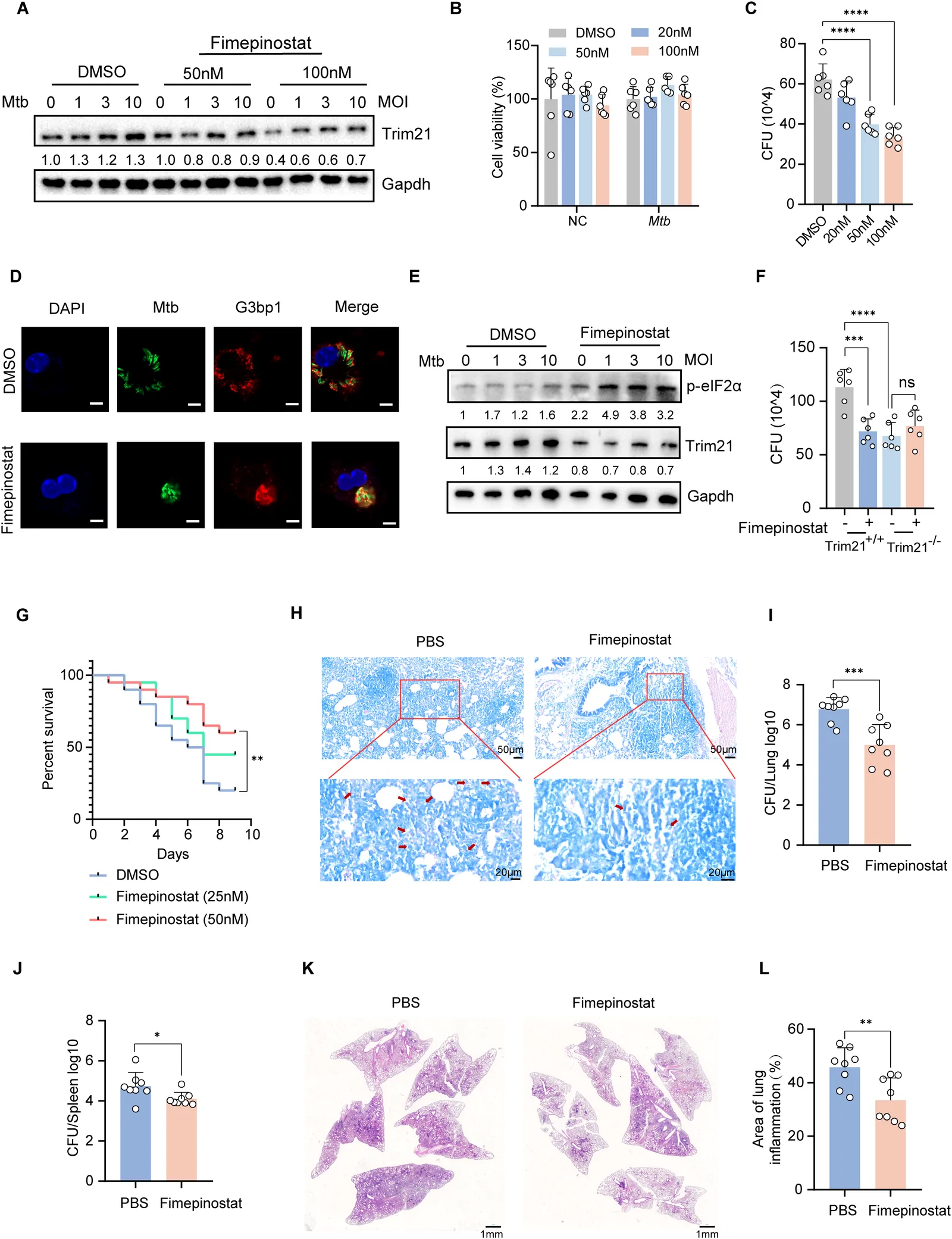
Pharmacological inhibition of TRIM21 by fimepinostat restricts intracellular *Mtb* growth in vitro and in vivo. **(A)** Immunoblot analysis of TRIM21 protein levels in *Mtb*-infected bone marrow-derived macrophages (BMDMs) treated with fimepinostat at the indicated concentrations and multiplicities of infection (MOI). Cells were pretreated with Fimepinostat for 2 h before *Mtb* infection and maintained under continuous exposure throughout the 24 h infection period. GAPDH served as a loading control. **(B)** Cell viability of uninfected (NC) and *Mtb*-infected BMDMs following treatment with fimepinostat at the indicated concentrations for 24h, as assessed by LDH assay. **(C)** Intracellular H37Rv colony-forming units (CFUs) in BMDMs treated with fimepinostat at the indicated concentrations. **(D)** Representative confocal images of *Mtb*-infected peritoneal macrophages (MOI = 5, 48 h) treated with fimepinostat (50 nM) and stained for the stress granule marker G3bp1 (scale bars: 10 μm). **(E)** Immunoblot analysis of phosphorylated elF2α and Trim21 in *Mtb*-infected BMDMs treated with fimepinostat (50 nM). **(F)** Intracellular H37Rv CFUs in BMDMs treated with fimepinostat (50 nM) in Trim21^+/+^ and Trim21^-/-^ BMDMs. **(G)** Survival of adult zebrafish (n = 20) following intraperitoneal infection with *Mycobacterium marinum* (500 CFU per fish) and daily treatment with fimepinostat (25 nM or 50 nM) or vehicle control (DMSO) for 9 days. **(H)** Representative acid-fast–stained lung sections from H37Rv-infected mice treated with vehicle control or fimepinostat. Red arrows indicate acid-fast bacilli. **(I, J)** Bacterial burden in the lungs (I) and spleens (J) of H37Rv-infected mice treated with phosphate-buffered saline (PBS) or 35 mg/kg fimepinostat, as determined by CFU enumeration. **(K)** Representative hematoxylin and eosin **(H&E)**–stained lung sections from H37Rv-infected mice treated with PBS or fimepinostat. **(L)** Quantification of lung immune cell infiltration area from H&E-stained sections. Data are presented as means ± SD. Statistical significance was assessed using Student’s *t* test and one-way ANOVA. *P < 0.05, **P < 0.01, ***P < 0.001, ****P < 0.0001.

Given our findings that TRIM21 promotes intracellular *Mtb* growth, we finally examined whether pharmacological inhibition of TRIM21 could restrict *Mtb* growth in experimental macrophage and murine models. To this end, we assessed the activity of fimepinostat, a drug recently shown to inhibit TRIM21 at both the mRNA and protein levels [29]. Fimepinostat treatment led to a marked reduction in TRIM21 protein levels in both murine BMDMs and human THP-1–derived macrophages (Fig 6A, S5 Fig A). Importantly, fimepinostat did not induce detectable cytotoxicity in macrophages (Fig 6B, S5 Fig B), but reduced intracellular *Mtb* growth in both cell types (Fig 6C, S5 Fig C).

Notably, compared with DMSO-treated macrophages, fimepinostat treatment exhibited significantly increased the co-localization of intracellular *Mtb* with SGs in macrophages (Fig 6D). We also observed that fimepinostat treatment significantly increased phosphorylated eIF2α levels in *Mtb*-infected cells (Fig 6E). Importantly, in TRIM21-deficient cells, the fimepinostat-induced reduction in intracellular bacterial burden was abolished, indicating that TRIM21 is required for the antibacterial effect of fimepinostat (Fig 6F).

To translate these findings in vivo, we tested fimepinostat in two independent animal infection models. In the zebrafish model, following intraperitoneal infection with *Mycobacterium marinum* for 9 days, treatment with fimepinostat improved host survival compared with controls (Fig 6G). Similarly, in mice infected with *Mtb* for 28 days, fimepinostat treatment resulted in reduced bacterial burdens in both the lungs and spleens, accompanied by decreased immune cell infiltration, relative to control-treated animals (Fig 6H–6L). Collectively, these data indicate that phenocopying TRIM21 loss with fimepinostat restricts intracellular *Mtb* growth in vitro and in vivo, supporting TRIM21 inhibition as a potential strategy for host-directed therapy against TB.

## Discussion

TB is a continuing global health burden, underscoring the need to better define host–pathogen interactions that shape disease susceptibility and immune regulation in order to inform the development of effective therapeutic strategies. In this study, we identify TRIM21 as a host factor that promotes intracellular *Mtb* growth and increases susceptibility to TB in mice through a mechanism distinct from of its well-characterized E3 ubiquitin ligase activity. Mechanistically, TRIM21 directly interacts with the RNA helicase DDX3X, thereby disrupting liquid–liquid phase separation dynamics required for SG formation. Importantly, phenocopying TRIM21 loss with fimepinostat enhances host resistance to *Mtb* infection in both cellular and animal models. Together, these findings uncover a previously unrecognized TRIM21-mediated mechanism by which *Mtb* evades innate immune defenses and position TRIM21 as a potential target for HDT in TB.

While prior studies have established a protective function for TRIM21 in antiviral immunity [5–7], accumulating evidence indicates that it can negatively regulate antibacterial immune responses [8]. Although we previously observed upregulation of TRIM21 expression during *Mtb* infection and demonstrated its role in promoting NCOA4-mediated ferritin degradation to support intracellular *Mtb* growth [9,10], the broader contribution of TRIM21 to *Mtb* pathogenesis has remained incompletely defined. Here, we show that TRIM21 promotes intracellular *Mtb* growth in both macrophages and mice through a mechanism distinct with its canonical E3 ligase activity. This conclusion is supported by the observation that TRIM21 depletion further reduced *Mtb* growth even in NCOA4-deficient macrophages, in which the ferritinophagy pathway is functionally disrupted. Here, we show that TRIM21 physically interacts with DDX3X but does not regulate its protein abundance via ubiquitin-mediated proteasomal degradation. Instead, our data identify the central coiled-coil domain of TRIM21, rather than the RING domain that confers E3 ligase activity, as the key structural element mediating DDX3X interaction and supporting intracellular *Mtb* growth.

SG formation is now recognized as a conserved host response to microbial infections. In bacterial infections, *Shigella flexneri* activates the GCN2–eIF2α signaling pathway, leading to SG assembly [30]. Similarly, several viral RNAs activate protein kinase R (PKR), resulting in eIF2α phosphorylation and subsequent SG formation [31,32]. In the context of mycobacterial infection, multiple species, including *Mycobacterium avium*, *Mycobacterium ulcerans*, and *Mtb*, share a conserved capacity to modulate host stress responses through eIF2α phosphorylation [18,33,34]. Beyond their role in translational control, SGs have emerged as important regulators of inflammatory signaling and infection pathogenesis. SGs can modulate inflammatory responses by sequestering key proteins and mRNAs involved in immune signaling pathways. For example, astrin has been shown to inhibit mTOR–raptor association and recruit raptor to SGs, thereby restricting mTORC1 activation under conditions of metabolic challenge and redox stress [35]. SGs can also sequester TNF receptor–associated factor 2, leading to suppression of pro-inflammatory TNF-α signaling [36]. In addition, SGs contribute to antiviral defense by capturing viral RNAs and innate immune sensors such as RIG-I, thereby restricting viral replication and promoting innate immune activation [37]. During *Mtb* infection, SGs form protective structures near damaged lysosomes, stabilizing ruptured membranes and facilitating their repair, which limits bacterial growth [18]. Notably, many viruses have evolved strategies to counteract this host defense mechanism by actively suppressing SG formation. For example, infection with SARS-CoV-2 or hepatitis C virus (HCV) leads to depletion of the SG core protein G3BP1 and inhibition of SG assembly [38,39], while other viruses interfere with SG formation by blocking PKR activation [40–42]. In this study, we demonstrate that *Mtb* similarly suppresses SG formation through TRIM21-mediated signaling, thereby creating a permissive intracellular environment that supports bacterial growth. Importantly, a previous study identified TRIM21 as a key regulator of SG homeostasis, showing that TRIM21 inhibits SG formation by catalyzing ubiquitination of the core SG protein G3BP1, whereas autophagy receptors such as SQSTM1 and CALCOCO2 mediate SGs clearance [43]. Consistent with previous observations that TRIM21 can limit SG accumulation, our findings similarly demonstrate that TRIM21 suppresses SG formation. However, our study extends this concept by identifying an infection-specific mechanism in which TRIM21 regulates SG assembly through its interaction with DDX3X. Nevertheless, we recognize that our study does not directly assess selective autophagic clearance of SGs or xenophagy of *Mtb*-containing compartments. Therefore, the interplay between TRIM21-mediated SG regulation and selective autophagy pathways, including xenophagy, remains an important and unresolved question that warrants further investigation.

There are also several limitations to consider when interpreting our results. First, given that TRIM21 is a well-characterized E3 ubiquitin ligase, we further examined whether TRIM21 regulates DDX3X through ubiquitination. In our experimental system, TRIM21 manipulation did not alter DDX3X protein abundance, and proteasome inhibition by MG132 did not restore DDX3X-associated activity, arguing against a proteasome-dependent degradation mechanism. However, we cannot formally exclude the possibility that TRIM21 may catalyze non-degradative ubiquitination or low-level ubiquitination of DDX3X under specific cellular contexts.

Second, some mechanistic analyses were performed under transient overexpression conditions in 293T cells. While this system is advantageous for dissecting protein interaction interfaces, it is important to acknowledge that elevated protein expression may promote non-physiological condensate formation. Therefore, the effects of protein overexpression on SG formation and dynamics should be considered when interpreting these findings.

Third, although phenocopying TRIM21 loss with fimepinostat reduced *Mtb* growth, this effect resulted from global TRIM21 protein inhibition rather than selective modulation of its function, such as its E3 ligase activity or DDX3X binding. Treatment with fimepinostat, a dual PI3K/HDAC inhibitor previously reported to reduce TRIM21 expression, resulted in decreased bacterial burden in *Mtb*-infected mice. Given the broad transcriptional and metabolic effects of PI3K/HDAC inhibition, these findings should not be interpreted as evidence of specific TRIM21 inhibition. Moreover, given that TRIM21 is expressed in multiple immune cell types, including T cells, B cells, and dendritic cells [44], future research should investigate its cell type–specific functions during *Mtb* infection. It will also be important to resolve the relative contributions of TRIM21’s enzymatic versus scaffold functions across different stages of infection.

Finally, although Trim21-deficient mice exhibited enhanced resistance to *Mtb*, we used a global knockout model rather than a myeloid-specific deletion strategy. As a result, the observed phenotypes may reflect contributions from immune cell populations beyond macrophages.

In summary, our work identifies TRIM21 as a host factor that promotes *Mtb* growth through a non-canonical mechanism distinct with its E3 ubiquitin ligase activity. We further define a previously unappreciated pathway in TB pathogenesis in which TRIM21 suppresses stress granule formation and compromises innate immune defenses. Supported by both genetic and pharmacological evidence, these findings highlight TRIM21 as a promising candidate target for host-directed therapeutic strategies against TB.

## Materials and methods

### Bacterial strains and culture conditions

*Mtb* strains (H37Ra, H37Rv, GFP-H37Rv) were cultured in 7H9 broth (BD Biosciences, San Jose, CA, USA) containing 0.2% glycerol (Sigma-Aldrich, Merck, Darmstadt, Germany), 10% Middlebrook oleic acid-albumin-dextrose-catalase enrichment medium (BD Biosciences), and 0.05% Tween 80 (Sigma-Aldrich) for 10–14 days at 37°C to reach the logarithmic growth phase (OD600 = 0.3-0.8). The concentration of bacteria was determined by the OD 600 as a function of CFU per milliliter.

### Cell culture and mice

The human monocytic cell line THP-1 and HEK293T cells were obtained from the Cell Bank of the Chinese Academy of Sciences (Shanghai, China). THP-1 cells were cultured in RPMI 1640 medium (Corning, USA) supplemented with 10% fetal bovine serum (Gibco, USA) at 37°C in a humidified atmosphere containing 5% CO_2_. THP-1 cells (5 × 10^5^ cells/ml) were seeded into 12-well plates in complete RPMI 1640 medium and differentiated using phorbol 12-myristate 13-acetate (PMA; Sigma, #P8139). The cells were then incubated in fresh complete RPMI 1640 medium for 12 h at 37°C before subsequent experiments.

HEK293T cells were cultured in DMEM (Corning) supplemented with 10% fetal bovine serum and 1% penicillin/streptomycin (C0222; Beyotime) at 37°C in a humidified atmosphere containing 5% CO_2_. For cell stress experiments, HEK293T cells were treated with NaAsO_2_ (0.5 mM) for 1 h, followed by confocal microscopy analysis.

Mouse peritoneal macrophages were isolated from C57BL/6 mice and cultured in DMEM (Corning) supplemented with 1 mM sodium pyruvate (Sigma), 2 mM L-glutamine (Sigma), and 10% fetal bovine serum. Bone marrow–derived macrophages (BMDMs) were isolated from C57BL/6 mice and cultured in DMEM supplemented with 20% L929 cell–conditioned medium, 1 mM sodium pyruvate, 2 mM L-glutamine, and 10% fetal bovine serum for 7 days. Trim21^−/−^ mice were obtained from Nanmo Biotechnology (Guangzhou, China). All animal experiments were conducted in accordance with guidelines approved by the Institutional Animal Committee of Shenzhen University School of Medicine.

### CFU assay

BMDMs and PMA-differentiated THP-1 macrophages (5 × 10^5^ cells/ml) were infected with *Mtb* strain H37Rv at a multiplicity of infection (MOI) of 3 for 6 h at 37°C with 5% CO_2_. The medium was then replaced with fresh complete medium, and infected macrophages were cultured for an additional 72 h. At 6 h and 72 h post-infection, cells were washed three times with PBS and lysed in 500 μl sterile PBS containing 0.1% SDS for 10 min at room temperature. Serial dilutions were plated on Middlebrook 7H10 agar (BD Biosciences) plates and incubated at 37°C for 15–20 days before colony enumeration.

### Plasmids, siRNAs, and transfection

For protein expression in mammalian cells, TRIM21 (HA-tagged or EGFP-tagged), TRIM21 truncation mutants (HA-tagged), and DDX3X (Flag-tagged or mCherry-tagged) were cloned into pcDNA3.1 vectors. HEK293T cells were transiently transfected with the indicated plasmids using jetPRIME transfection reagent (PolyPlus-transfection, France), and total RNA and protein were extracted 48 h after transfection. *Trim21* mutants ligated into the pLenti-CMV-HA plasmid were generated by gene synthesis (Tongyong Biotech, Anhui, China) and transfected into BMDMs in the presence of 8 μg/ml polybrene.

For siRNA-mediated knockdown, TRIM21 siRNA (5′-GTGAAGCAGCCTCCTTATA-3′) was transfected into PMA-differentiated THP-1 macrophages using Lipofectamine RNAiMAX (Invitrogen). siRNA–liposome complexes were prepared in Opti-MEM (Gibco). After 6 h, the medium was replaced, and cells were cultured for an additional 36–48 h. Total RNA and protein were then extracted, and knockdown efficiency was assessed by RT–qPCR or western blotting.

To generate lentivirus for TRIM21 knockout, the LentiCRISPRv2 plasmid containing a TRIM21-specific single-guide RNA (sgRNA; ATGCTCACAGGCTCCACGAA) was transfected into HEK293T cells in the presence of 8 μg/ml polybrene.

### Co-immunoprecipitation (Co-IP) and mass spectrometry

Co-IP experiments were conducted as previously described [45]. In brief, cells were lysed in IP buffer (P0013; Beyotime) supplemented with a protease inhibitor cocktail. The extracted proteins were incubated overnight at 4°C with anti-TRIM21 antibody (1:200 dilution in TBS) Protein–antibody complexes were then captured using protein A/G immunoprecipitation magnetic beads (P2108; Beyotime) for an additional 4 h. The precipitated complexes were washed five times with IP buffer to remove non-specific binding and eluted with 1 × SDS loading buffer. Eluted samples were heated at 95°C for 5–10 min to denature proteins and dissociate them from the beads, followed by immunoblotting using anti-DDX3X antibody (1:1000 dilution in TBS).

### Mass spectrometry analysis

THP-1 cell lysates from both *Mtb*-infected and uninfected groups were first pre-cleared with IgG magnetic beads to reduce non-specific binding. TRIM21 protein levels in whole-cell lysates were assessed by immunoblotting. Based on these measurements, the amount of lysate used for each condition was adjusted to ensure comparable TRIM21 abundance between groups. Subsequently, immunoprecipitation was performed using equal amounts of anti-TRIM21 antibody for both samples. For mass spectrometry, the precipitates immunoprecipitated with anti- TRIM21 antibody were eluted using an elution buffer (0.5 mol/L NH4OH, 0.5 mmol/L EDTA), according to the manufacturer’s instructions. Samples were concentrated using an evaporator and subjected to liquid chromatography–mass spectrometry analyses. The proteomics identification results are provided in S1 Table. Protein identification confidence was assessed based on the −10lgP score. The number of peptides and unique peptides was used as additional criteria to evaluate protein identification reliability. Sequence coverage (%) was calculated to reflect the proportion of the protein sequence represented by identified peptides. Signal intensity/abundance of the protein in the sample, derived from MS peak area.

### Mouse infection model

Our study examined male and female animals, and similar findings are reported for both sexes. Six- to eight-week-old Trim21^−/−^ and Trim21^+/+^ mice were randomly assigned to infected or uninfected control groups. Mice in the infected groups were exposed to approximately 100–200 CFU of the *Mtb* H37Rv strain using a Glas-Col inhalation exposure system. At 28 days post-infection, mice were euthanized and lung tissues were harvested and homogenized. Ten-fold serial dilutions of the homogenates were plated on Middlebrook 7H10 agar to determine bacterial burden in the lungs. Colony-forming units (CFUs) were enumerated after 2–3 weeks of incubation at 37°C. Lung tissues were also processed for histopathological analysis by hematoxylin and eosin staining. All animal experiments were conducted in accordance with guidelines approved by the Institutional Animal Committee of Shenzhen University School of Medicine.

For pharmacological studies, six- to eight-week-old C57BL/6 mice were infected with approximately 100–200 CFU of the *Mtb* H37Rv strain using the Glas-Col inhalation exposure system. After 28 days of infection, mice were treated every other day by oral gavage with fimepinostat (CUDC-907; Selleck, S2759) at 35 mg/kg in 200 μL of 0.9% saline. Mice were euthanized after 14 days of treatment, and lung tissues were harvested, homogenized, and processed for CFU enumeration as described above.

### Zebrafish and infection

Adult zebrafish were intraperitoneally infected with *Mycobacterium marinum* (500 CFU per fish). Infections were performed under anesthesia with 0.02% tricaine methanesulfonate (MS-222). Infected zebrafish were randomly assigned to treatment groups receiving fimepinostat (25 nM or 50 nM) or vehicle control (DMSO). Treatments were administered daily via water bath exposure for 9 days.

### SG fractionation and enrichment

SG fractionation and enrichment were performed as previously described [43,46]. PMA-differentiated THP-1 macrophages (5 × 10^5^ cells/ml) were infected with *Mtb* strain H37Ra at a multiplicity of infection (MOI) of 5 for 48 h at 37°C with 5% CO_2_. Cells were treated with 0.029% digitonin (Beyotime, China; Y263961) for 30 s to permeabilize the plasma membrane and then washed once with PBS. Cells were suspended in 200 µl stress granule lysis buffer (50 mM Tris–HCl, pH 7.6; 50 mM NaCl; 100 mM potassium acetate; 5 mM MgCl_2_; 0.5 mM DTT; 50 μg/ml heparin; 0.5% NP40; 1:5000 Antifoam B; 1% EDTA-free protease inhibitor cocktail; and RNase inhibitor in DEPC-treated H2O). After syringe lysis, stress granules were enriched by density gradient centrifugation at 4°C (1,000 × g for 5 min, followed by 18,000 × g for 20 min). Pellets were resuspended in 90 μl stress granule lysis buffer and centrifuged at 850 × g for 2 min at 4°C. The resulting supernatants (S850) represent the stress granule core–enriched fractions.

### Immunofluorescence, and confocal microscope imaging

For immunofluorescence microscopy, cells were grown on confocal dishes. Following transfection, drug treatment, or H37Rv infection, cells were washed twice with PBS, fixed with 4% paraformaldehyde for 15 min, permeabilized with 0.3% Triton X-100 for 10 min, and blocked with 3% bovine serum albumin (BSA) for 2 h at room temperature. Cells were then incubated with primary antibodies overnight at 4°C, followed by incubation with secondary antibodies for 2 h at room temperature. After three washes with PBST, nuclei were counterstained with DAPI (Beyotime, China; C1002) for 10 min. Fluorescence images were acquired using a Zeiss confocal microscope (LSM 880, Zeiss Microscopy, Germany) equipped with a 63 × oil-immersion objective.

### Antibodies

The following antibodies were used for western blotting or immunoprecipitation: rabbit recombinant monoclonal TRIM21/SS-A antibody (ab207728; Abcam), TRIM21 monoclonal antibody (67136–1-lg; Proteintech), DDX3X rabbit antibody (A5637; ABclonal), G3BP1 polyclonal antibody (13057–2-AP; Proteintech), ABflo 555-conjugated goat anti-rabbit IgG (H + L) (AS058; ABclonal), ABflo 555-conjugated goat anti-mouse IgG (H + L) (AS057; ABclonal), ABflo 488-conjugated goat anti-mouse IgG (H + L) (AS037; ABclonal), ABflo 488-conjugated goat anti-rabbit IgG (H + L) (AS053; ABclonal), HRP-conjugated goat anti-rabbit IgG (H + L) (AS014; ABclonal), HRP-conjugated goat anti-mouse IgG (H + L) (AS003; ABclonal), GAPDH monoclonal antibody (60004–1-Ig; Proteintech), β-actin rabbit monoclonal antibody (AC038; ABclonal), DYKDDDDK tag polyclonal antibody (binds to FLAG tag epitope) (20543–1-AP; Proteintech), HA tag polyclonal antibody (51064–2-AP; Proteintech), anti-phospho-eIF2α (Ser51) (9721; Cell Signaling Technology), and phospho-STAT1-Y701 Rabbit mAb (AP0054; ABclonal).

### RNA isolation and quantitative real-time PCR

Total RNA was extracted using an RNA isolation kit (RC102–01; Vazyme) and stored at −80°C until use. Reverse transcription was performed using reverse transcriptase (R433-01; Vazyme). Quantitative PCR was carried out using a SYBR Green qRT-PCR kit (Q221-01; Vazyme) on an Applied Biosystems StepOnePlus Real-Time PCR System. Relative gene expression was calculated using the 2-ΔΔCq method and normalized to glyceraldehyde 3-phosphate dehydrogenase (*GAPDH*). Primers were obtained from PrimerBank and were as follows: *GAPDH* (forward: 5′-GTCTCCTCTGACTTCAACAGCG-3′; reverse: 5′-ACCACCCTGTTGCTGTAGCCAA-3′), *TRIM21* (forward: 5′-TCAGCAGCACGCTTGACAAT-3′; reverse: 5′-GGCCACACTCGATGCTCAC-3′), *G3BP1* (forward: 5′-AGCCTGTTCAGAAAGTCCTTAGC-3′; reverse: 5′-CGAAGGCGATTATCTCGTCGGT-3′), *mTrim21* (forward: 5′-GGAGGATTCGTGGTTCAGAGCT-3′; reverse: 5′-GGCATGTGCTTGTTAGGTCTGG-3′) *m*β-*actin* (forward: 5′-CATTGCTGACAGGATGCAGAAGG-3′; reverse: 5′-TGCTGGAAGGTGGACAGTGAGG-3′).

### Lactate dehydrogenase assay

Lactate dehydrogenase (LDH) activity was measured using an LDH release assay kit (Beyotime, C0016) according to the manufacturer’s instructions. Absorbance was measured at 490 nm using a Varioskan LUX multimode microplate reader (Thermo Fisher Scientific).

### Bioinformatic analyses

Intrinsically disordered region (IDR) analysis was performed using the PONDR-VSL2 predictor (http://www.pondr.com). PONDR (Predictor of Natural Disordered Regions) analysis is a bioinformatics tool that predicts intrinsically disordered regions (IDRs) in protein sequences based on their amino acid composition and sequence features. It assigns a disorder score to each residue, and regions with scores above 0.5 are generally considered to have a high likelihood of being intrinsically disordered.

### Fluorescence recovery after photobleaching (FRAP) assay

FRAP assay was performed using the FRAP module of a Zeiss confocal microscopy system. Cells expressing TRIM21–EGFP or DDX3X–mCherry were cultured in glass-bottom dishes and imaged using a 63 × oil-immersion objective. Appropriate laser wavelengths were selected according to the fluorophore (488 nm for EGFP and 561 nm for mCherry), with low laser power for image acquisition and high laser power for photobleaching. Photobleaching was performed on a defined circular region of interest. Time-lapse images were acquired following bleaching. Fluorescence intensity within the region of interest was normalized to pre-bleach values, and background fluorescence was subtracted.

### Statistical analyses

All data are presented as means ± SD. Statistical analyses were performed using GraphPad Prism. Group comparisons were conducted using Student’s t test, and a P value < 0.05 was considered statistically significant.

### Study approval

This study was approved by the Ethics Committees of Shenzhen University and Guangzhou Eighth People’s Hospital (approval number: IACUC-DYC-2026-04). Written informed consent was obtained from all participants.

## Supporting information

S1 Fig TRIM21 deficiency further enhances clearance of *Mtb* from macrophages. (A-B) TRIM21 expression in peripheral blood from patients with pulmonary tuberculosis (TB) and healthy controls, based on the GSE107991 and GSE94438 datasets. (C) TRIM21 expression in HIV/TB co-infected individuals and HIV/latent tuberculosis infection cohorts from the GSE69581 dataset. (D) TRIM21 expression before and after anti-TB therapy at three time points, based on the GSE19491 dataset. (E) Quantitative real-time PCR (RT–qPCR) analysis of TRIM21 knockdown efficiency in macrophages. (F) Immunoblot analysis confirming TRIM21 knockdown efficiency in macrophages. (G) Immunoblot analysis of TRIM21 knockout efficiency in mouse tissues. (H) PCR genotyping of TRIM21 knockout mice; C193–C214 denote Trim21^-/-^ mice, and WT denotes Trim21^+/+^ mice. Statistical significance was assessed using Student’s t test and one-way ANOVA. ***P < 0.001, ****P < 0.0001.
(TIF)

S2 Fig TRIM21 deficiency reduces endomembrane damage and intracellular *Mtb* burden during infection. (A) Representative confocal microscopy images of *Mtb*-infected peritoneal macrophages at 24 h and 48 h, showing co-staining of *Mtb*, stress granule marker G3BP1, and the lysosomal damage marker Galectin-3 (scale bars: 10 μm). (B) Quantification of *Mtb* (bacteria area per cell) in Trim21^+/+^ and Trim21^-/-^ peritoneal macrophages at 24 and 48 h after infection. (C) Colocalization was analyzed with the ImageJ software. (D) Quantification of Gal-3 (per cell) in Trim21^+/+^ and Trim21^-/-^ peritoneal macrophages at 24 and 48 h after infection. Statistical significance was assessed using Student’s t test. *P < 0.05, **P < 0.01, ****P < 0.0001.
(TIF)

S3 Fig TRIM21 does not regulate DDX3X stability via ubiquitin-mediated proteasomal degradation. (A–C) Immunoblot analysis of DDX3X and TRIM21 expression in TRIM21-knockdown THP-1–derived macrophages after *Mtb* infection at the indicated time points. (D) Immunoblot analysis of DDX3X and TRIM21 in THP-1–derived macrophages infected with H37Rv for the indicated durations in the presence or absence of the proteasome inhibitor MG132 (10 mM) and/or cycloheximide (CHX; 10 mM), both of which were added during infection.
(TIF)

S4 Fig TRIM21 lacking the coiled-coil domain fails to inhibit SG formation. (A) Immunofluorescence analysis of G3BP1 in HEK293T cells transfected with TRIM21 or TRIM21-△CC plasmids and treated with NaAsO_2_ (0.5 mM, 1 h). (scale bars: 5 μm) (B) Intracellular H37Rv CFUs in TRIM21-knockdown and NCOA4-knockout THP-1–derived macrophages. Statistical significance was assessed using one-way ANOVA. ***P < 0.001, ****P < 0.0001.
(TIF)

S5 Fig Fimepinostat reduces TRIM21 protein levels and is associated with reduced intracellular *Mtb* burden. (A) Immunoblot analysis of TRIM21 expression in fimepinostat-treated THP-1–derived macrophages following *Mtb* infection. (B) Lactate dehydrogenase assay assessing cytotoxicity of fimepinostat in THP-1–derived macrophages under uninfected and *Mtb*-infected conditions. (C) Intracellular H37Rv CFUs in THP-1–derived macrophages treated with the indicated concentrations of fimepinostat. Data are presented as means ± SD. P < 0.05, ****P < 0.0001 (Student’s t test).
(TIF)

S1 Video FRAP assay of TRIM21-EGFP droplets in 293T cells. (MP4)

S2 Video FRAP assay of DDX3X-mCherry droplets in 293T cells. (MP4)

S3 Video FRAP assay of DDX3X-mCherry droplets in 293T cells transfected with the TRIM21 plasmid. (MP4)

S1 File Raw gel images. (PDF)

S1 Table Mass spectrometric identification of TRIM21-interacting proteins in THP-1 macrophages with or without *Mtb* infection. (XLSX)
