# Supplementary material for: TRIM21 blocks DDX3X-driven stress granule formation during Mycobacterium tuberculosis infection via a non-canonical E3 ligase mechanism
Source: PLoS Pathog. 2026 Sep 8;22(9):e1014553. doi: 10.1371/journal.ppat.1014553 (PMC13552953; doi:10.1371/journal.ppat.1014553)
Supplement: Supplementary file 1 [file ppat.1014553.s009.pdf]

Figure 2D

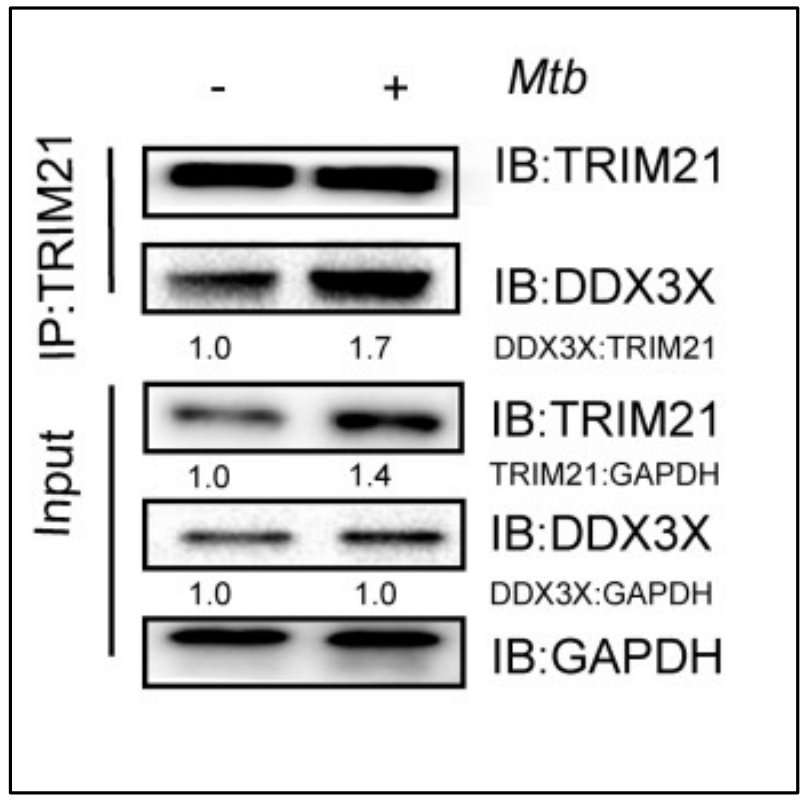

- + Mtb
IP:TRIM21
IB:TRIM21
IB:DDX3X
1.0 1.7 DDX3X:TRIM21
Input
IB:TRIM21
1.0 1.4 TRIM21:GAPDH
IB:DDX3X
1.0 1.0 DDX3X:GAPDH
IB:GAPDH

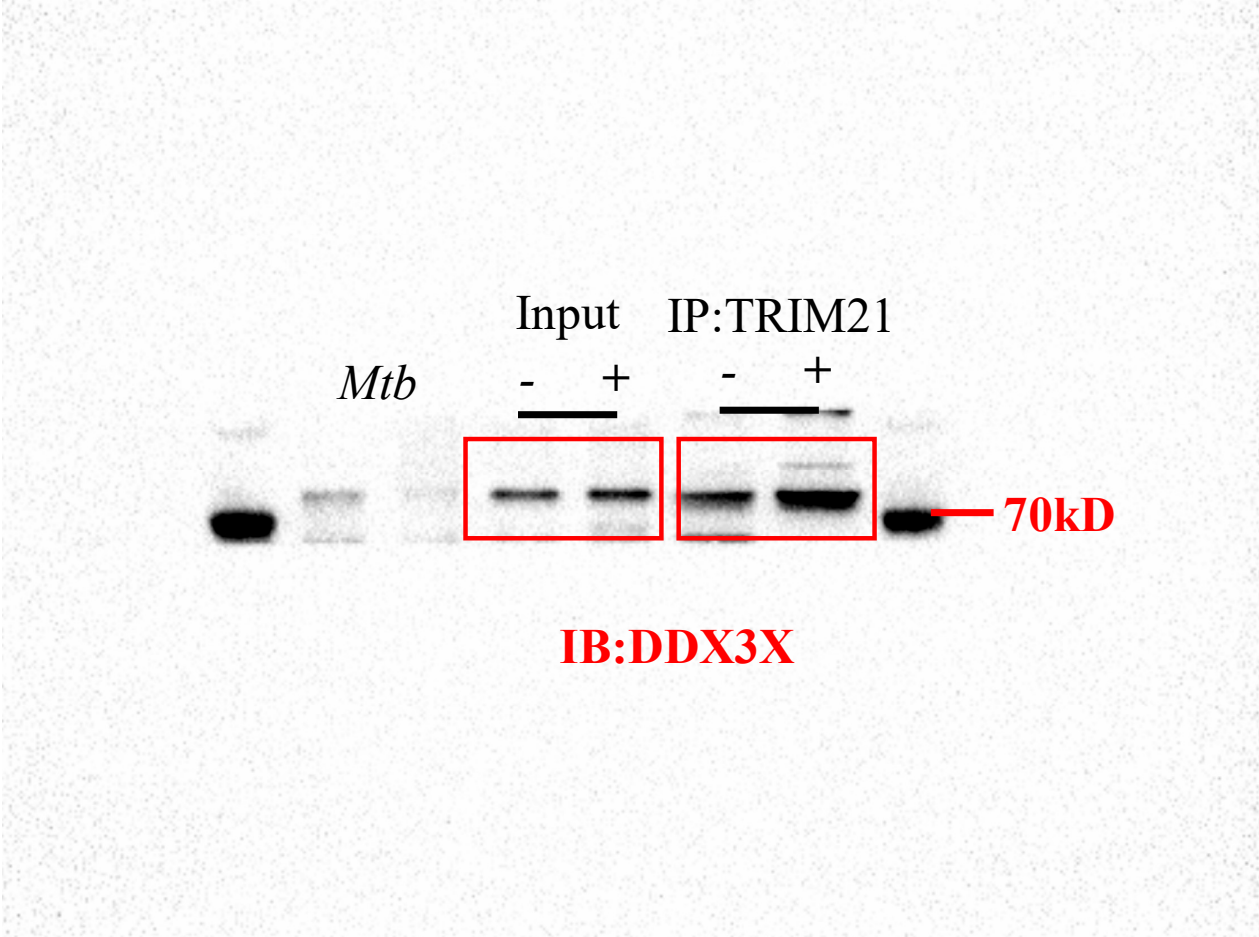

Input IP:TRIM21
Mtb - + - +
70kD
IB:DDX3X

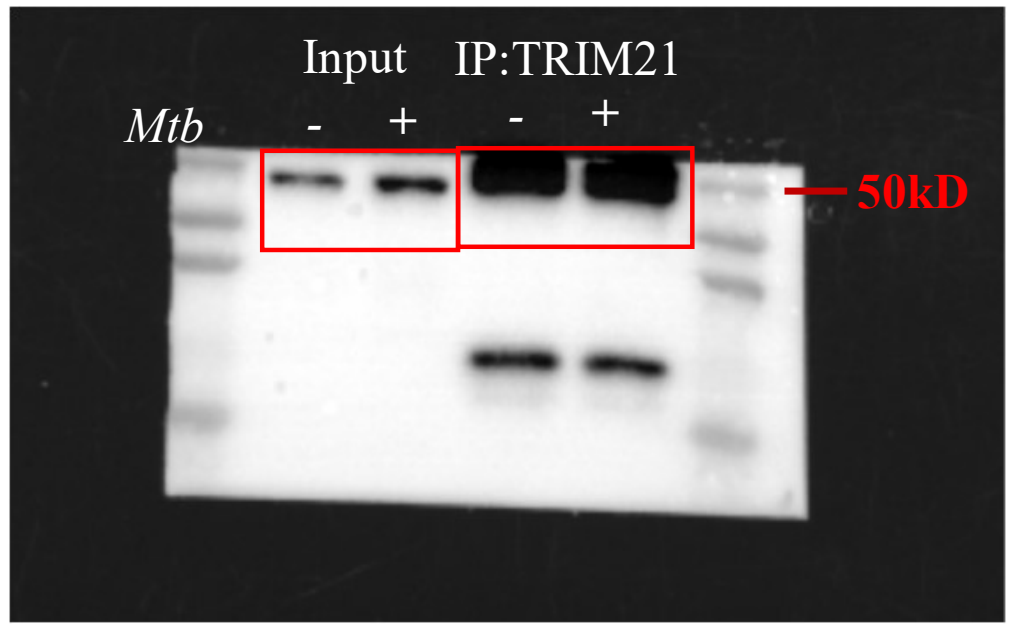

Input IP:TRIM21
Mtb - + - +
50kD

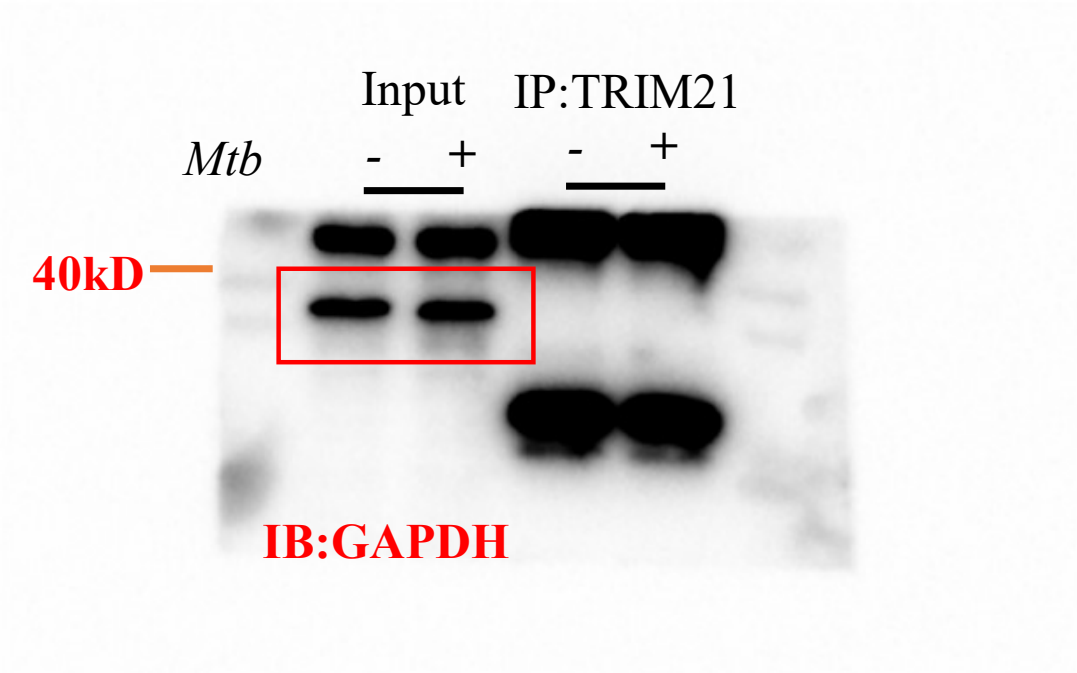

Input IP:TRIM21
Mtb - + - +
40kD
IB:GAPDH

Figure 3F

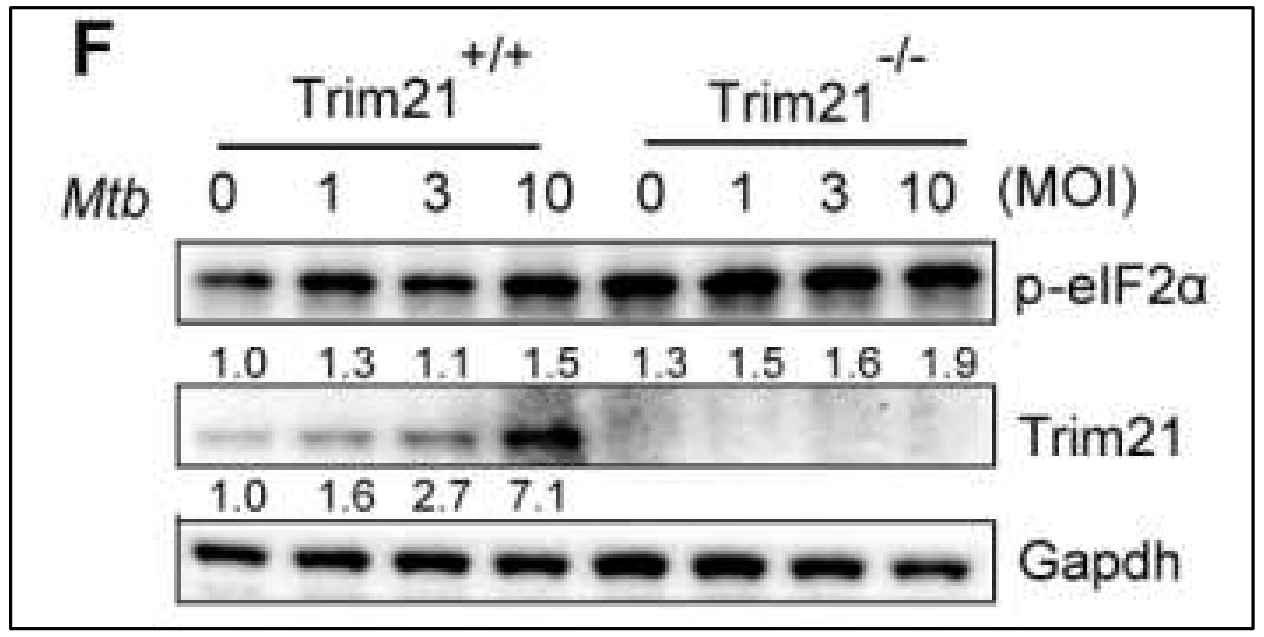
F
Trim21 +/+
Trim21 -/-
Mtb 0 1 3 10 0 1 3 10 (MOI)
p-eIF2α
1.0 1.3 1.1 1.5 1.3 1.5 1.6 1.9
Trim21
1.0 1.6 2.7 7.1
Gapdh

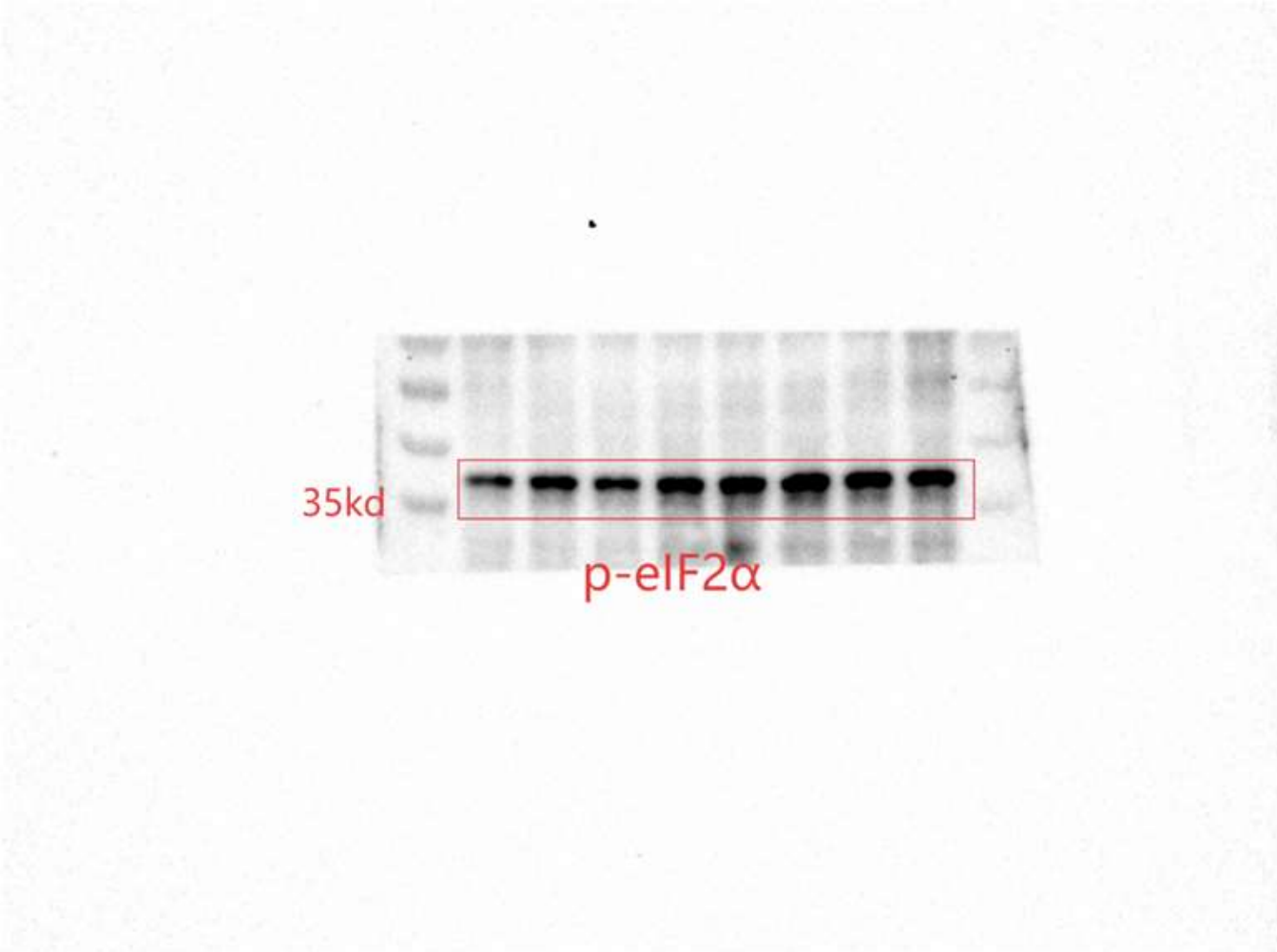
35kd
p-eIF2α

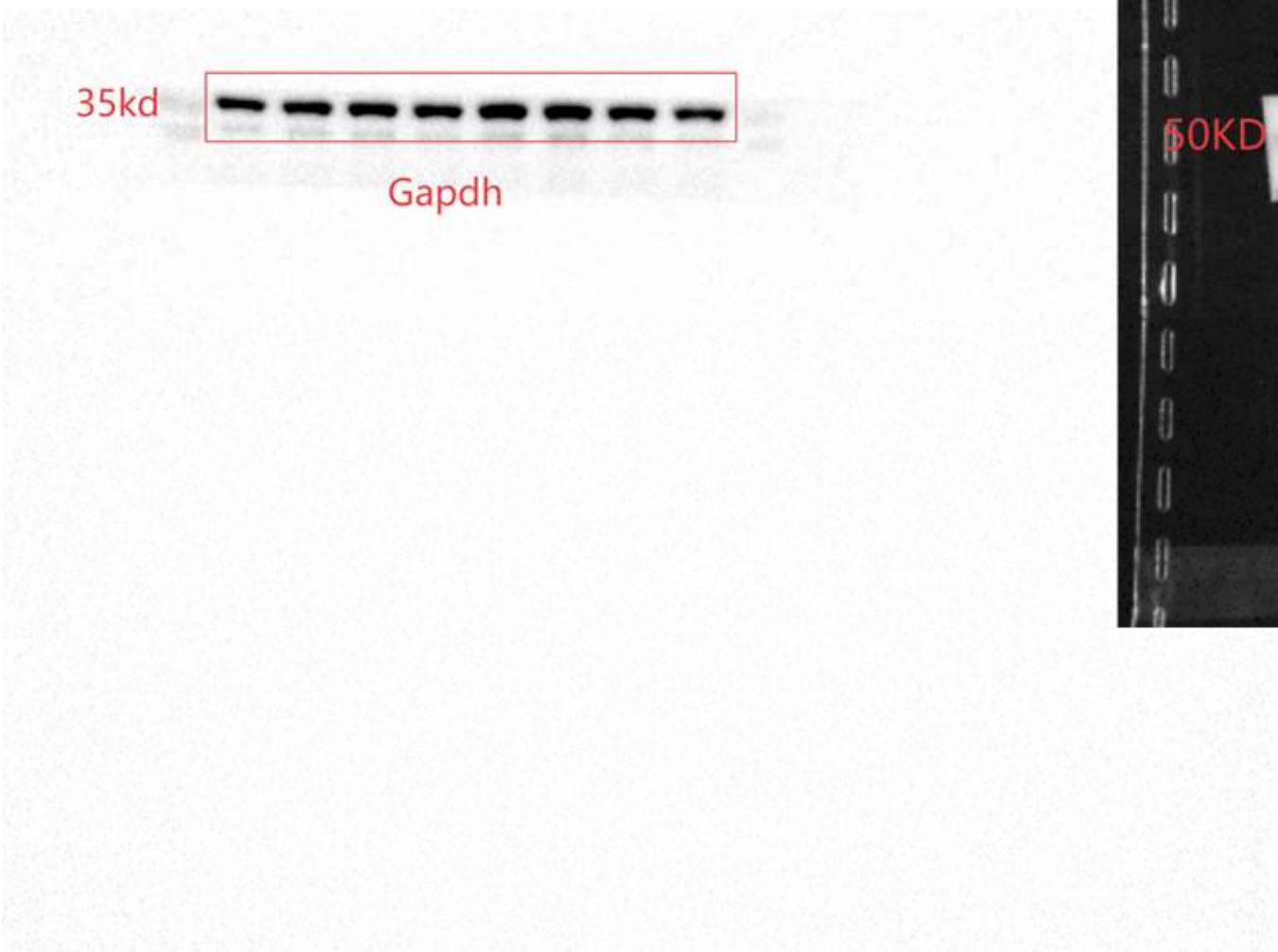
35kd
Gapdh

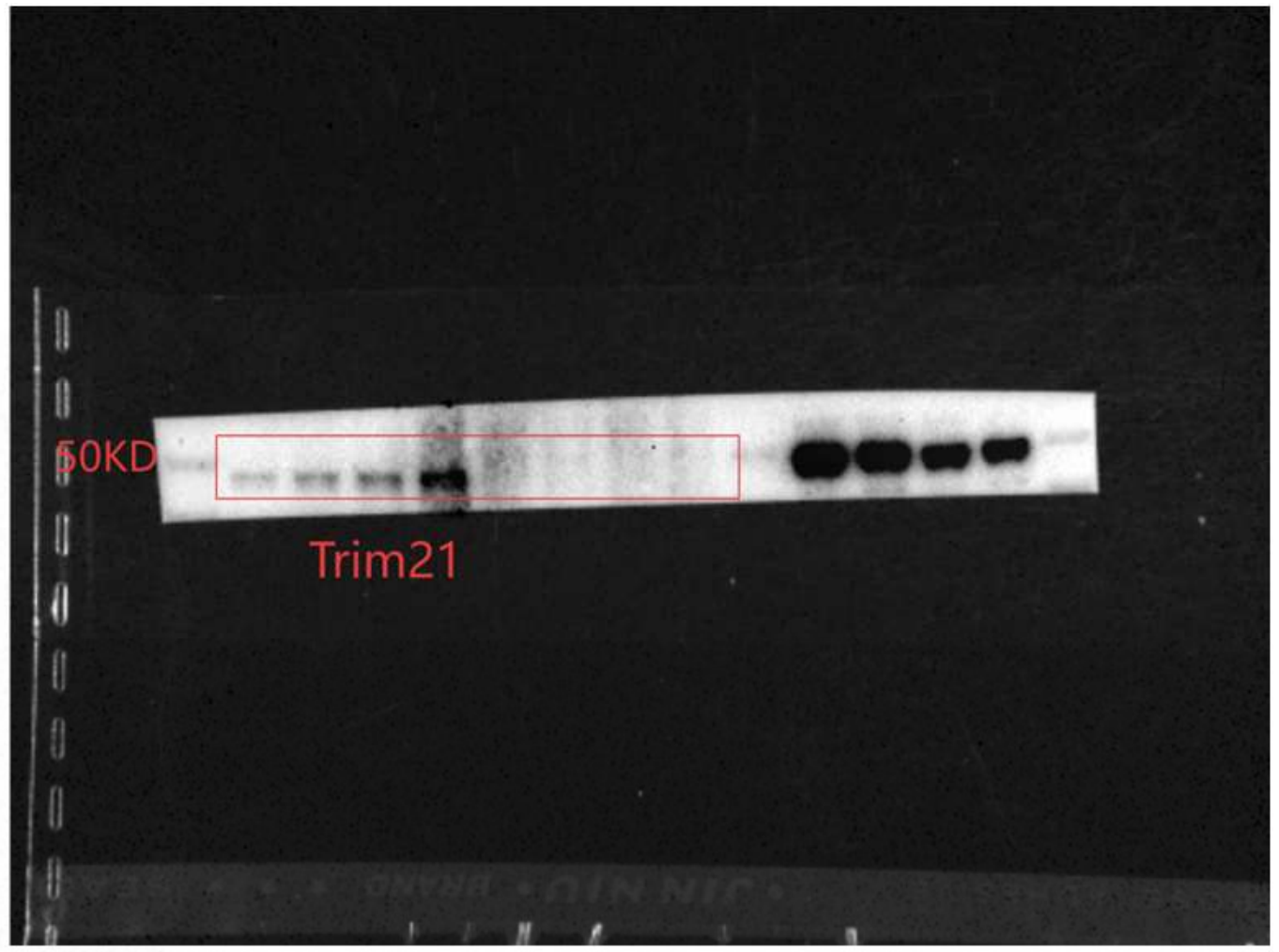
50KD
Trim21

Figure 3G

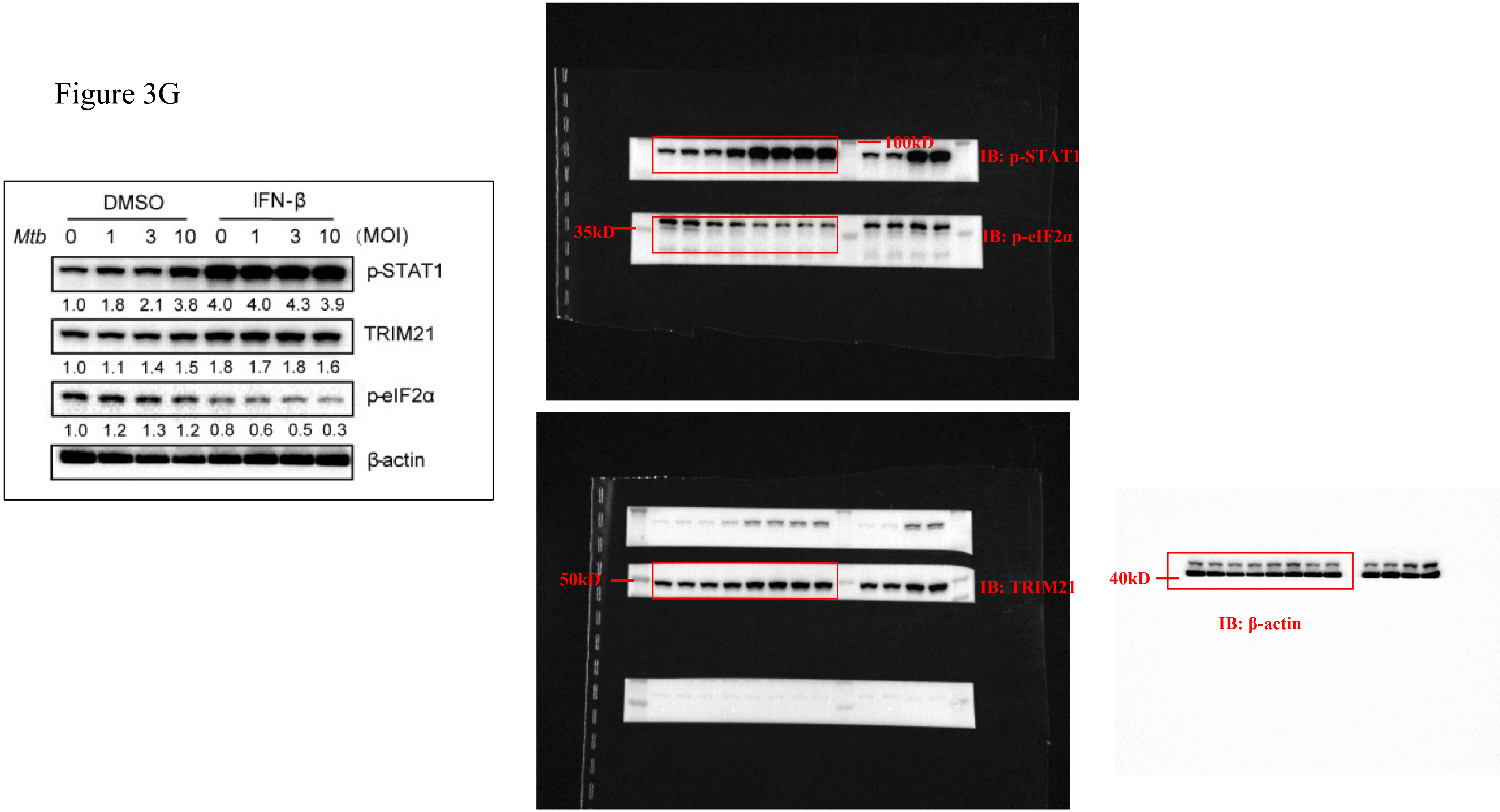
DMSO
IFN-β
Mtb 0 1 3 10 0 1 3 10 (MOI)
p-STAT1
1.0 1.8 2.1 3.8 4.0 4.0 4.3 3.9
TRIM21
1.0 1.1 1.4 1.5 1.8 1.7 1.8 1.6
p-eIF2α
1.0 1.2 1.3 1.2 0.8 0.6 0.5 0.3
β-actin
100kD
IB: p-STAT1
35kD
IB: p-eIF2α
50kD
IB: TRIM21
40kD
IB: β-actin

Figure 3J

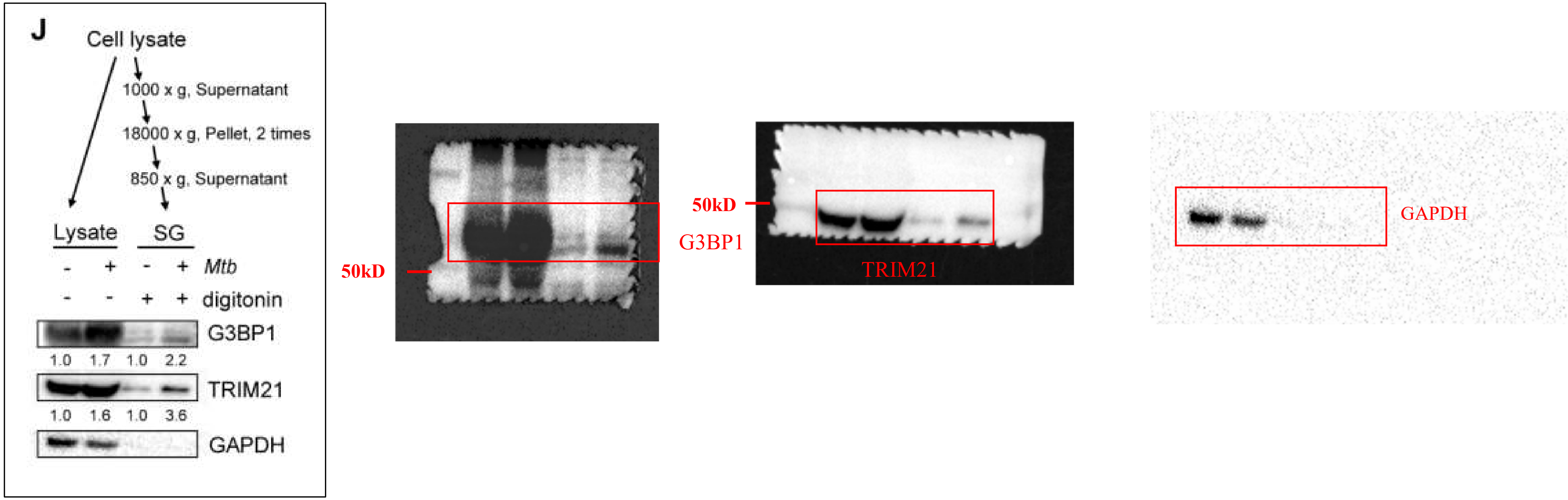
J
Cell lysate
1000 x g, Supernatant
18000 x g, Pellet, 2 times
850 x g, Supernatant
Lysate
SG
- + - + Mtb
- - + + digitonin
G3BP1
1.0 1.7 1.0 2.2
TRIM21
1.0 1.6 1.0 3.6
GAPDH
50kD
G3BP1
50kD
TRIM21
GAPDH

Figure 4I

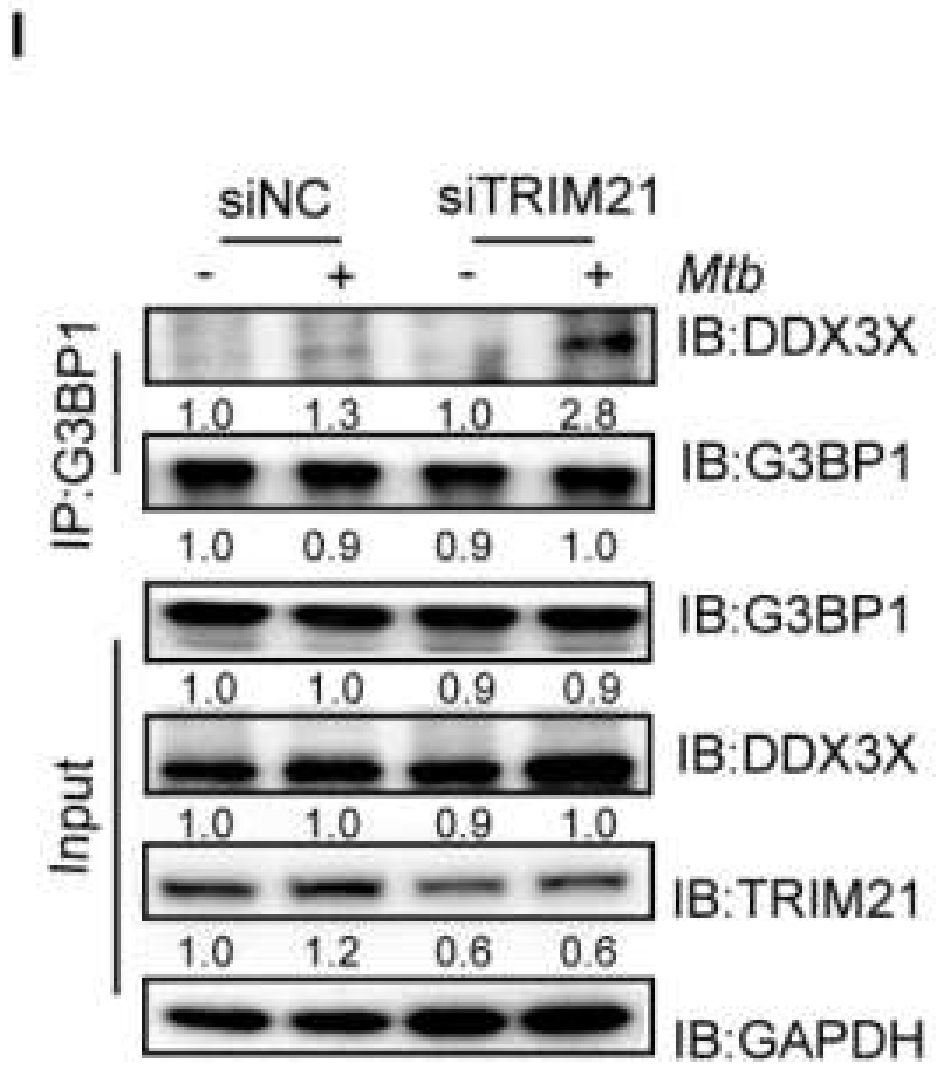
I
siNC
siTRIM21
- + - +
Mtb
IP:G3BP1
IB:DDX3X
1.0 1.3 1.0 2.8
IB:G3BP1
1.0 0.9 0.9 1.0
IB:G3BP1
1.0 1.0 0.9 0.9
IB:DDX3X
1.0 1.0 0.9 1.0
IB:TRIM21
1.0 1.2 0.6 0.6
IB:GAPDH
Input

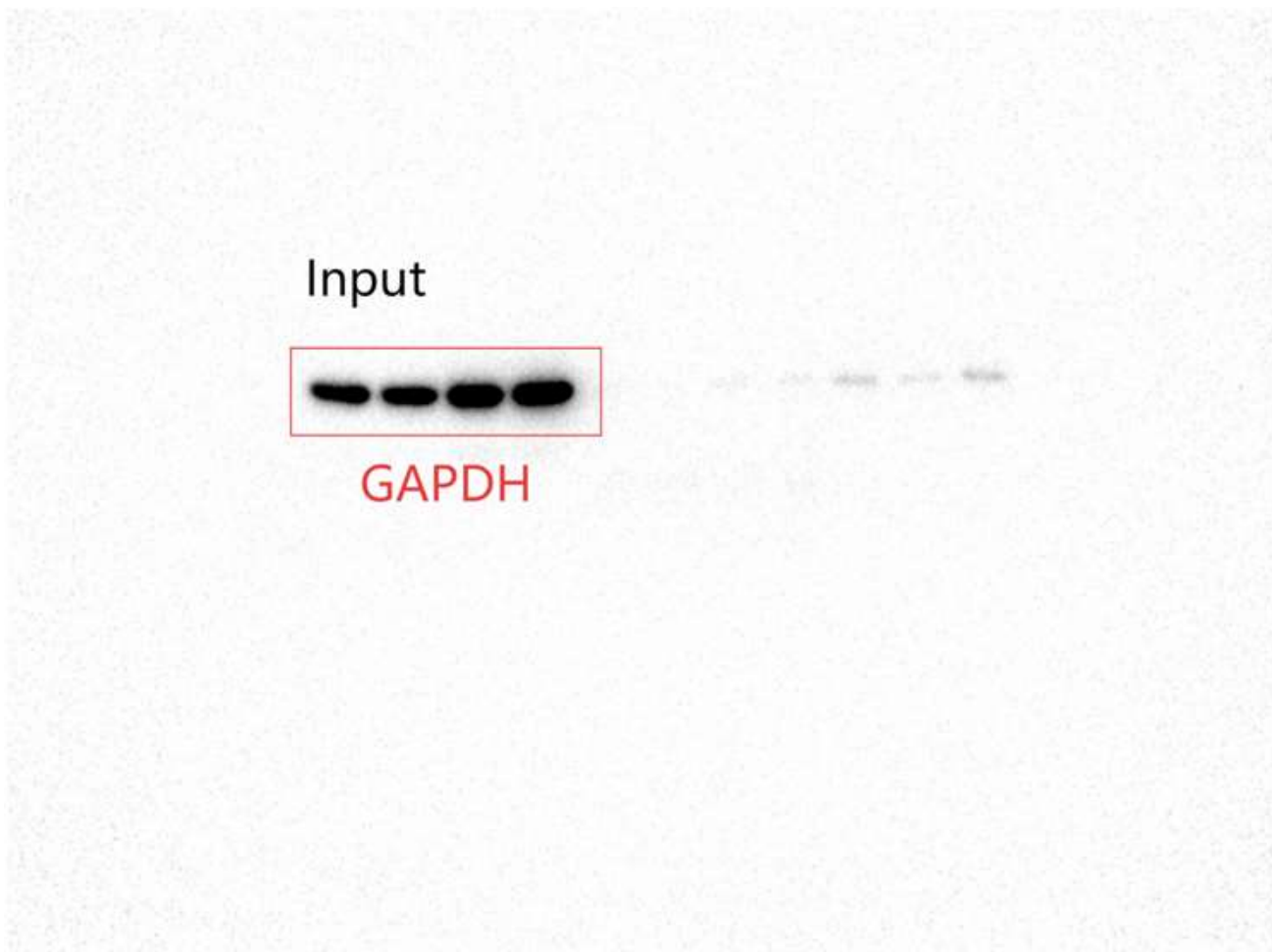
Input
GAPDH

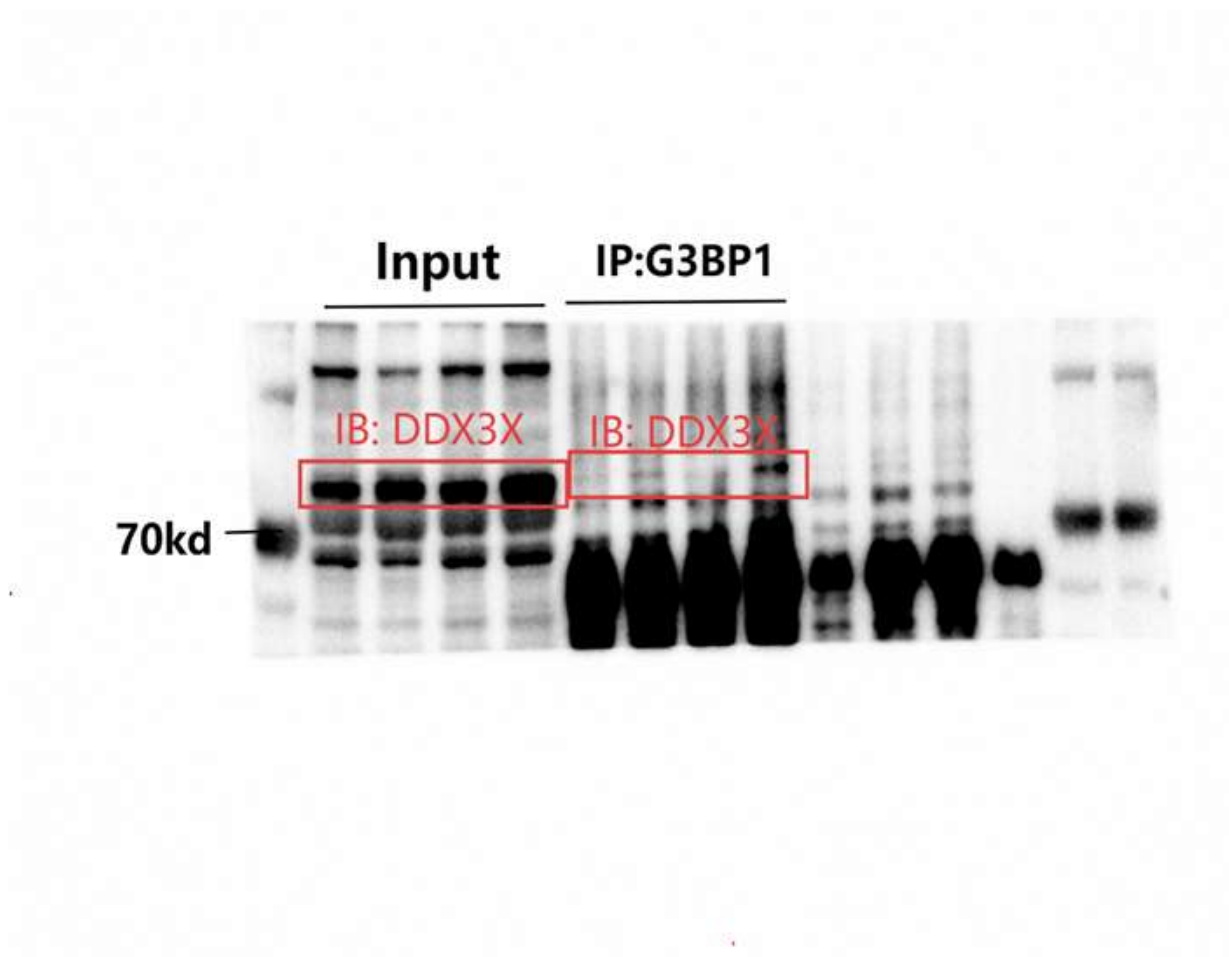
Input
IP:G3BP1
IB: DDX3X
IB: DDX3X
70kd

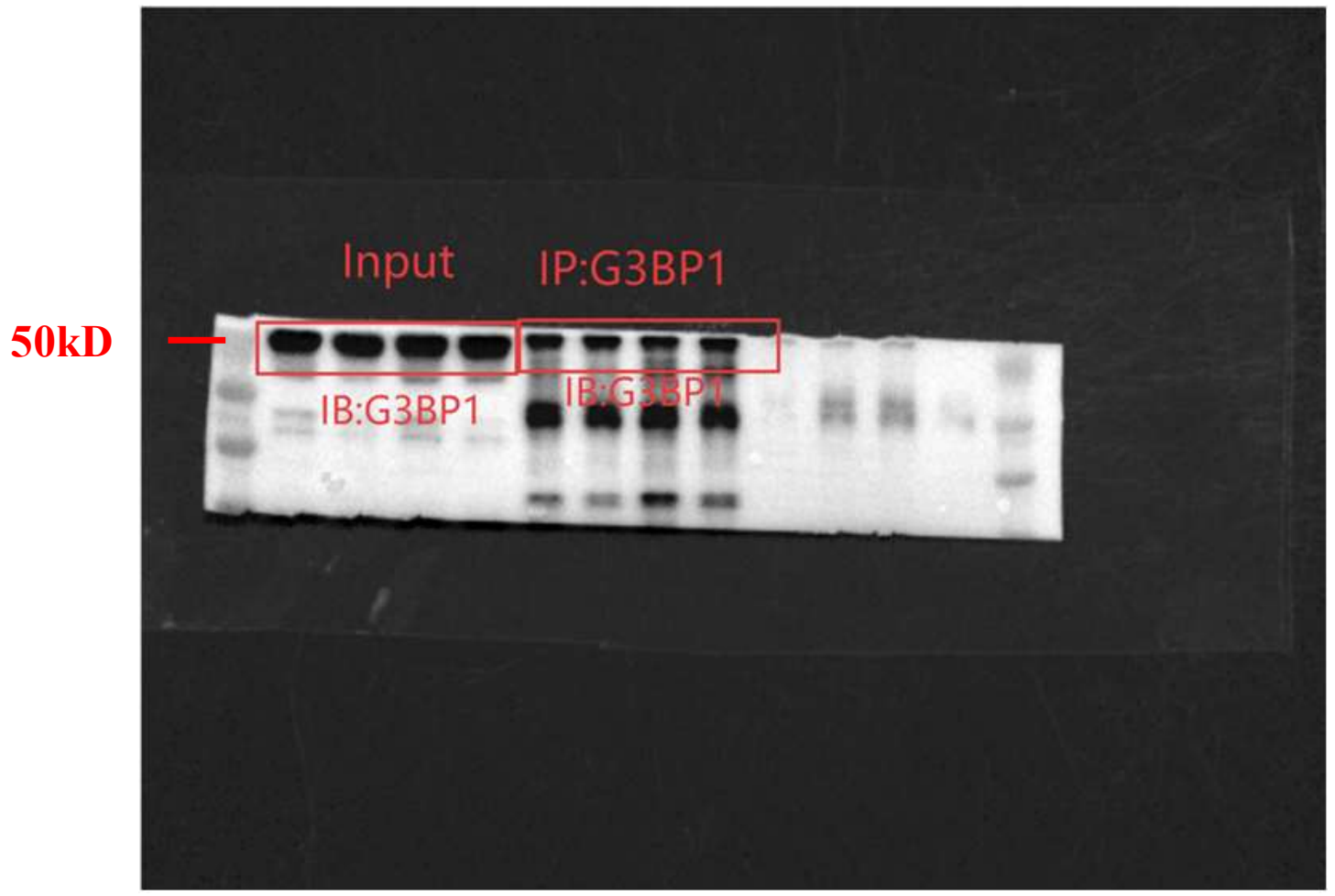
50kD
Input
IP:G3BP1
IB:G3BP1
IB:G3BP1

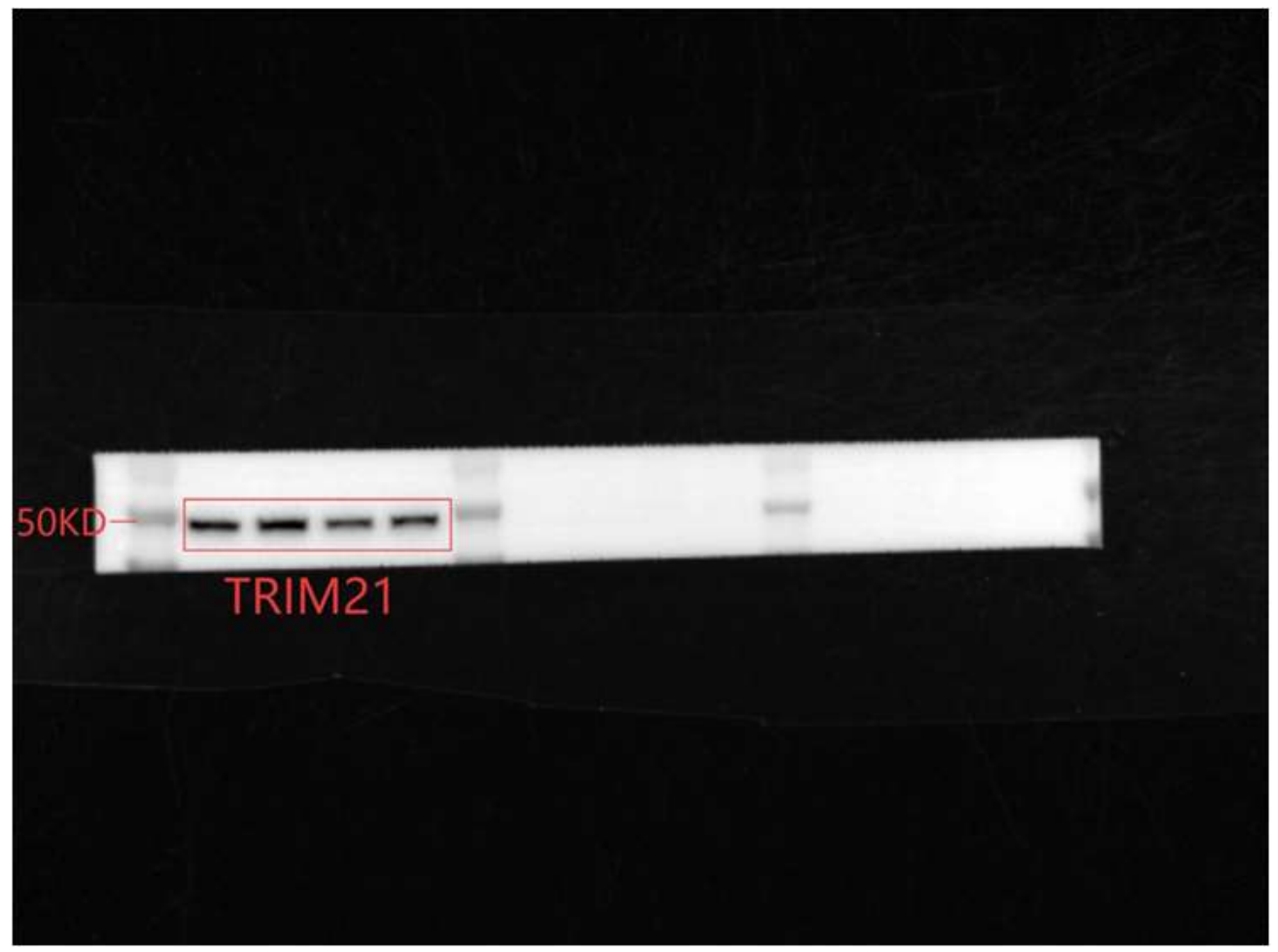
50KD
TRIM21

Figure J

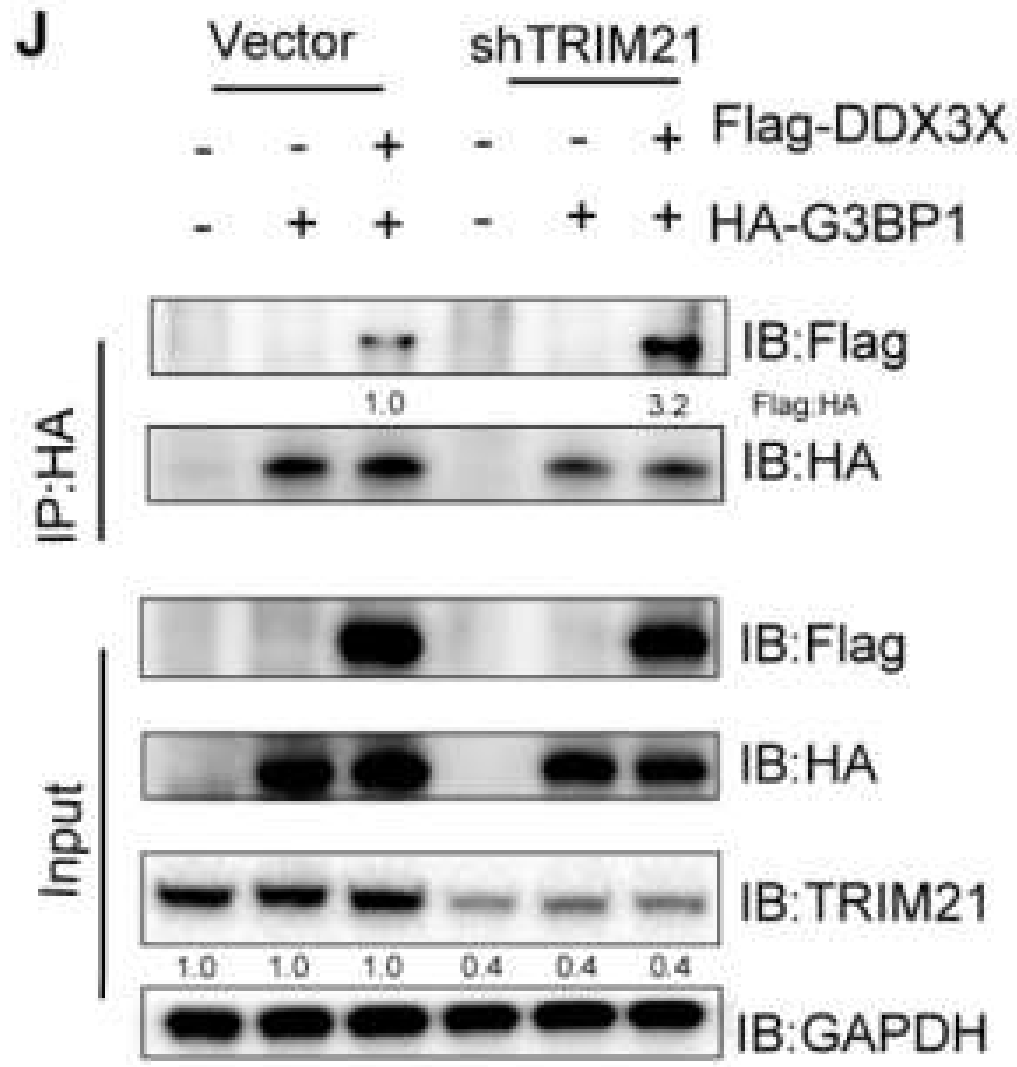
J
Vector
shTRIM21
- - + - - + Flag-DDX3X
- + + - + + HA-G3BP1
IP:HA
IB:Flag
1.0
3.2
Flag:HA
IB:HA
Input
IB:Flag
IB:HA
IB:TRIM21
1.0 1.0 1.0 0.4 0.4 0.4
IB:GAPDH

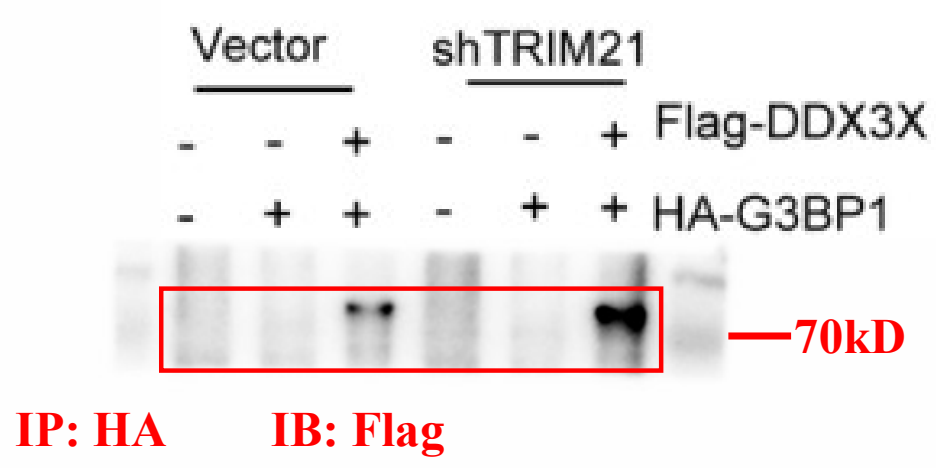
Vector
shTRIM21
- - + - - + Flag-DDX3X
- + + - + + HA-G3BP1
70kD
IP: HA
IB: Flag

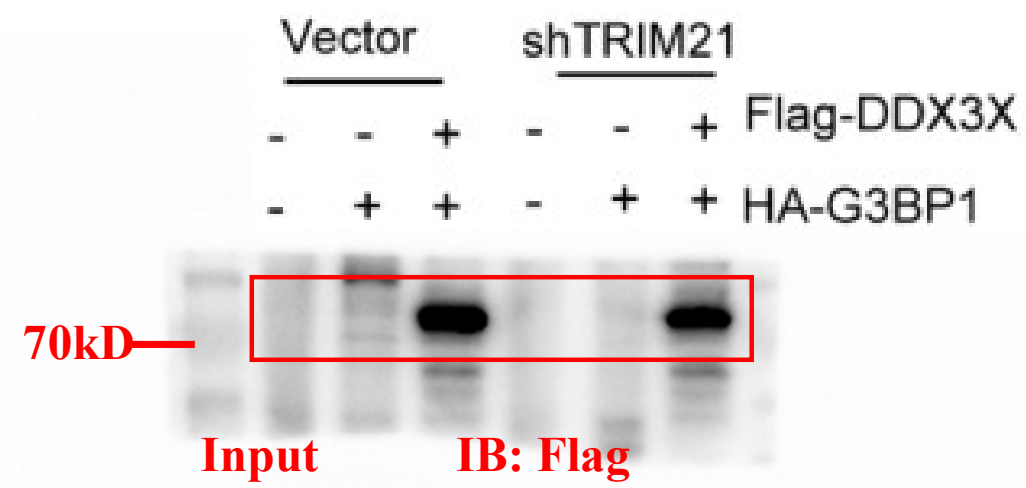
Vector
shTRIM21
- - + - - + Flag-DDX3X
- + + - + + HA-G3BP1
70kD
Input
IB: Flag

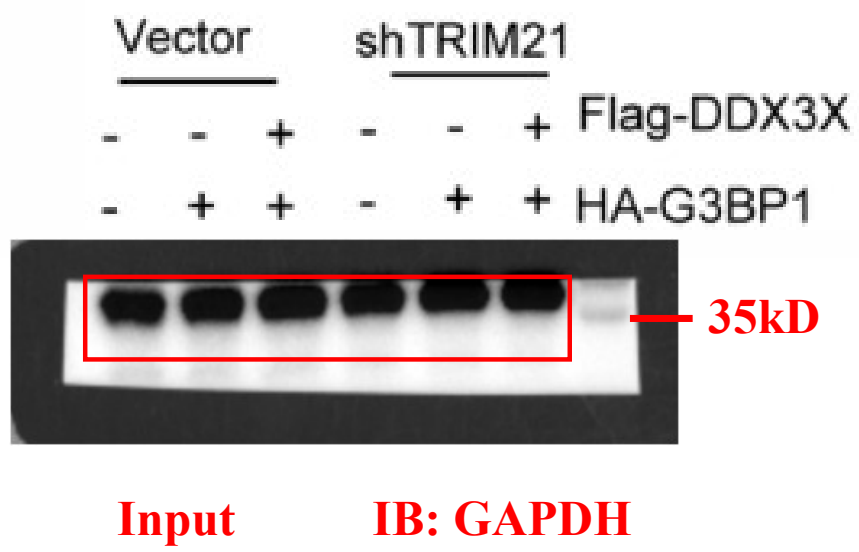
Vector
shTRIM21
- - + - - + Flag-DDX3X
- + + - + + HA-G3BP1
35kD
Input
IB: GAPDH

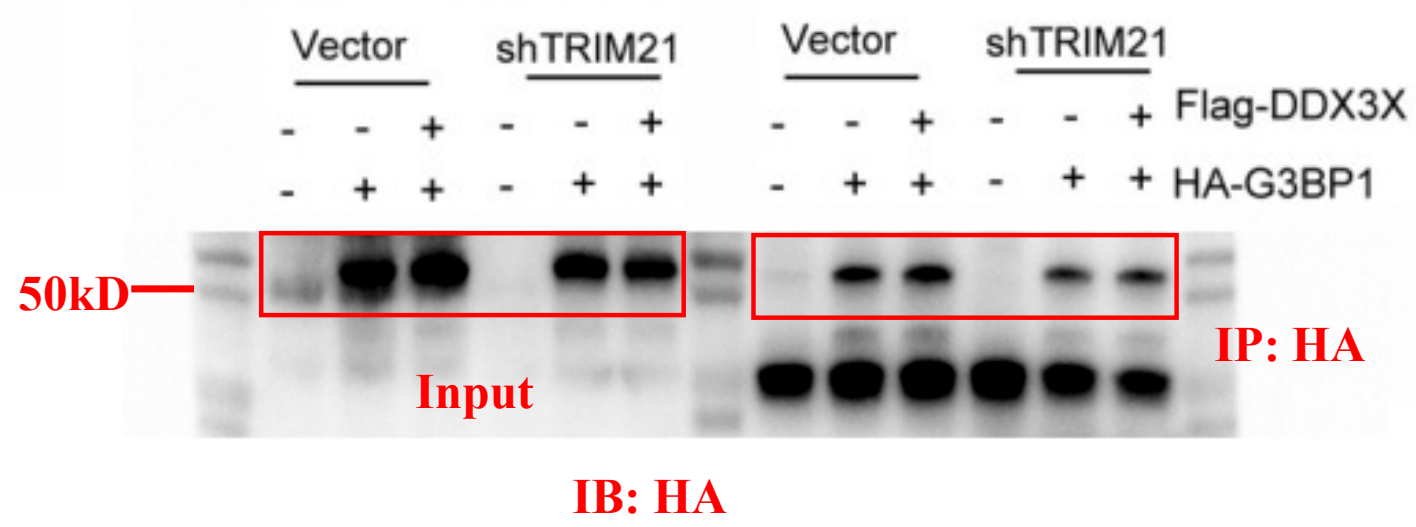
Vector
shTRIM21
Vector
shTRIM21
- - + - - + - - + - - + Flag-DDX3X
- + + - + + - + + - + + HA-G3BP1
50kD
Input
IP: HA
IB: HA

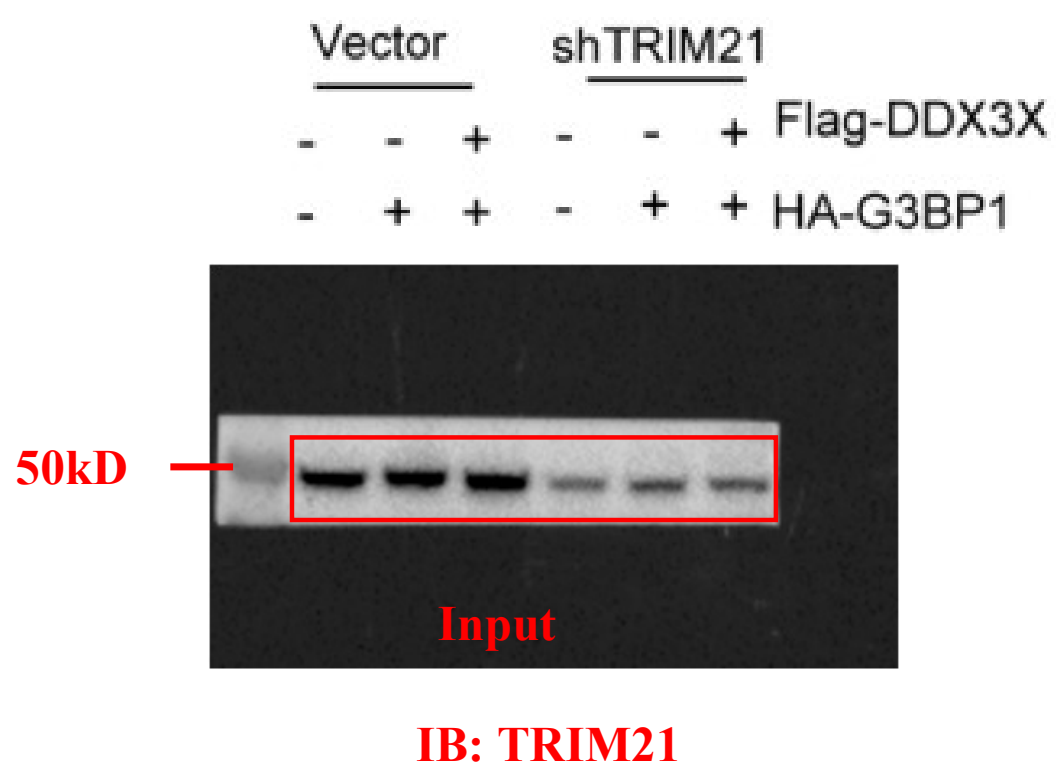
Vector
shTRIM21
- - + - - + Flag-DDX3X
- + + - + + HA-G3BP1
50kD
Input
IB: TRIM21

Figure 5B

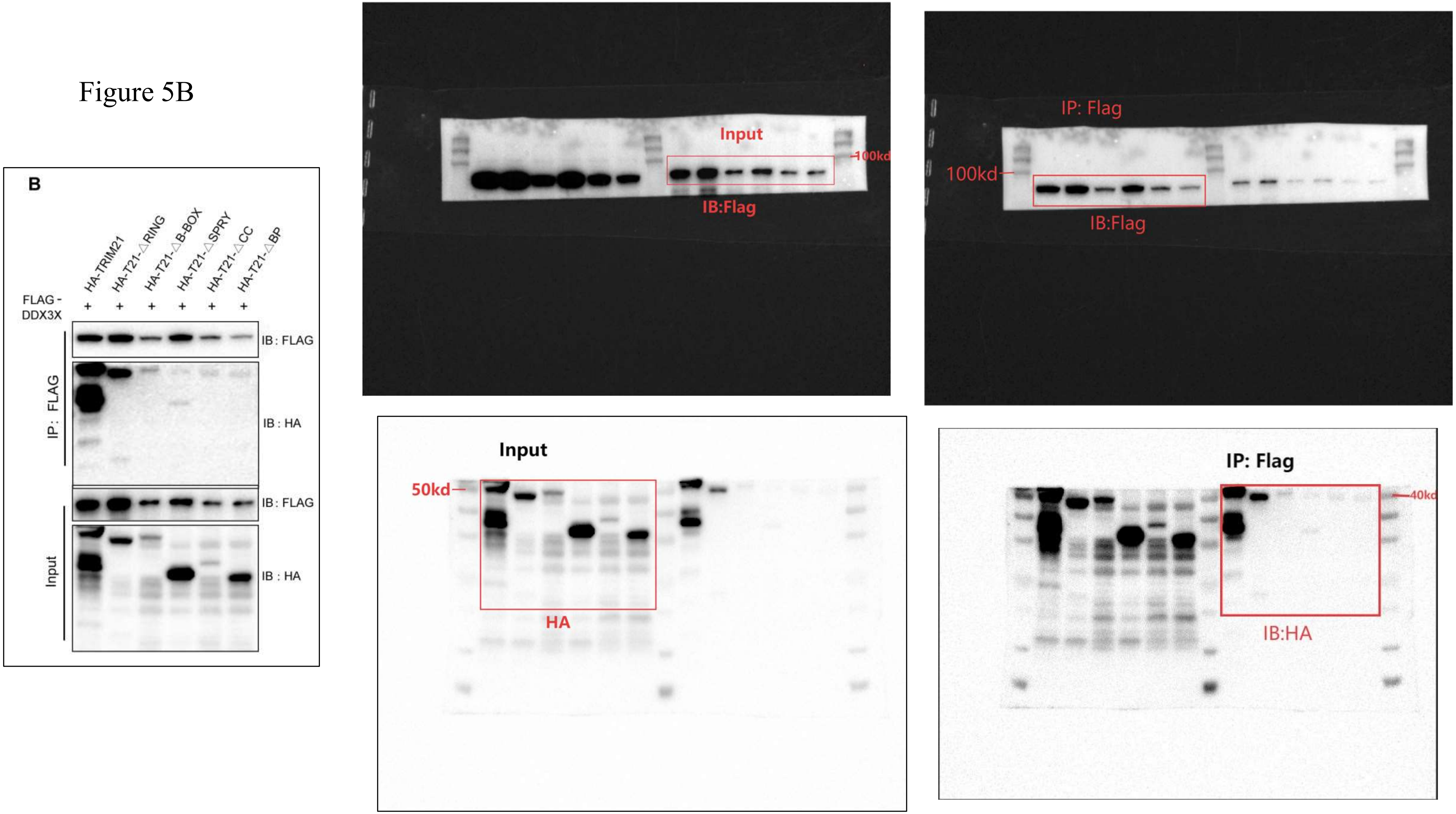
B
HA-TRIM21
HA-T21-△RING
HA-T21-△B-BOX
HA-T21-△SPRY
HA-T21-△CC
HA-T21-△BP
FLAG - DDX3X
IB : FLAG
IP : FLAG
IB : HA
IB : FLAG
Input
IB : HA
Input
100kd
IB:Flag
IP: Flag
100kd
IB:Flag
Input
50kd
HA
IP: Flag
40kd
IB:HA

Figure 5F

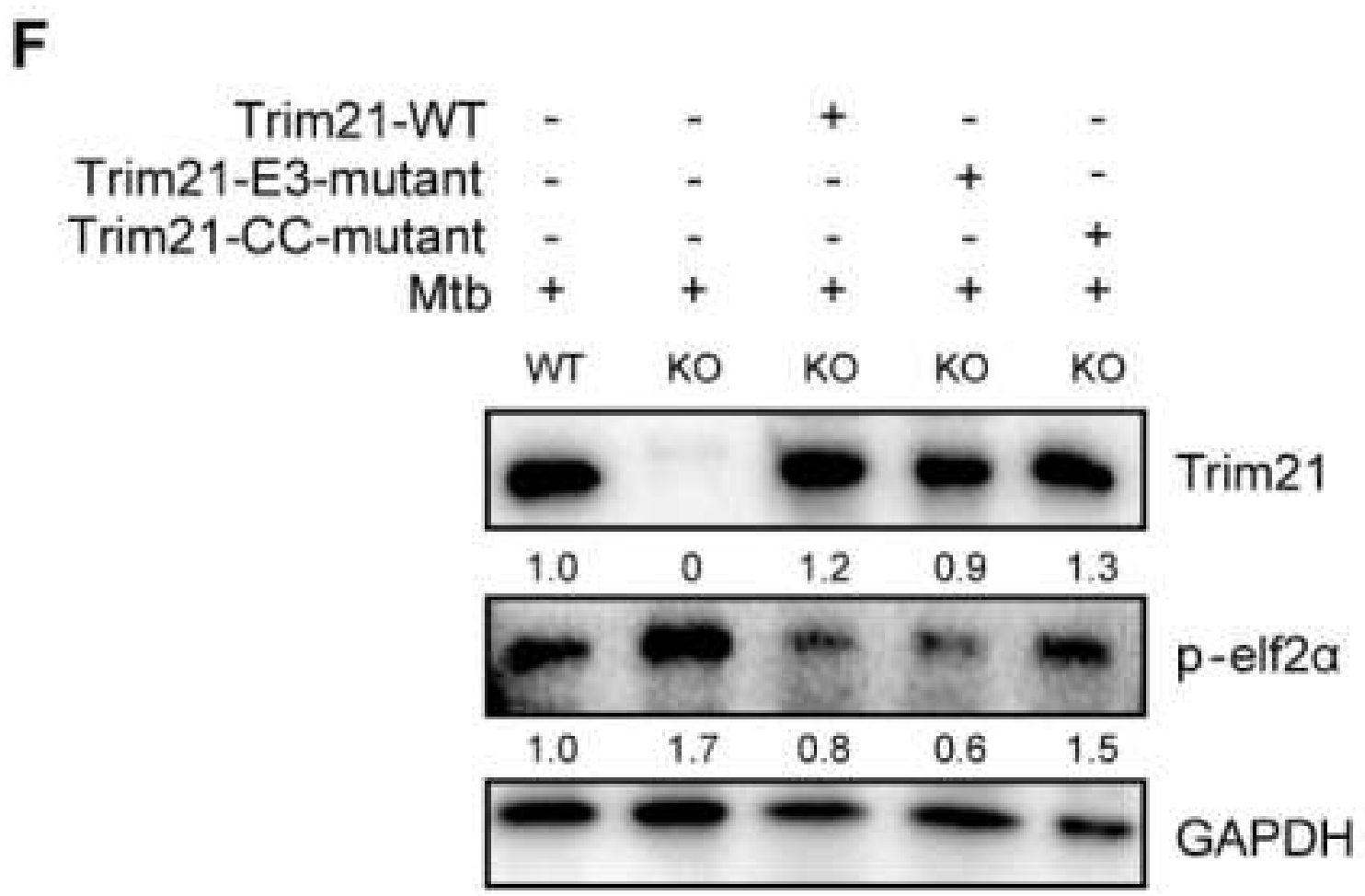

F
Trim21-WT - - + - -
Trim21-E3-mutant - - - + -
Trim21-CC-mutant - - - - +
Mtb + + + + +
WT KO KO KO KO
Trim21
1.0 0 1.2 0.9 1.3
p-elf2α
1.0 1.7 0.8 0.6 1.5
GAPDH

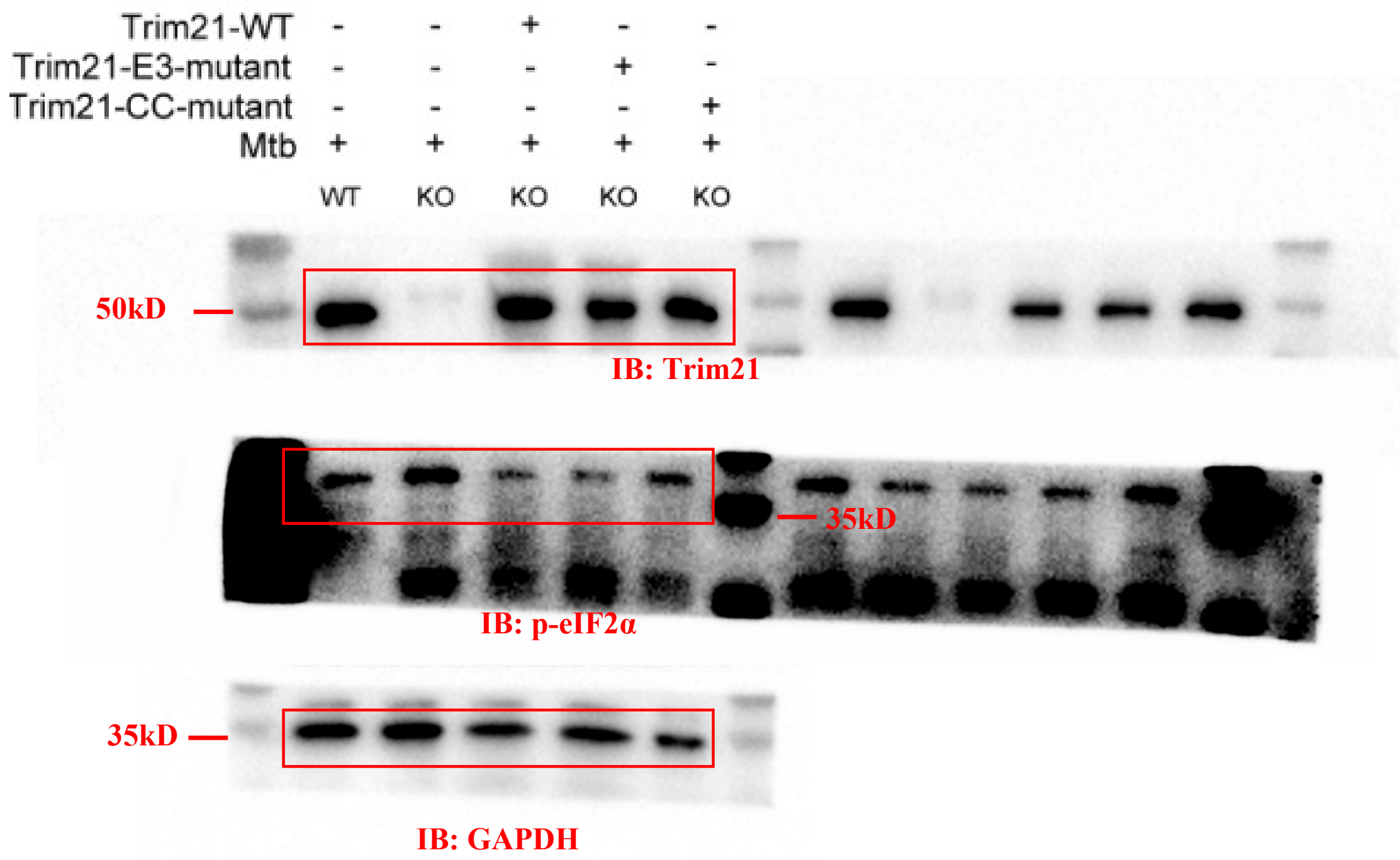

Trim21-WT - - + - -
Trim21-E3-mutant - - - + -
Trim21-CC-mutant - - - - +
Mtb + + + + +
WT KO KO KO KO
50kD
IB: Trim21
35kD
IB: p-eIF2α
35kD
IB: GAPDH

Figure 6A

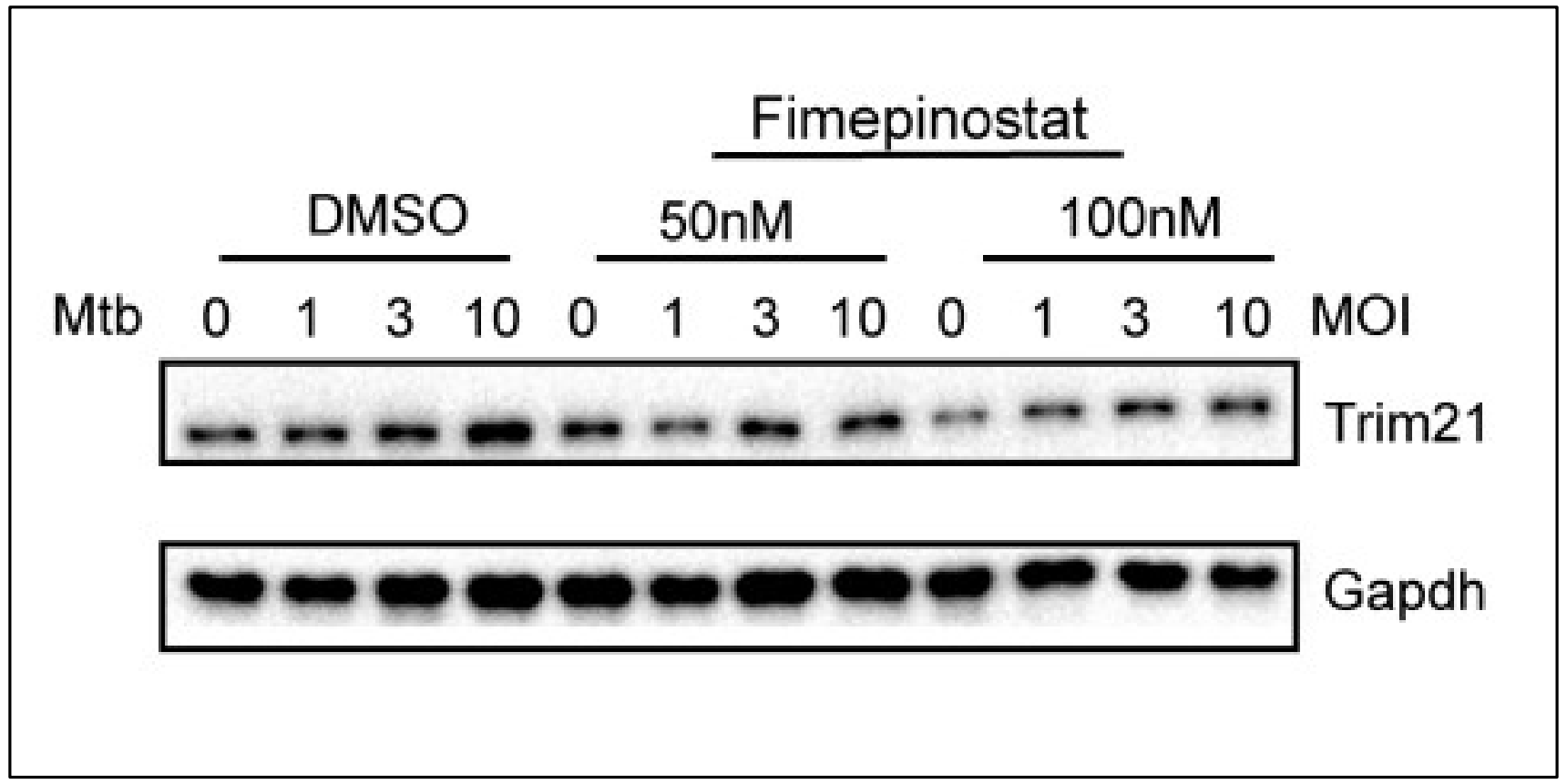
Fimepinostat
DMSO
50nM
100nM
Mtb 0 1 3 10 0 1 3 10 0 1 3 10 MOI
Trim21
Gapdh

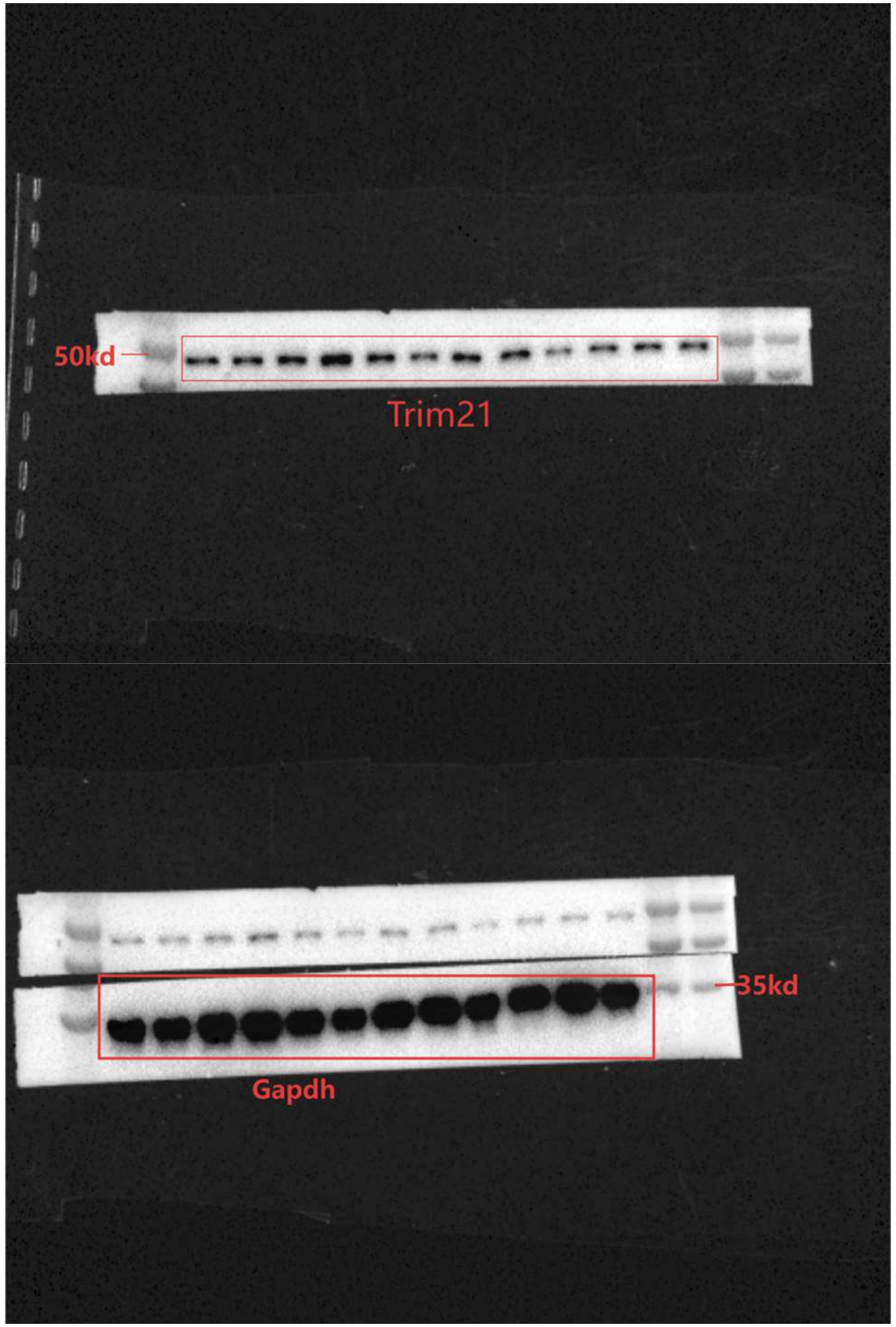
50kd
Trim21
35kd
Gapdh

Figure 6E

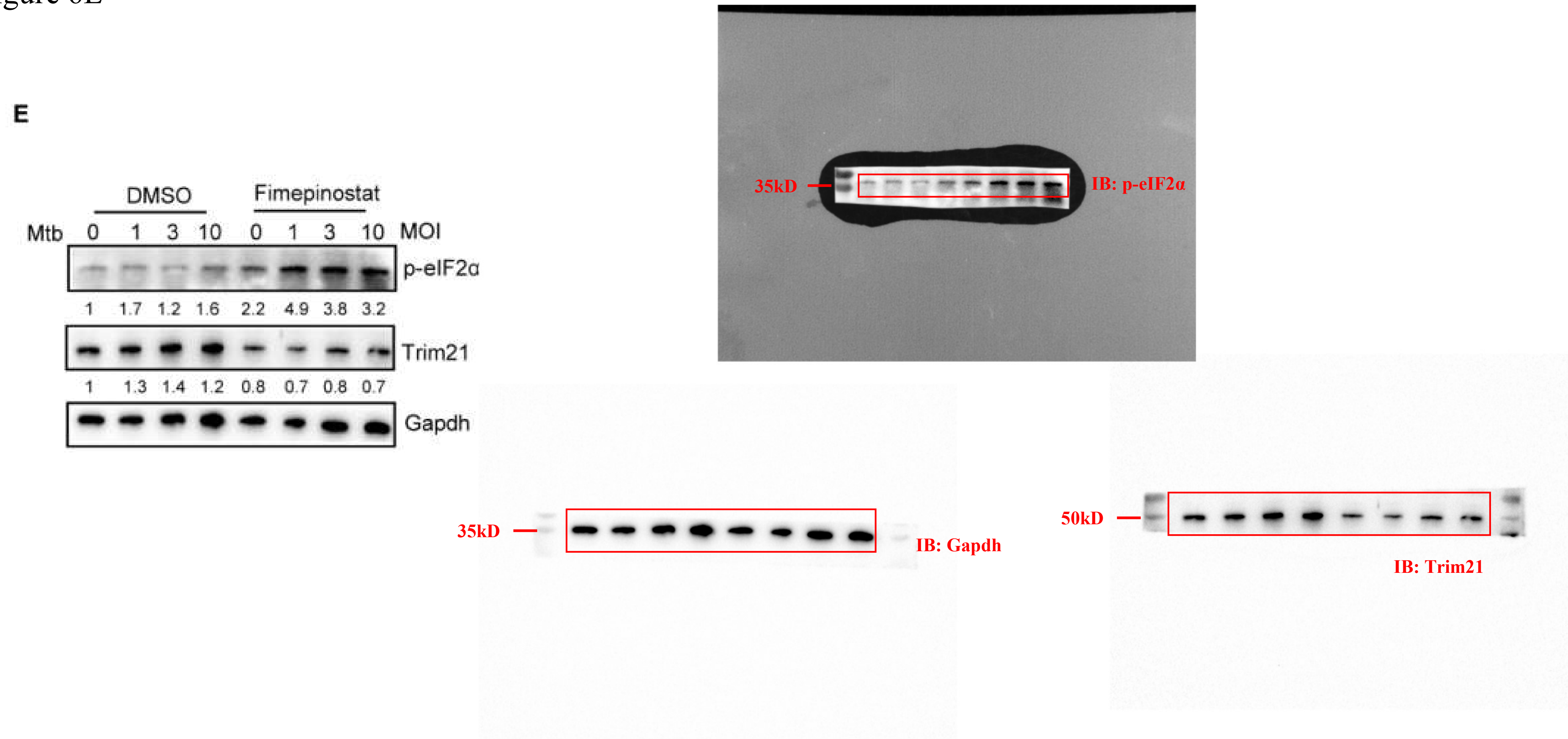

E
DMSO
Fimepinostat
Mtb 0 1 3 10 0 1 3 10 MOI
p-eIF2α
1 1.7 1.2 1.6 2.2 4.9 3.8 3.2
Trim21
1 1.3 1.4 1.2 0.8 0.7 0.8 0.7
Gapdh
35kD
IB: p-eIF2α
35kD
IB: Gapdh
50kD
IB: Trim21

Figure S1F

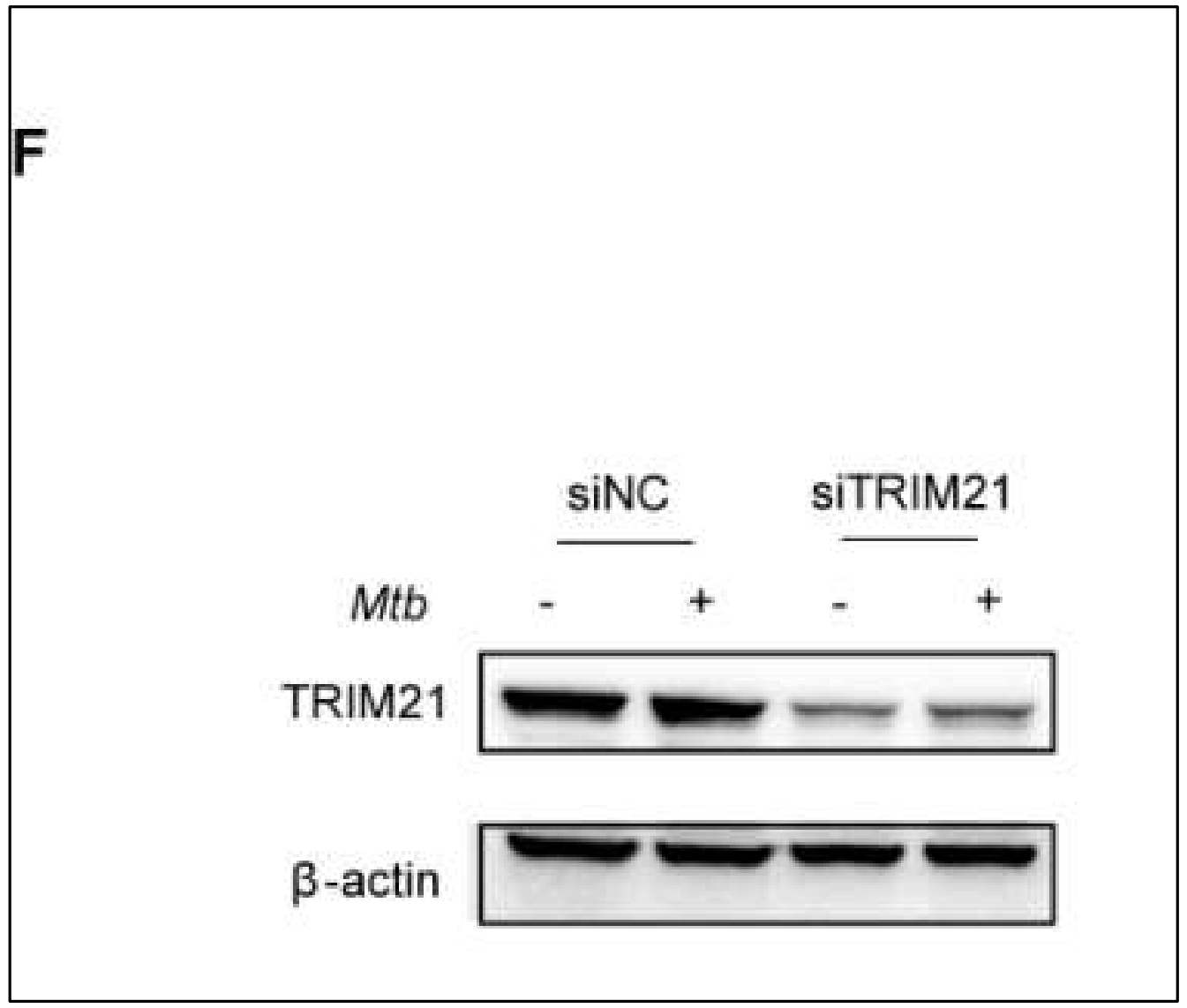

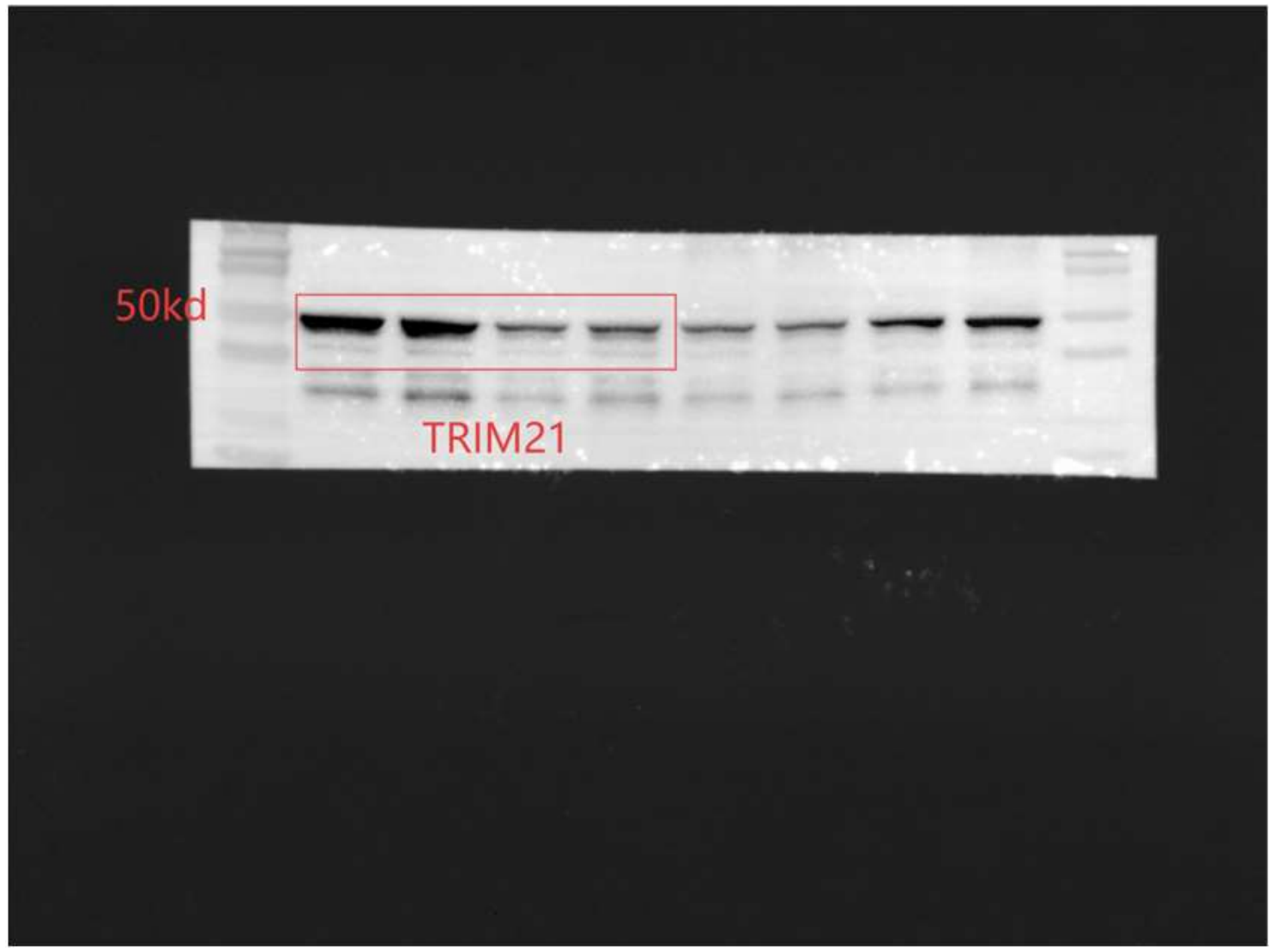

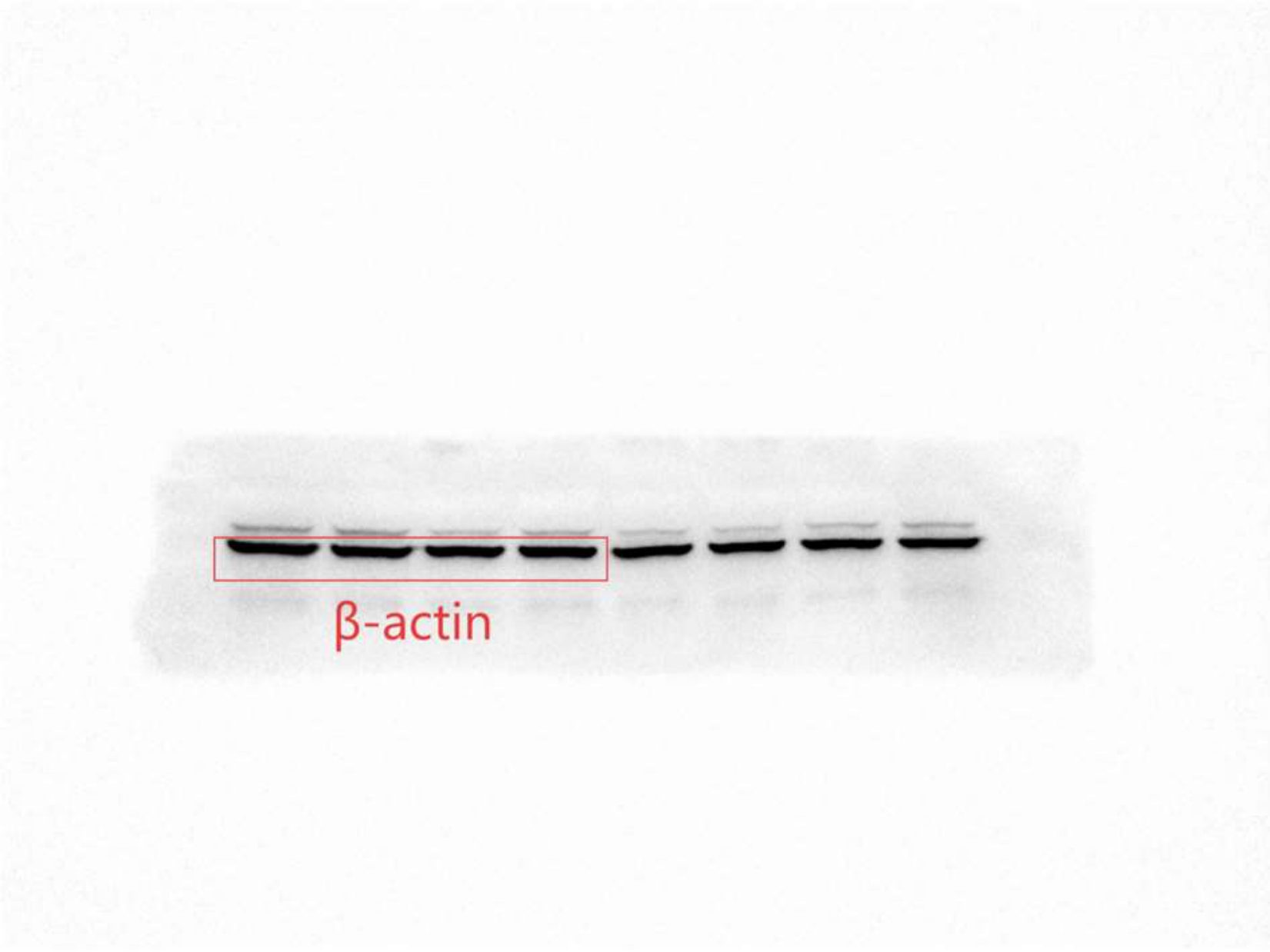

Figure S1G

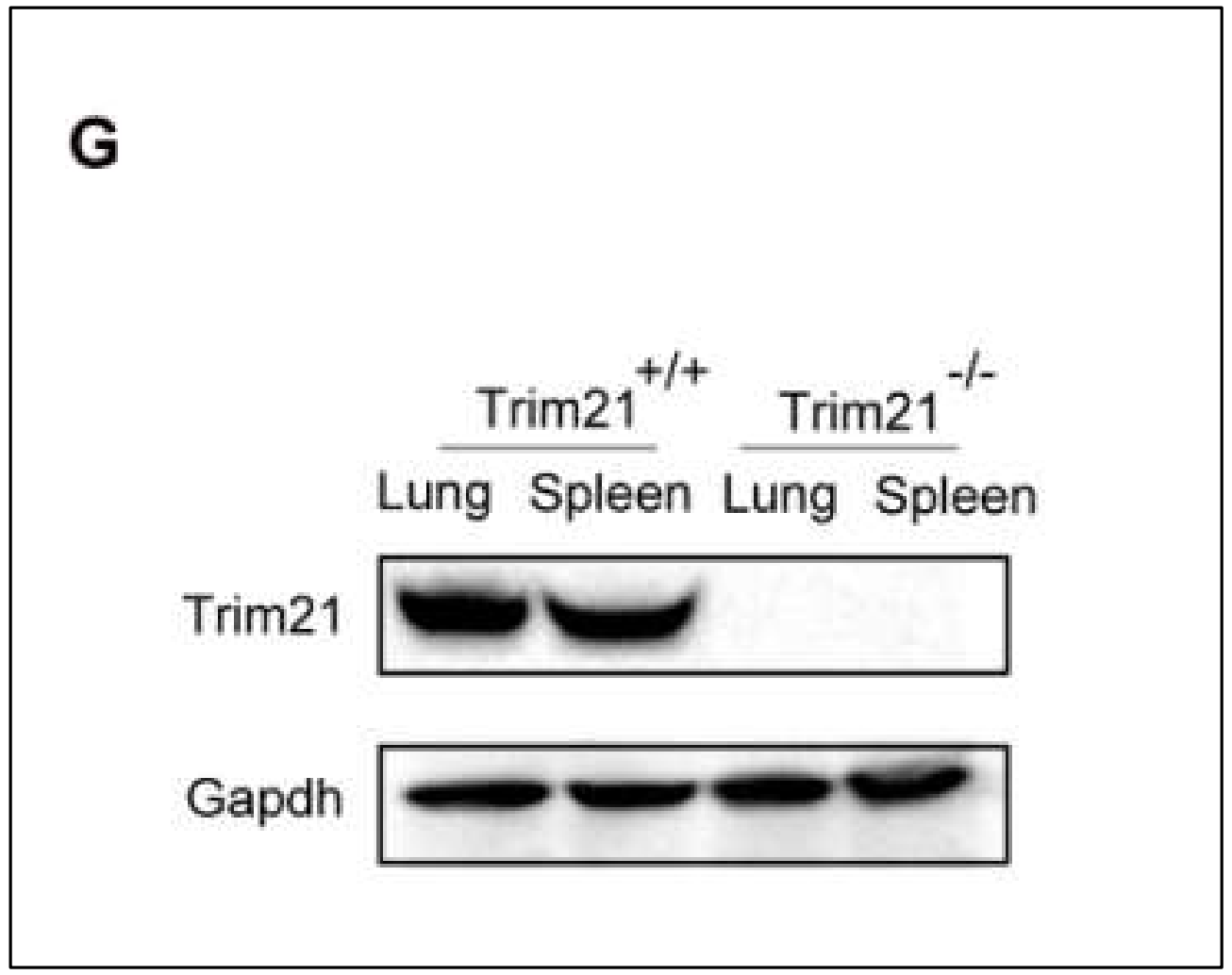
G
Trim21+/+
Trim21-/-
Lung
Spleen
Lung
Spleen
Trim21
Gapdh

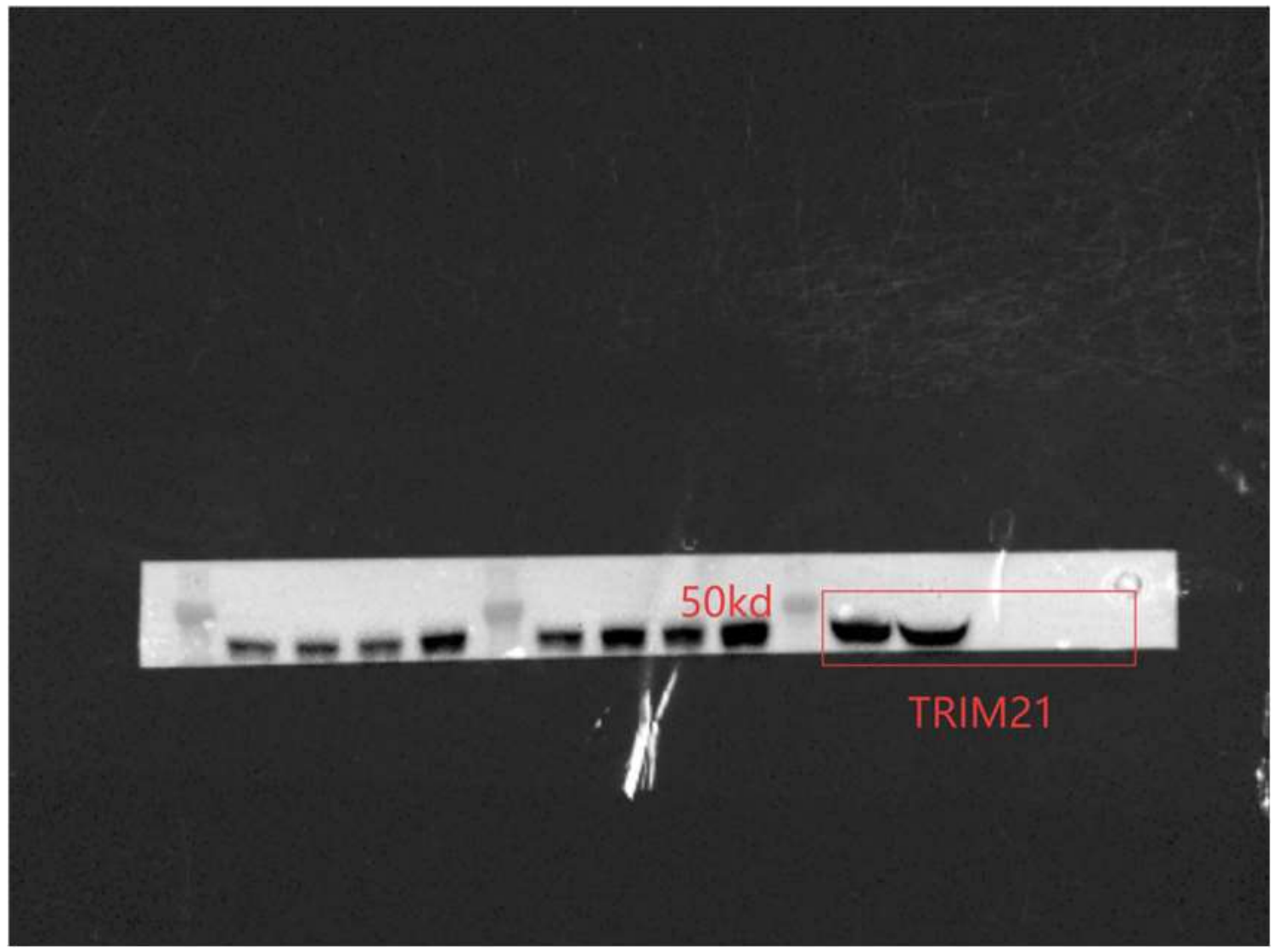
50kd
TRIM21

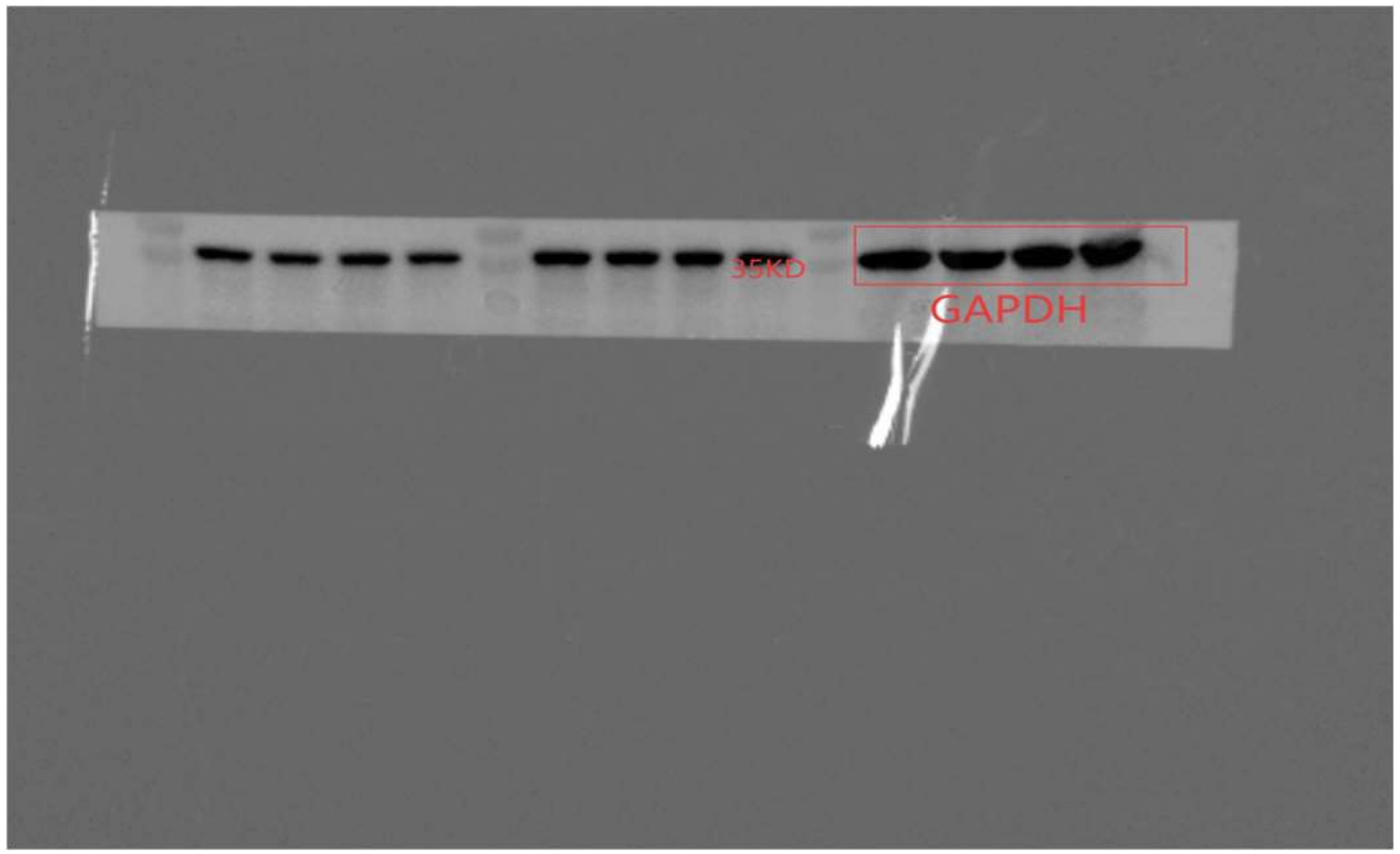
35KD
GAPDH

Figure S3A

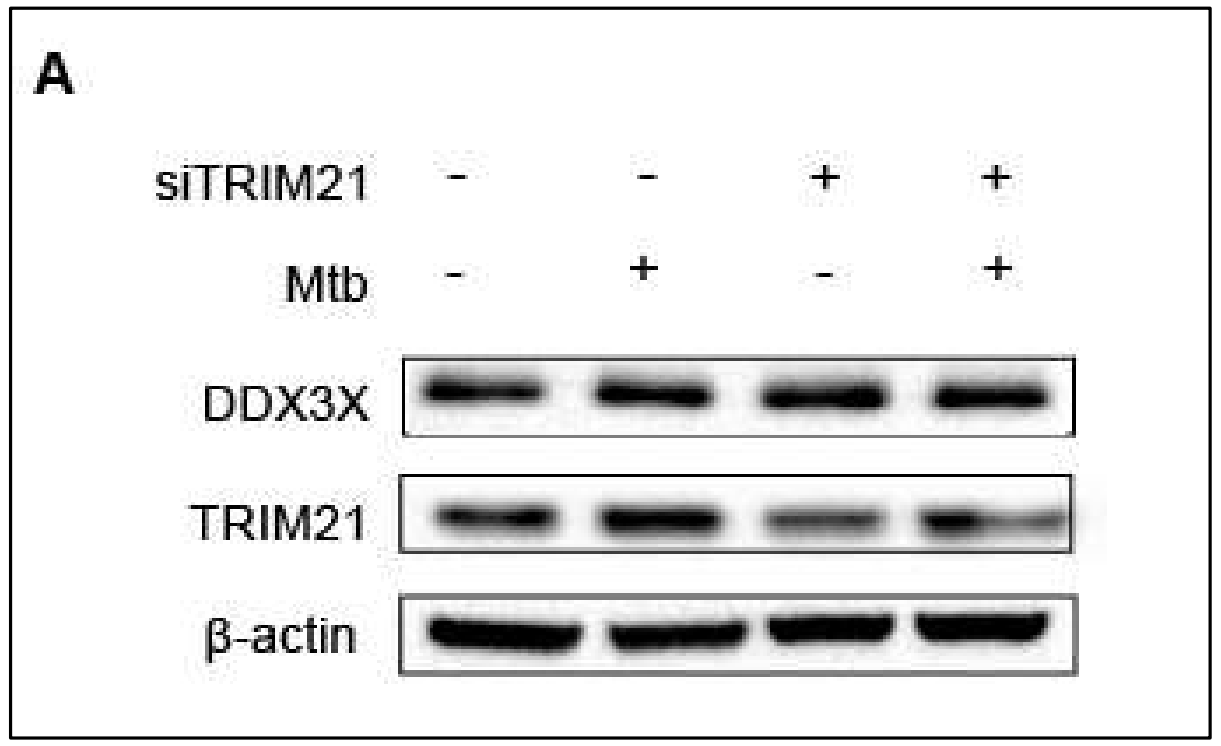

A
siTRIM21 - - + +
Mtb - + - +
DDX3X
TRIM21
β-actin

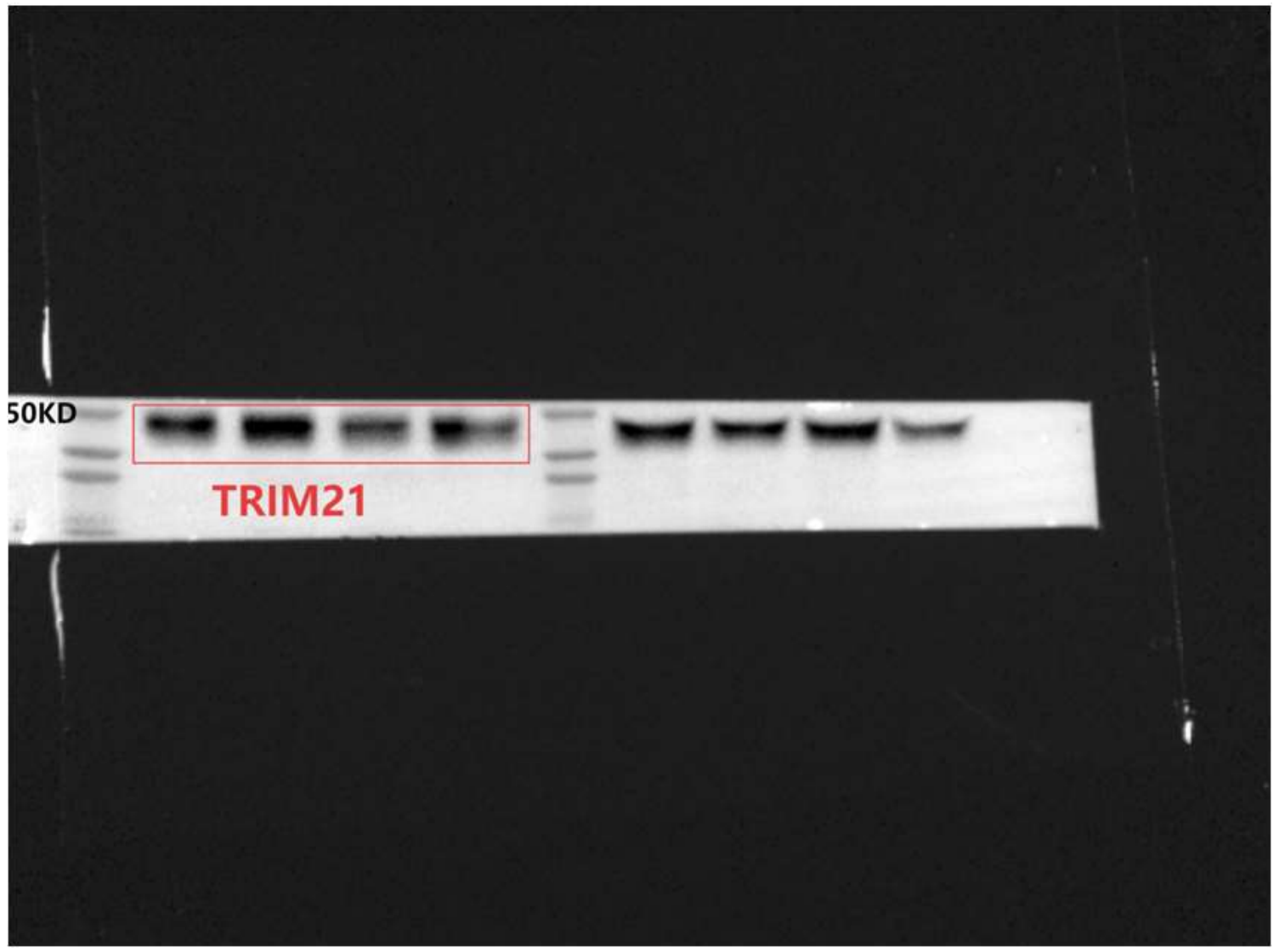

50KD
TRIM21

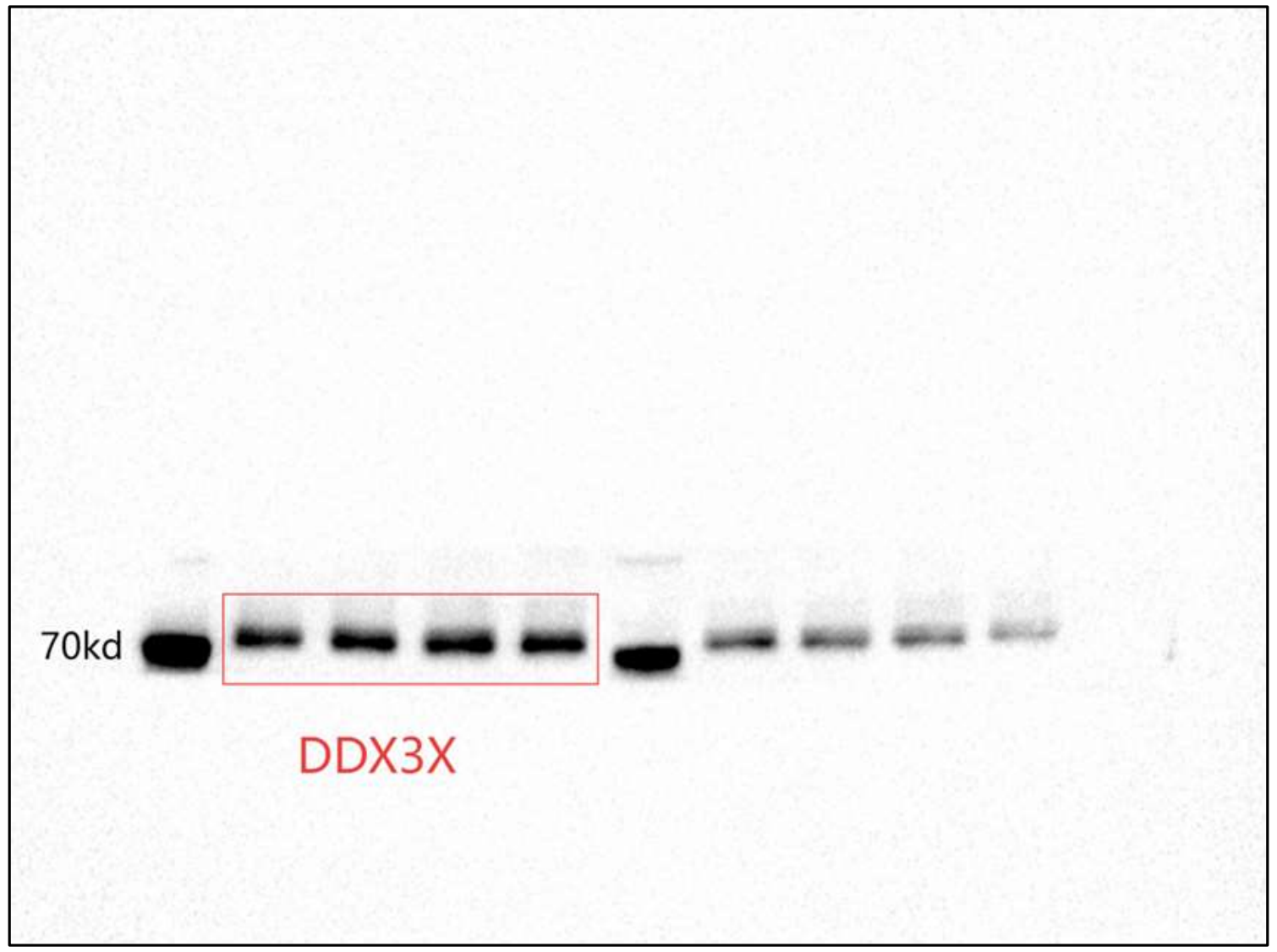

70kd
DDX3X

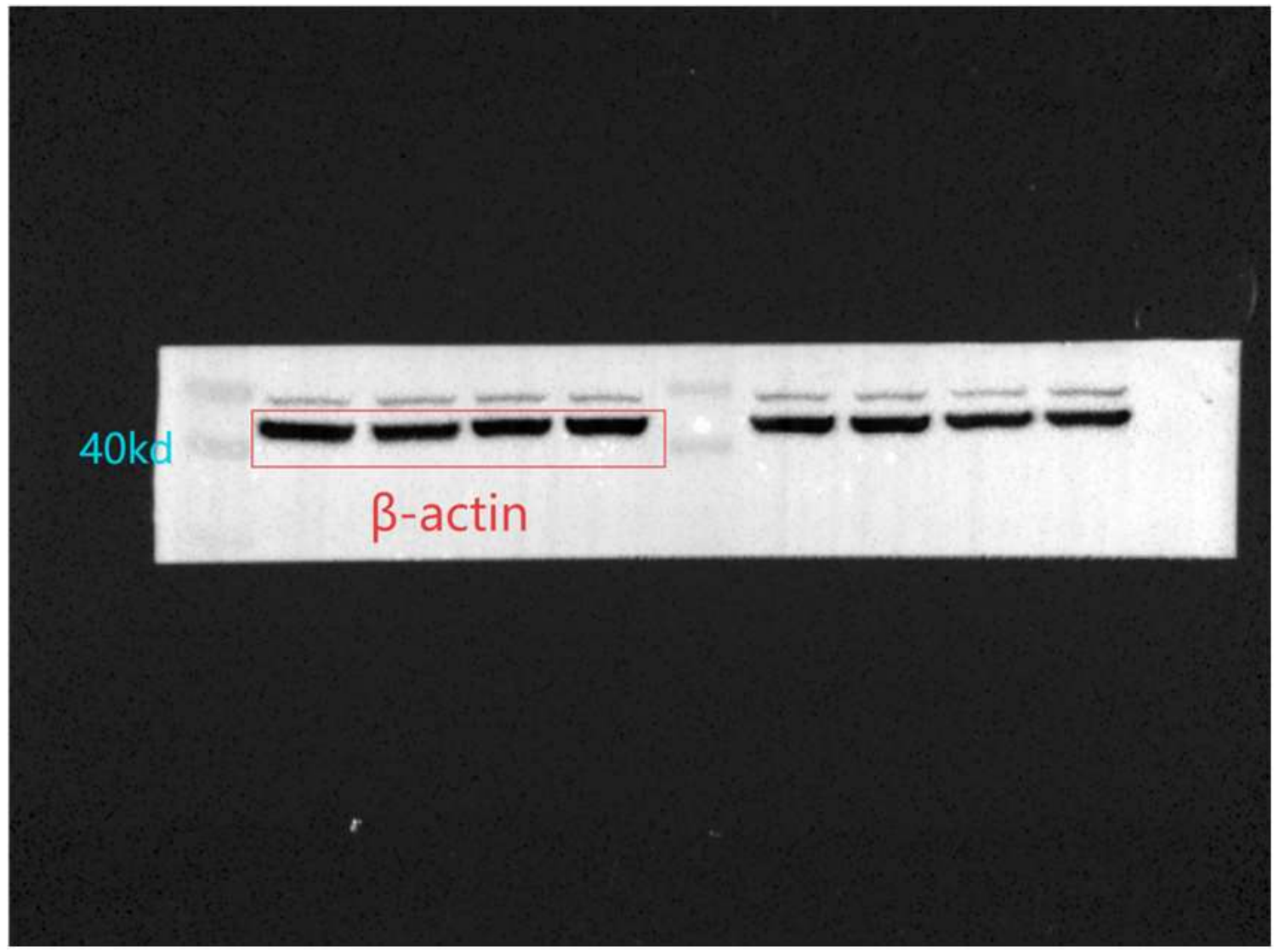

40kd
β-actin

Figure S3B

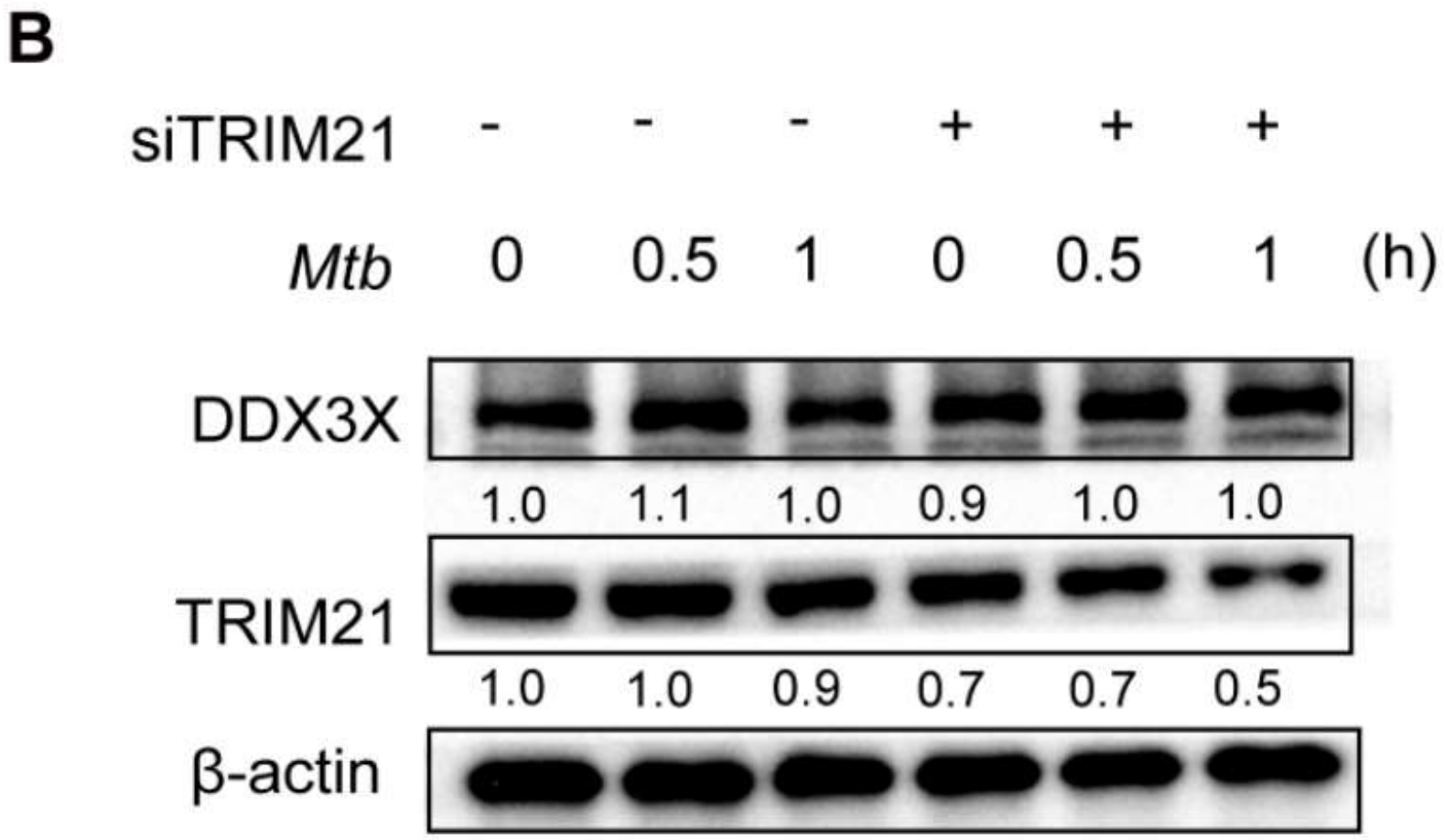

B
siTRIM21 - - - + + +
Mtb 0 0.5 1 0 0.5 1 (h)
DDX3X
1.0 1.1 1.0 0.9 1.0 1.0
TRIM21
1.0 1.0 0.9 0.7 0.7 0.5
β-actin

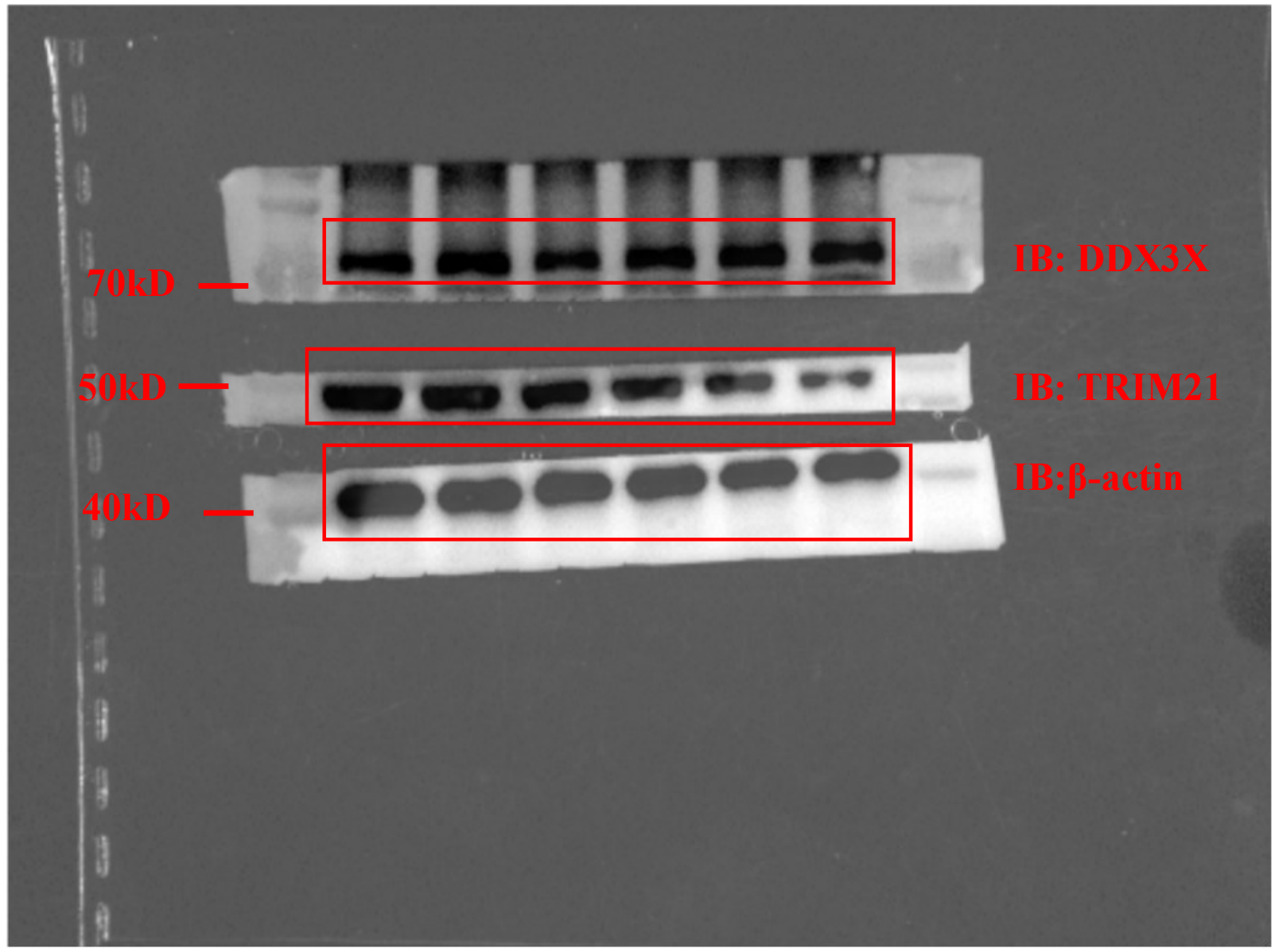

70kD
50kD
40kD
IB: DDX3X
IB: TRIM21
IB:β-actin

Figure S3C

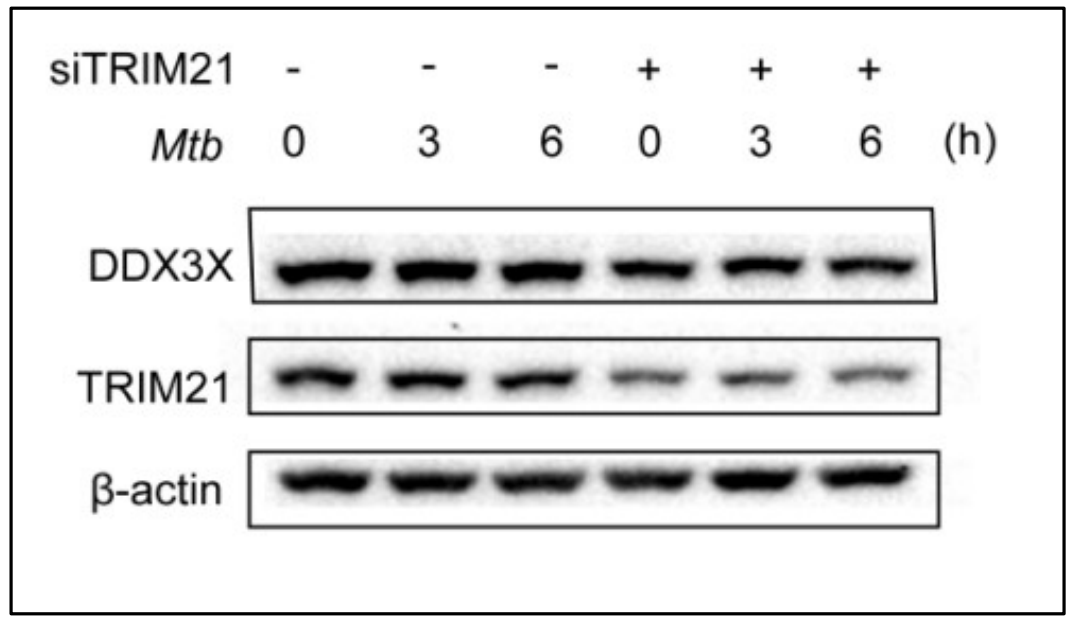
siTRIM21 - - - + + +
Mtb 0 3 6 0 3 6 (h)
DDX3X
TRIM21
β-actin

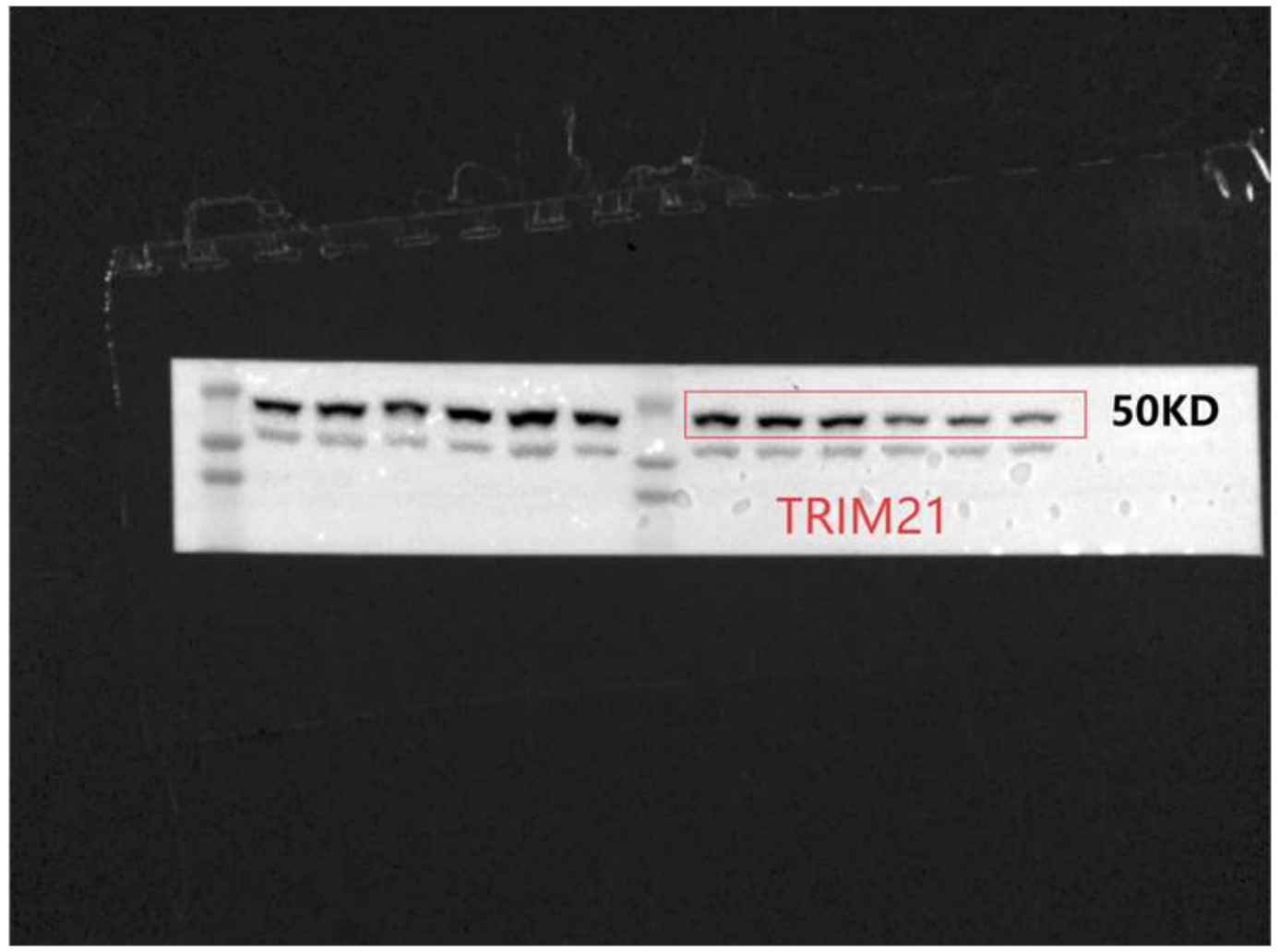
50KD
TRIM21

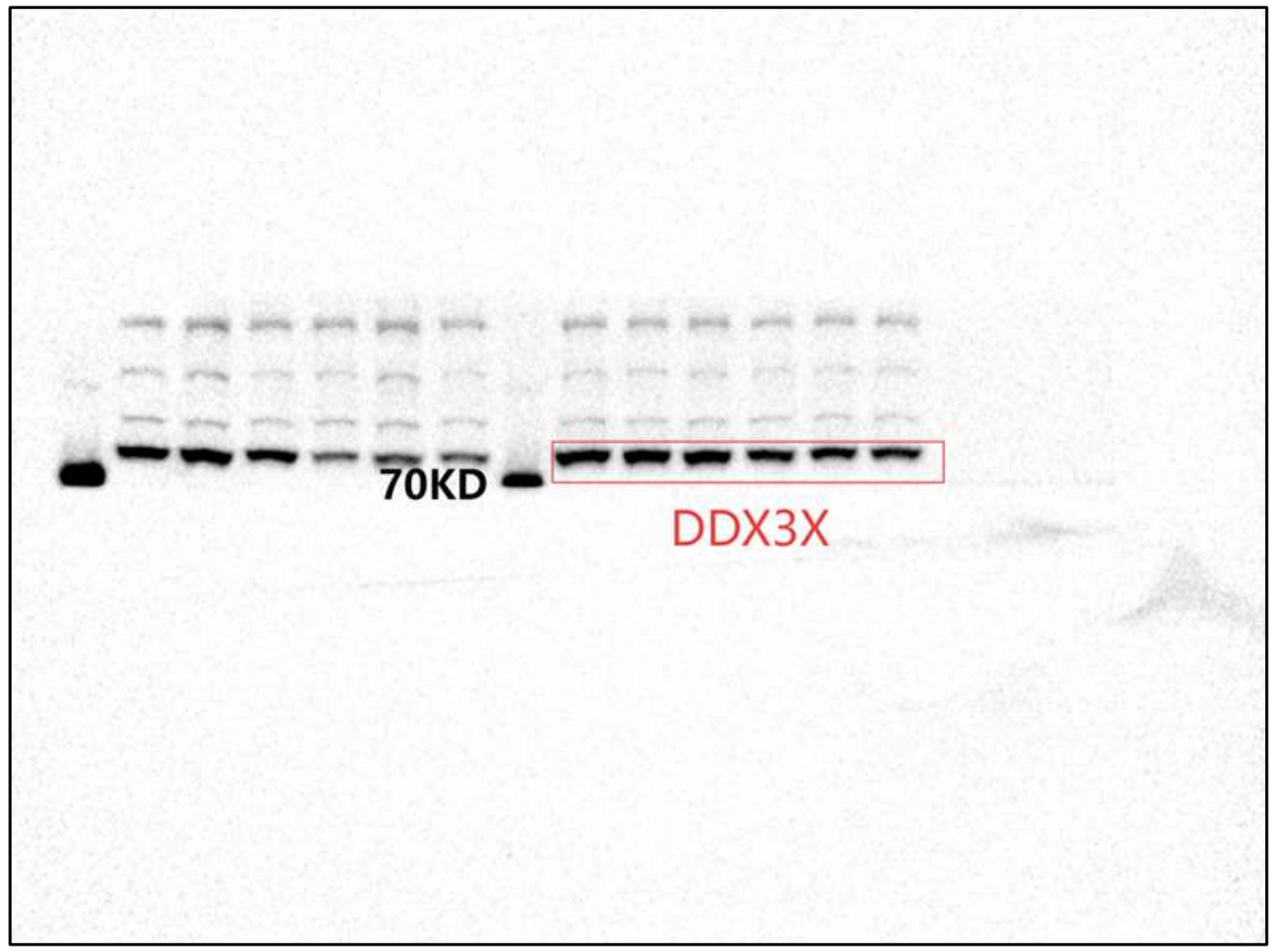
70KD
DDX3X

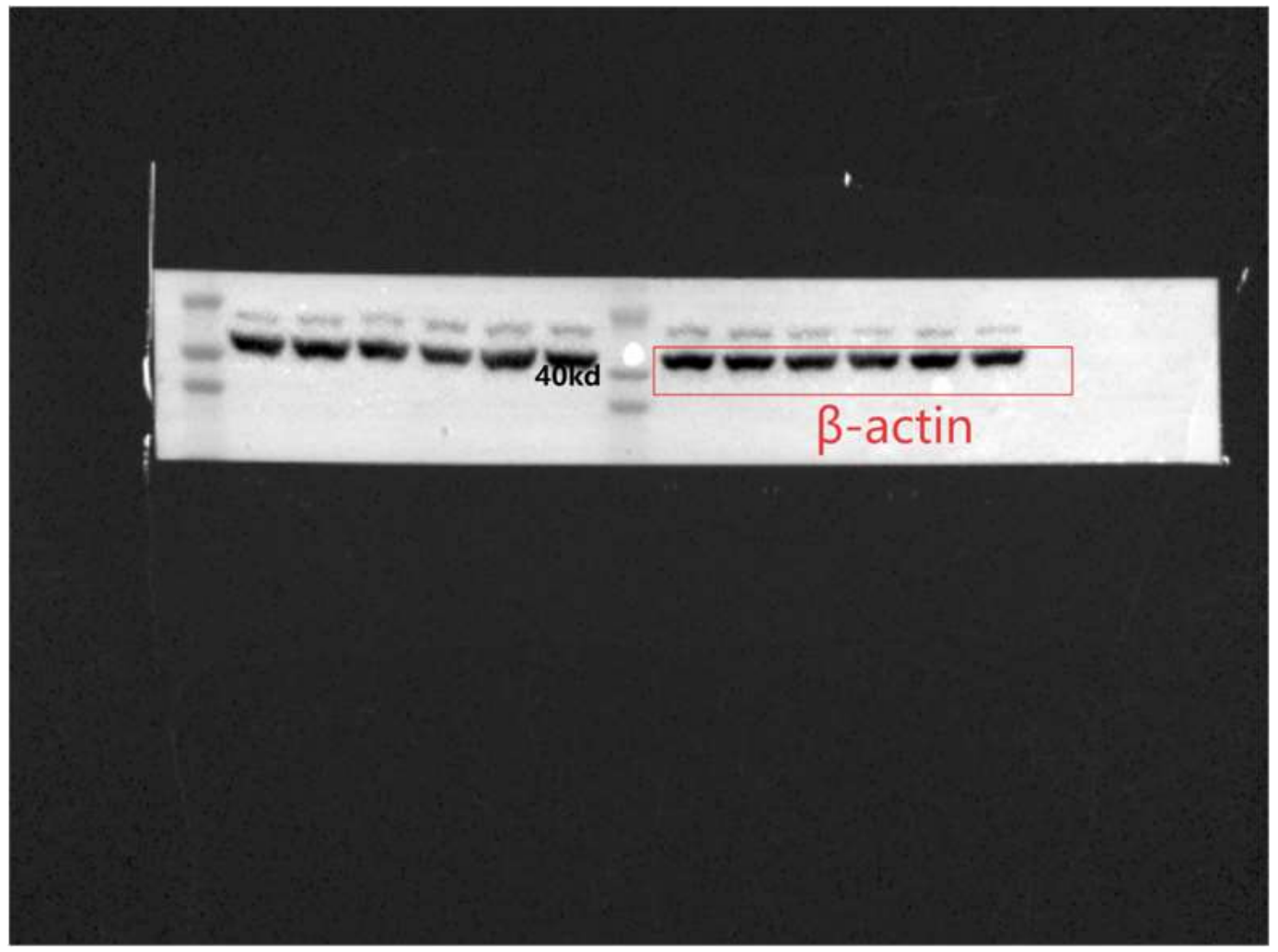
40kd
β-actin

Figure S3D

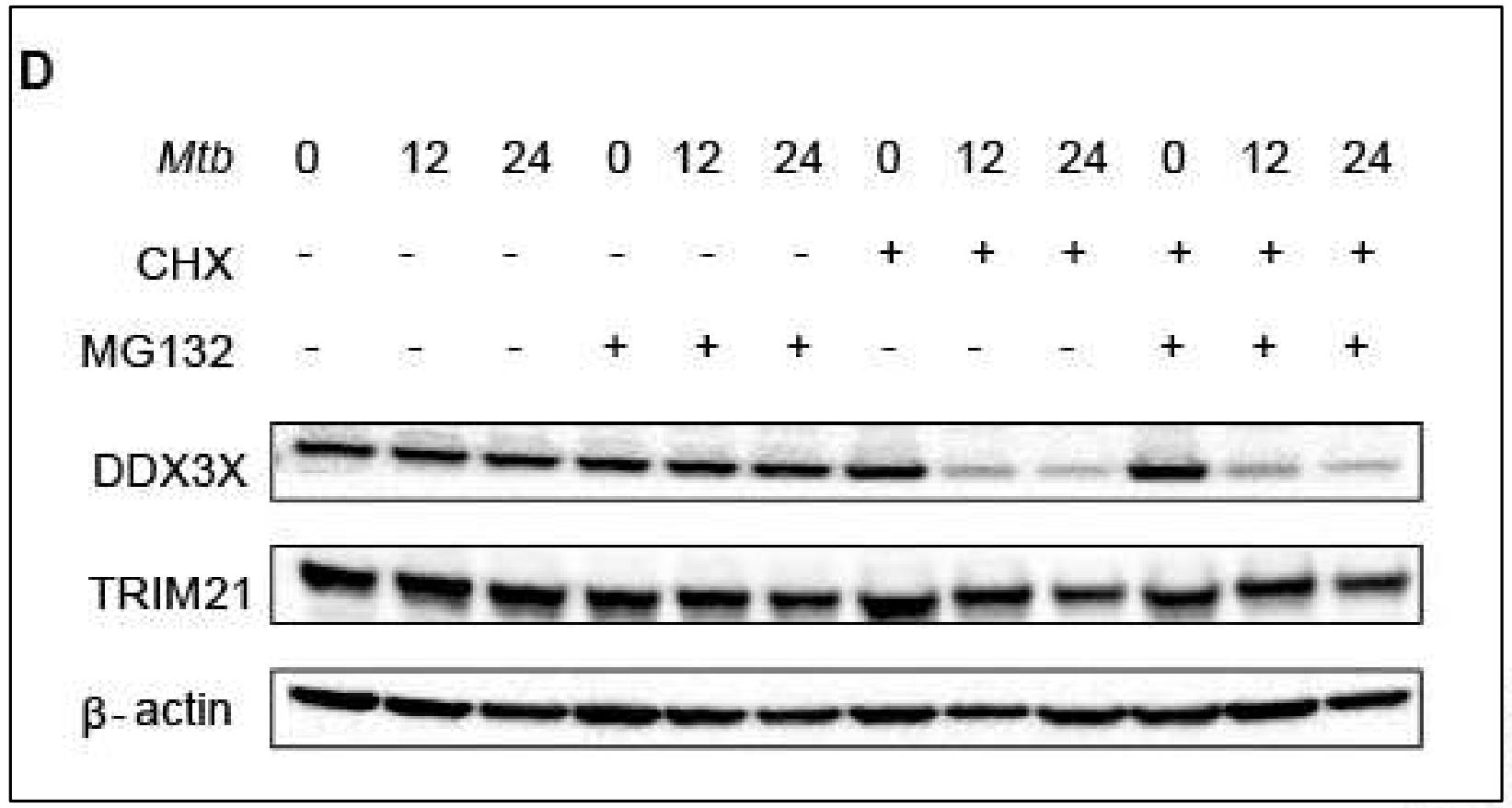
D
Mtb 0 12 24 0 12 24 0 12 24 0 12 24
CHX - - - - - - + + + + + +
MG132 - - - + + + - - - + + +
DDX3X
TRIM21
β-actin

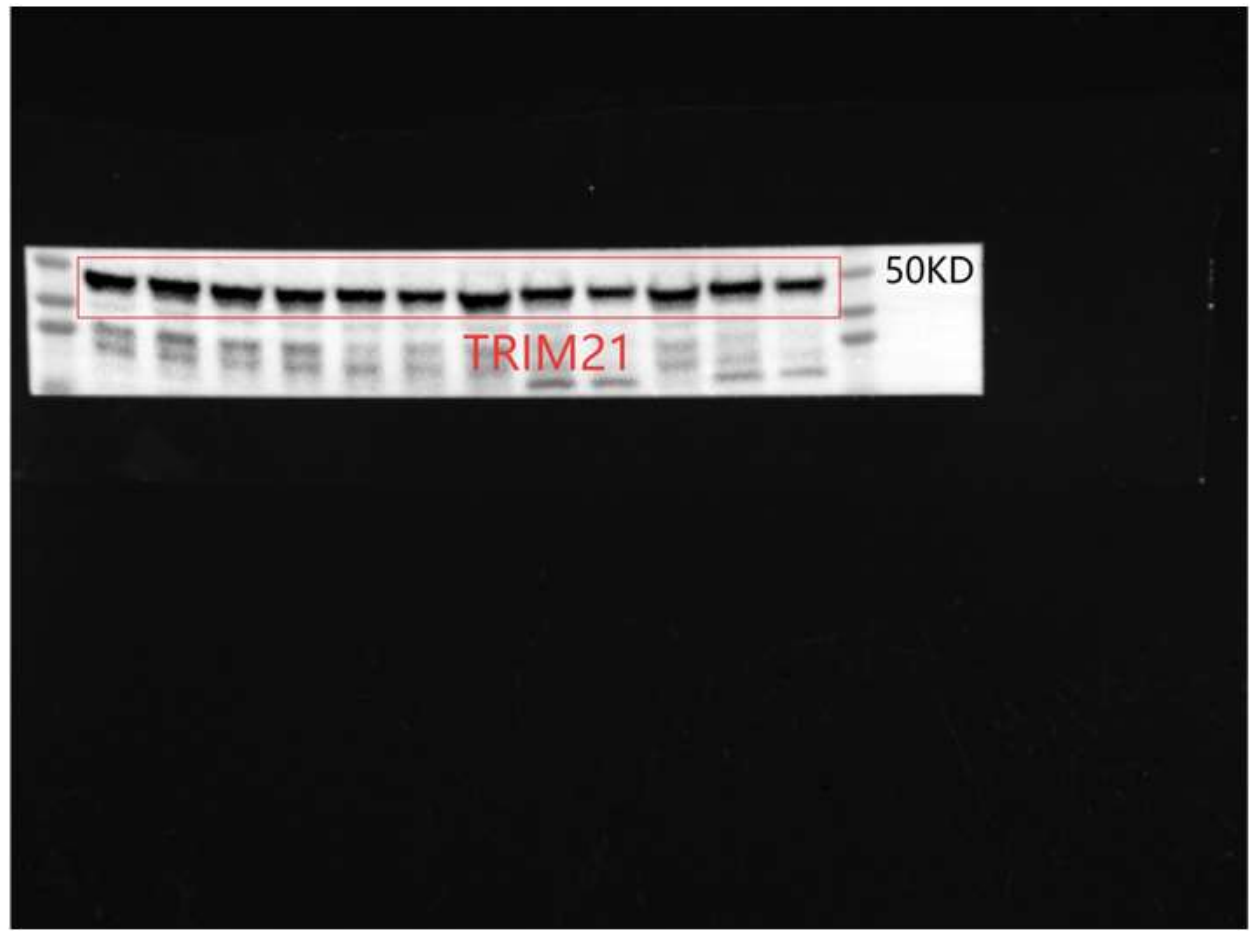
50KD
TRIM21

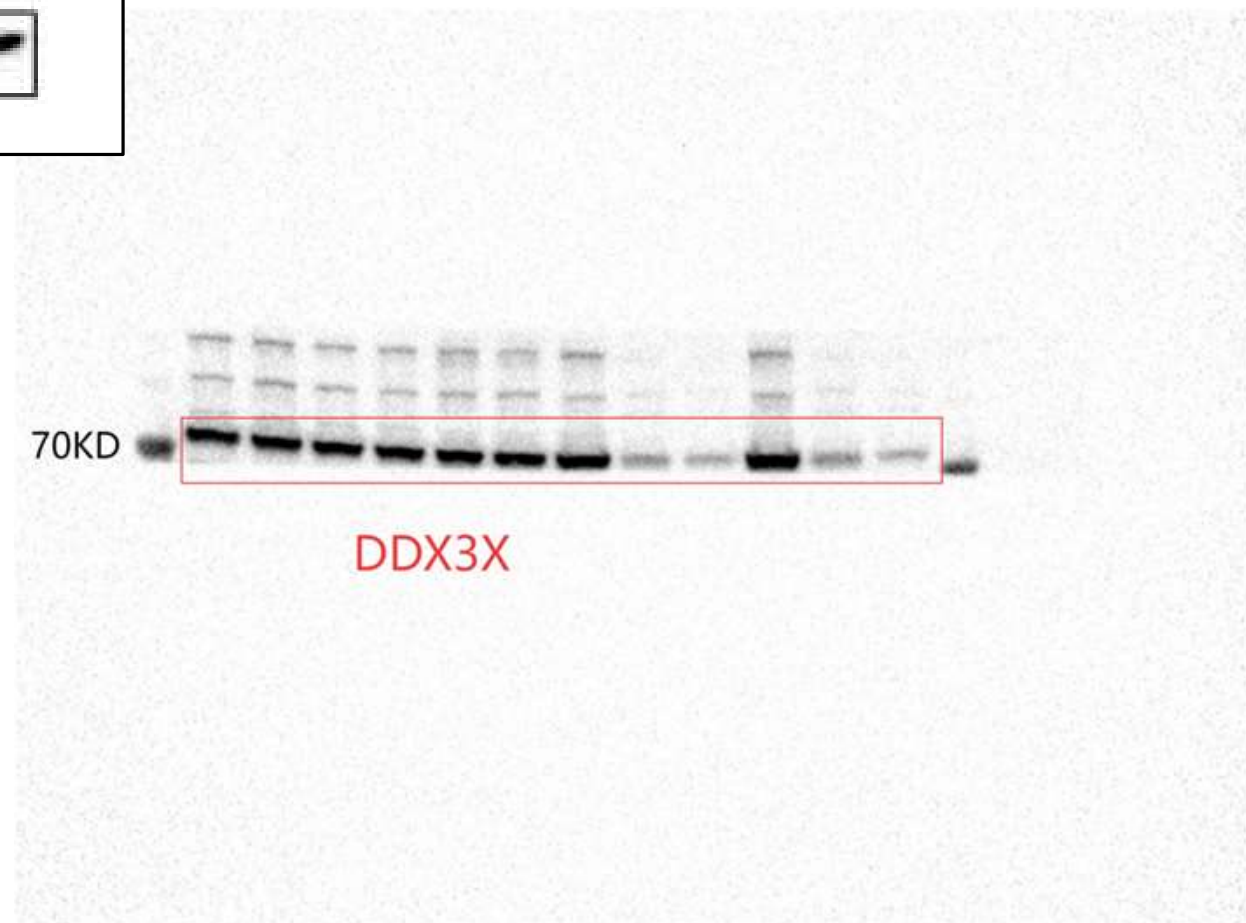
70KD
DDX3X

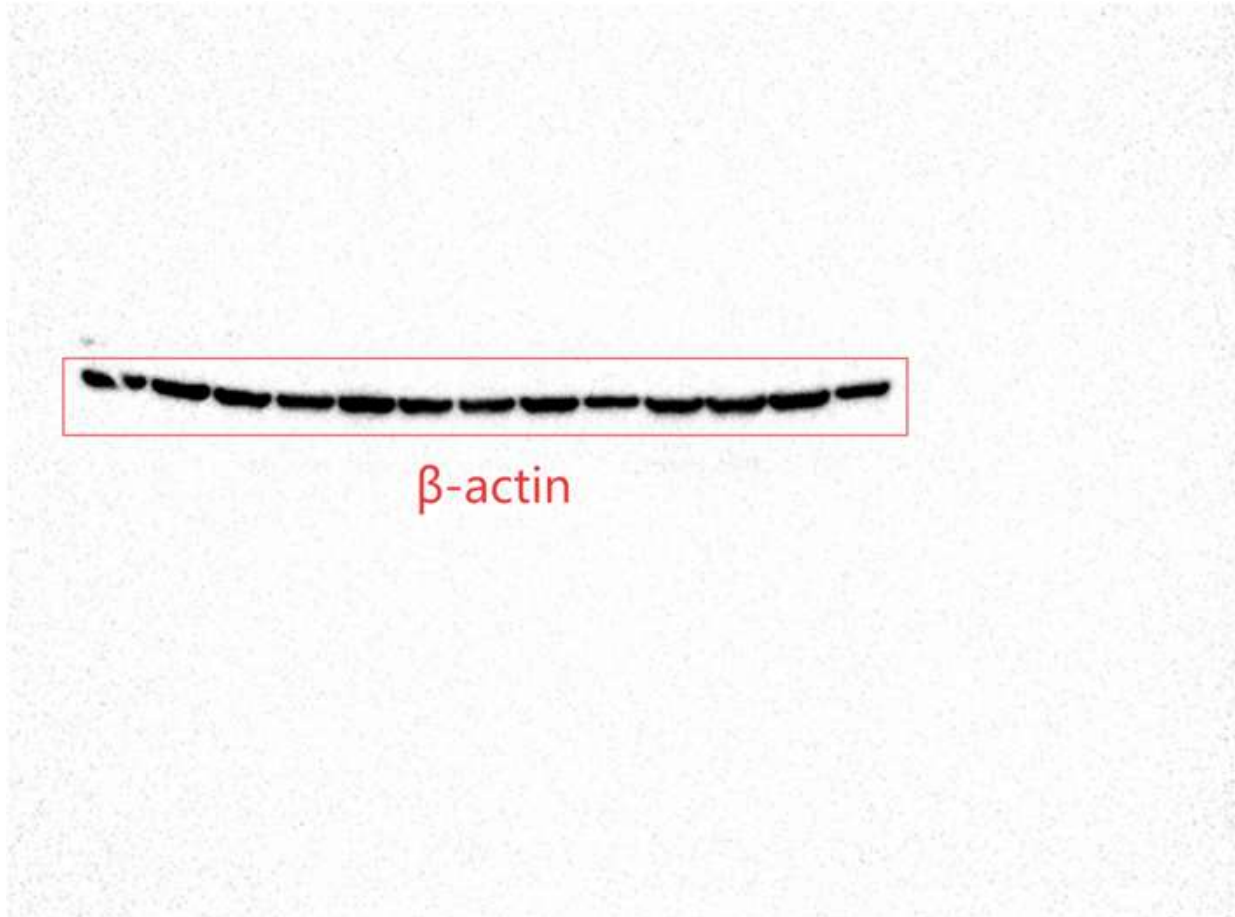
β-actin

Figure S5A

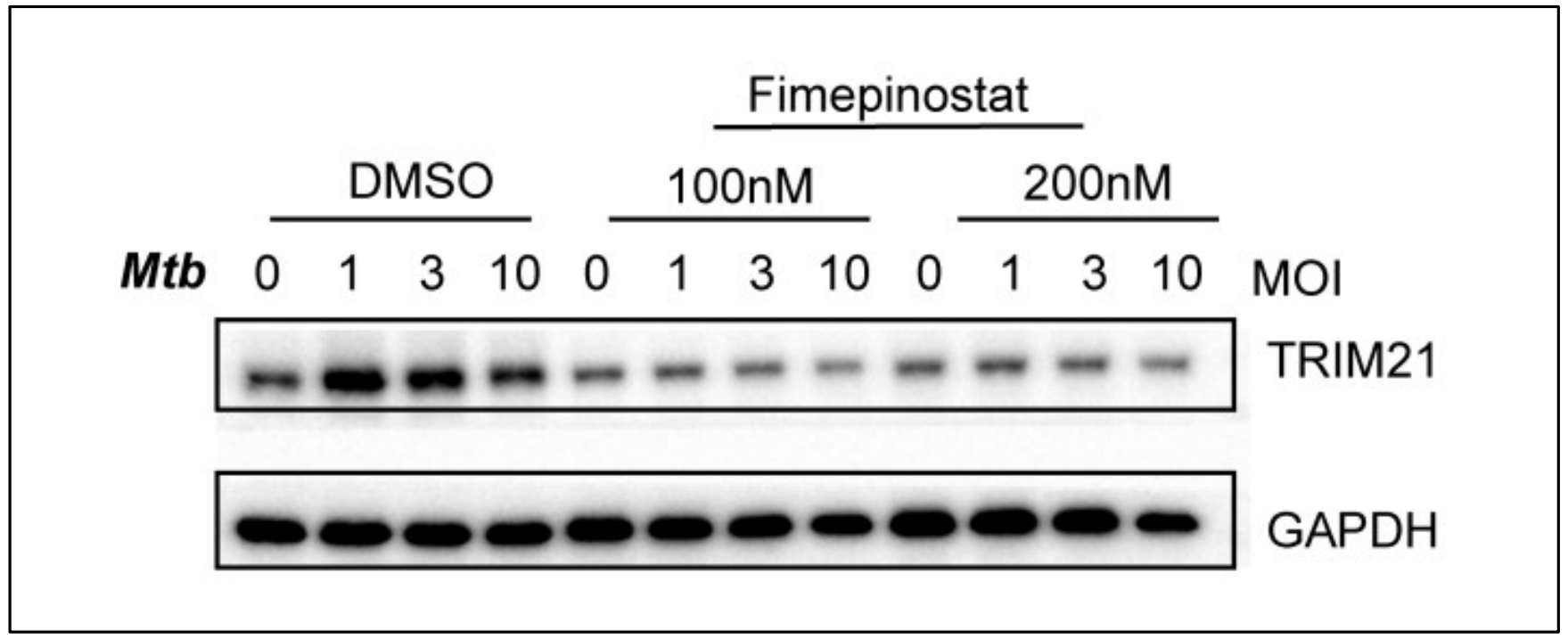
Fimepinostat
DMSO
100nM
200nM
Mtb 0 1 3 10 0 1 3 10 0 1 3 10 MOI
TRIM21
GAPDH

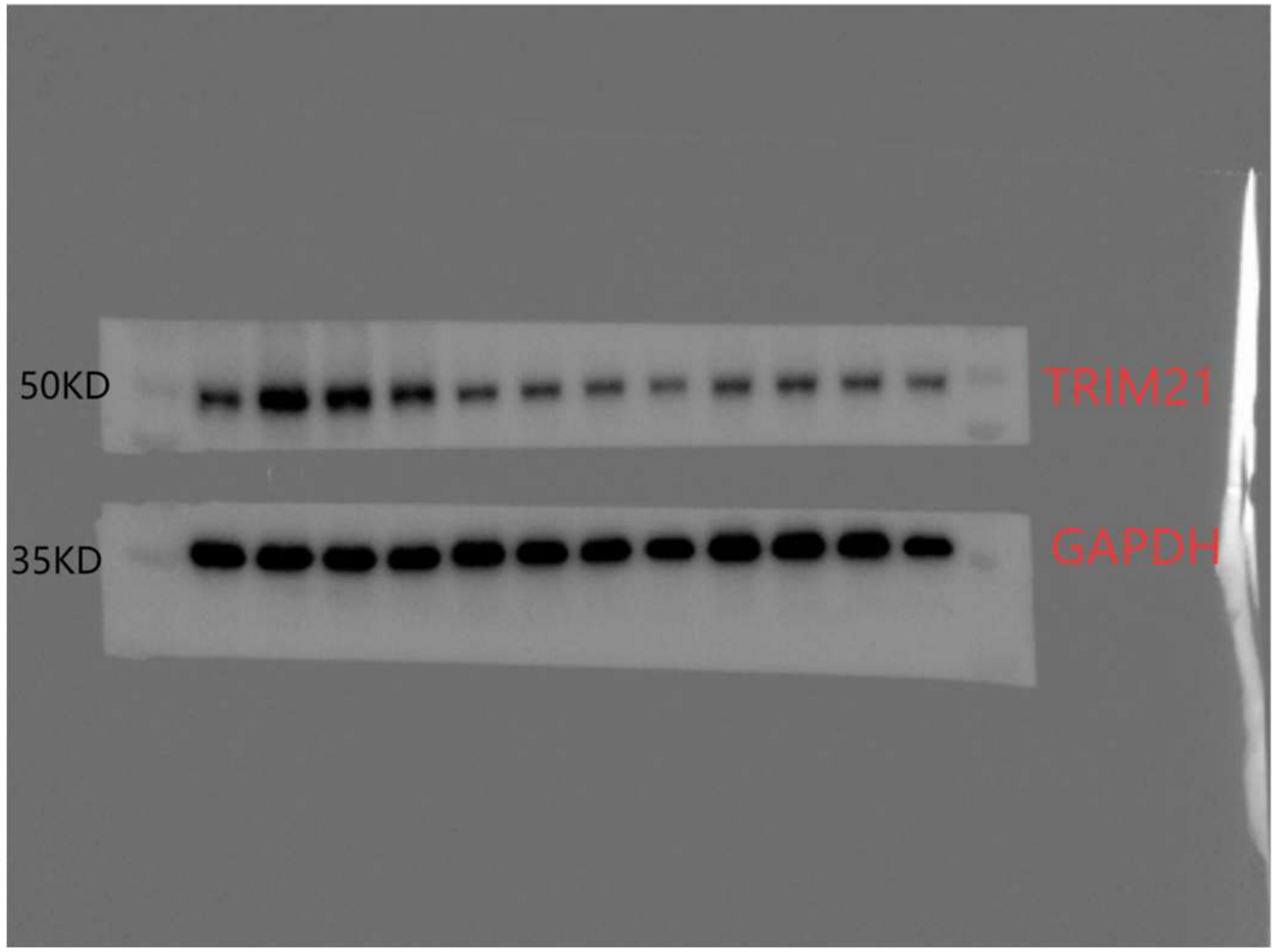
50KD
TRIM21
35KD
GAPDH
